# Global transcriptomic profiling demonstrates induction of oxidative stress and of compensatory cellular stress responses in brown trout exposed to glyphosate and Roundup

**DOI:** 10.1186/s12864-015-1254-5

**Published:** 2015-01-31

**Authors:** Tamsyn M Uren Webster, Eduarda M Santos

**Affiliations:** Biosciences, College of Life & Environmental Sciences, Geoffrey Pope Building, University of Exeter, Exeter, EX4 4QD UK

**Keywords:** RNA-seq, Transcriptome, Herbicide, Salmonids, Toxicogenomics

## Abstract

**Background:**

Glyphosate, the active ingredient in Roundup formulations, is the most widely used herbicide worldwide, and as a result contaminates surface waters and has been detected in food residues, drinking water and human urine, raising concerns for potential environmental and human health impacts. Research has shown that glyphosate and Roundup can induce a broad range of biological effects in exposed organisms, particularly via generation of oxidative stress. However, there has been no comprehensive investigation of the global molecular mechanisms of toxicity of glyphosate and Roundup for any species. We aimed to characterise and compare the global mechanisms of toxicity of glyphosate and Roundup in the liver of brown trout (*Salmo trutta*), an ecologically and economically important vertebrate species, using RNA-seq on an Illumina HiSeq 2500 platform. To do this, we exposed juvenile female brown trout to 0, 0.01, 0.5 and 10 mg/L of glyphosate and Roundup (glyphosate acid equivalent) for 14 days, and sequenced 6 replicate liver samples from each treatment.

**Results:**

We assembled the brown trout transcriptome using an optimised *de novo* approach, and subsequent differential expression analysis identified a total of 1020 differentially-regulated transcripts across all treatments. These included transcripts encoding components of the antioxidant system, a number of stress-response proteins and pro-apoptotic signalling molecules. Functional analysis also revealed over-representation of pathways involved in regulating of cell-proliferation and turnover, and up-regulation of energy metabolism and other metabolic processes.

**Conclusions:**

These transcriptional changes are consistent with generation of oxidative stress and the widespread induction of compensatory cellular stress response pathways. The mechanisms of toxicity identified were similar across both glyphosate and Roundup treatments, including for environmentally relevant concentrations. The significant alterations in transcript expression observed at the lowest concentrations tested raises concerns for the potential toxicity of this herbicide to fish populations inhabiting contaminated rivers.

**Electronic supplementary material:**

The online version of this article (doi:10.1186/s12864-015-1254-5) contains supplementary material, which is available to authorized users.

## Background

Glyphosate is a broad-spectrum, post-emergence herbicide that acts by inhibiting plant aromatic amino acid synthesis via the shikimate pathway [1,2]. In recent years, glyphosate has been the most widely used agricultural herbicide worldwide [3,4], and it is also used extensively in urban and domestic environments [4,5]. Glyphosate can be used alone, but it is more commonly applied as part of a formulated product and the most widely used of these are Roundup herbicides. Roundup formulations vary with application purpose, but contain a number of adjuvants that enhance the herbicidal properties of glyphosate. The most common of these is polyethoxylated tallow amine (POEA), a surfactant that enhances glyphosate cellular uptake in plants [6,7]. Concentrations of glyphosate entering surface waters are not routinely monitored, but values in the range of 10–15 μg/L have been reported in rivers (e.g. [8,9]), while measurements in the high μg/L range have been recorded only occasionally and are generally associated with direct application to wetland environments [7,10]. Glyphosate residues have also been found in food and in drinking water [7], and a recent study reports traces of glyphosate in 44% of human urine samples collected throughout Europe [11]. The widespread use of this herbicide and the measured concentrations in humans and in the environment have raised concerns about its toxicity and the risk that it may pose for human and wildlife health.

Although the target mechanism of action of glyphosate is specific to plants, a range of toxicological effects in a number of vertebrate and invertebrate species have been demonstrated. Both glyphosate and Roundup have been widely shown to induce cellular oxidative stress through generation of ROS and/or interference with the antioxidant system. In fish, short-term exposures to high concentrations (1–20 mg/L) of Roundup altered levels of cellular antioxidants and induced oxidative damage of DNA, lipids and proteins (e.g. [12-14]), while environmentally relevant concentrations of Roundup, glyphosate and POEA caused DNA damage in blood and liver cells of exposed eel and catfish [15-17]. Similarly in rats, treatment with > 100 mg/kg glyphosate and Roundup generated oxidative stress and induced lipid peroxidation [18], and 10 mg/kg glyphosate generated oxidative stress, DNA damage and an increase in apoptosis [19]. In human cell lines both glyphosate (from 50 mg/L) and Roundup (from 18 mg/L) induced apoptosis [20-22], and Roundup also increased necrosis [23,24]. Roundup, and to a lesser extent glyphosate, caused endocrine disruption in cell lines (e.g. [21,25]). Additionally, disruption of steroidogenic enzymes and reproductive health has been demonstrated following Roundup exposure in rats [26,27]. In fish, recent work in our laboratory demonstrated that 10 mg/L of glyphosate and Roundup affect reproduction in zebrafish in a process mediated via disruption of steroid hormone synthesis and induction of oxidative stress [28]. Other demonstrated biological effects of glyphosate and/or its commercial formulations include immunotoxity, neurotoxicity and developmental toxicity [29-31]. Generally, Roundup has been found to be more toxic than pure glyphosate, and this has been attributed to the inherent toxicity of POEA [16,30], and potentially other formulation products. Additionally, formulation products may enhance the toxicity of glyphosate by facilitating cellular entry [18].

Transcriptional profiling can be employed to comprehensively investigate global molecular mechanisms of chemical toxicity which, in turn, this can potentially provide valuable information for understanding and predicting adverse health effects following environmental exposure. Microarray technology has been successfully used to conduct transcriptomic analysis in fish and other aquatic organisms exposed to a wide range of environmental chemical pollutants, but the application of this technology required significant prior gene sequence information. High-throughput RNA sequencing (RNA-seq) has recently emerged as a sensitive and reproducible tool, and can be used to conduct non-biased transcriptomic analysis in any species of interest. In ecotoxicology, RNA-seq provides a valuable opportunity to investigate global mechanistic toxicology in environmentally-relevant species, but is yet to be widely employed in this field.

Despite the high rate of glyphosate usage and the concerns about its potential to cause human and environmental health impacts, no comprehensive studies investigating the global mechanisms of toxicity of glyphosate and its commercial formulation, Roundup, have been performed to date. This study aimed to investigate and compare the global transcriptional responses to glyphosate and Roundup in brown trout (*Salmo trutta*). Brown trout are an ecologically and economically important European species, known to be sensitive to environmental stressors. Due to their ecological niche, brown trout are likely to be affected by these compounds, particularly as a result of agricultural runoff. We conducted an exposure of juvenile female brown trout to three concentrations of both glyphosate and Roundup, including environmentally relevant concentrations and investigated the toxicological effects of these compounds in the liver of exposed fish using RNA-seq.

## Methods

### Fish maintenance

Juvenile brown trout (six months old; originating from a local aquaculture facility) were maintained in 35 L glass tanks, and acclimated to laboratory conditions for three weeks prior to the start of the exposure. Each tank was aerated and supplied with a water flow rate of 140 L/day. The aquarium water supply was reverse-osmosis treated tap water reconstituted with analar-grade salts to produce a standardized synthetic freshwater according to OECD guidelines, as described in Paull et al. [32], and maintained at 12 ± 0.2°C and pH 7.2-7.8. Fish were kept under a 16:8 h light:dark cycle (with 30 minute dawn/dusk transitional periods) and fed with 0.5 mm trout pellets (Biomar, Grangemouth, UK) at a rate of 2% body weight per day.

### Chemical exposure and sampling

All experiments were conducted under approved protocols according to the UK Home Office regulations for use of animals in scientific procedures, and approved by the University of Exeter Ethics committee.

Chemical exposure was conducted via a flow through system for a period of 14 days, employing a flow rate of 140 L/day for each tank (4x tank volume per day). The treatment groups consisted of three concentrations of glyphosate; 0.01, 0.5 and 10 mg/L (analytical grade; Molekula, Wimborne, UK), three concentrations of Roundup; 0.01, 0.5 and 10 mg/L glyphosate acid equivalent (using Roundup® GC liquid glyphosate concentrate containing 120 g/L glyphosate acid; Monsanto, Cambridge, UK); and a control group. These treatment groups will be referred to throughout as LG, MG, HG and LR, MR, HR for the 0.01, 0.5 and 10 mg/L Glyphosate and Roundup treatments, respectively. The two lower concentrations were chosen to represent concentrations that may occur in the environment frequently (0.01 mg/L) or during occasional peak contamination events (0.5 mg/L). The highest concentration tested (10 mg/L) was included to facilitate the analysis of the mechanisms of toxicity of glyphosate and Roundup but is unlikely to occur in surface waters. Each treatment group was comprised of two replicate tanks, with a volume of 35 L, each containing 8 fish. Water samples were collected from each tank on day 0, 7 and 14 of the exposure period and stored at −20°C prior to chemical analysis. Samples from the three separate time points were combined into a single sample for each replicate tank and analysed for the concentrations of glyphosate in an external accredited laboratory using LC-MS (South West Water, Exeter Laboratories).

Fish were humanely sacrificed on day 14 of the exposure period by a lethal dose of benzocaine (0.5 g L-1; Sigma-Aldrich) followed by destruction of the brain, in accordance with UK Home Office regulations. Wet weight and fork length were recorded, and the condition factor (k = (weight (g) × 100) / (fork length (cm)^3^)) was calculated for individual fish. Sex was determined by visual observation of the gonads. Livers were dissected and weighed, and the hepatosomatic index (HSI) (liver weight (mg)/total weight (mg)) × 100)) was determined for individual fish. Portions of the liver from female fish were snap frozen in liquid nitrogen and stored at −80°C prior to transcript profiling.

### RNA extraction, library preparation and sequencing

Transcript profiling was conducted in the liver of 6 females per treatment group. For the MG treatment group, only 3 individuals were analysed because there were only three females in the replicate tanks for this treatment. RNA was extracted from female livers using an RNeasy Mini extraction kit (Qiagen), incorporating on-column DNase treatment, according to the manufacturer’s instructions. The concentration, purity and integrity of RNA were determined using a NanoDrop ND-1000 Spectrophotometer (NanoDrop Technologies, USA) and an Agilent 2100 Bioanalyzer (Agilent Technologies, Inc., USA). All RNA input to library construction was of high quality with A_260_/A_280_ and A_260_/A_230_ ratios > 1.8 and RIN scores > 8. ERCC spike-in control mixes (Ambion) were added to all individual RNA samples, according to the manufacturer’s instructions to allow for analysis of the accuracy of the transcript quantification and dynamic range. cDNA libraries from all samples were then prepared using the Illumina TruSeq RNA Sample Preparation kit, multiplexed with 24 samples per lane and sequenced using an Illumina HiSeq 2500, to generate 100 bp paired reads.

### Transcriptome assembly and annotation

All analyses were carried out on a local server running the NEBC Bio-Linux 7 environment [23]. Remaining Illumina adaptor sequences were removed and the first 12 bp of all raw sequence reads were trimmed to remove 5' bias caused by non-random hexamer priming [33] using the FASTX-Toolkit (http://hannonlab.cshl.edu/fastx_toolkit). 3' sliding window quality trimming was performed using (http://wiki.bioinformatics.ucdavis.edu/index.php/Trim.slidingWindow.pl) and all reads where < 90% bases had a Phred quality score >20, and those shorter than 15 bp, were discarded. Digital normalisation was performed to remove highly duplicated reads using the normalize-by-median.py script part of the khmer package described by Brown et al. [34], with the recommended k-mer value of 20 and a coverage threshold of 20. This process reduces the computer memory requirements of transcriptome assembly, and also reduces the risk of potential sequencing error accumulation in abundant transcripts [35]. All retained reads were then paired, separated into forward and reverse fastq files for Trinity assembly and shuffled into a single interleaved fastq file for the Velvet assembly. In order to obtain the most appropriate transcriptome assembly for downstream expression analysis, we conducted *de novo* transcriptome assemblies using Velvet (version 1.2.08; [36]) followed by Oases (version 0.2.08; [37]), and Trinity (version r2013-02-25; [38]), and compared them. The Trinity assembly was conducted using the default parameters, specifying a minimum contig length of 200 bp. A series of 7 separate Velvet-Oases assemblies were created specifying ins_length 161 -ins_length_sd 150 and using k-mers ranging from 33 to 69 (with steps of 6), then these were merged using the Oases-Merge function (K = 27) specifying -min_trans_lgth 200. All transcripts in the final assemblies were annotated using Blastx against the Ensembl peptide databases (Release 71; April 2013) using an E-value cut-off of 10^−15^ and ‘best hits’ were assigned to transcripts in the following preferential order: zebrafish (*Danio rerio*); human (*Homo sapiens*) and mouse (*Mus musculus*); stickleback (*Gasterosteus aculeatus*), medaka (*Oryzias latipes*), tilapia (*Oreochromis niloticus*) and cod (*Gadus morhua*). For transcripts found to be differentially expressed (see below) that were not annotated in this way, additional annotation was performed using Blast (<10^−15^) against the NCBI Reference Sequence (RefSeq), nr and nt databases.

### Transcriptomic analysis

Raw sequence reads from individual samples were mapped back against both Trinity and Oases transcriptome assemblies using Bowtie2 (version 2.1.0, [39]), using a value of 1 for the k parameter (−k 1) to report a single best hit for each read and limit ambiguous mapping to redundant transcripts. Raw count data for each transcript was extracted using idxstats in SAMtools (version 0.1.18, [40]) and input into the edgeR R package [41] for differential expression analysis. In edgeR, a criterion of having at least 1 mapped read from a minimum of 6 samples for each transcript was imposed. Tagwise dispersion was applied with the recommended prior.df = 10. Initial comparison of transcript expression between the two control treatment groups showed strong similarities, and only 3 and 5 differentially expressed transcripts were identified for the Trinity and Oases assemblies, respectively. Therefore, differential expression analysis was conducted between the 12 replicates from the combined control groups and six replicates from each of the other treatment groups. Transcripts were considered differentially expressed with a FDR < 0.1 (after Benjamini-Hochberg correction). Hierarchical clustering was performed on all differentially expressed transcripts using an Euclidean distance metric, in the Pheatmap package for R (available from http://cran.r-project.org/web/packages/pheatmap/index.html). Functional analysis was then performed for differentially expressed genes from each treatment using the Database for Annotation, Visualisation and Integrated Discovery (DAVID v6.7; [42]), with the final brown trout liver transcriptome as a background. Kyoto Encyclopedia of Genes and Genomes (Kegg) pathways (http://www.genome.jp/kegg/) and Gene Ontology (GO) terms (http://geneontology.org/) for Biological Process, Cellular Component and Molecular Function were considered significantly over-represented when P < 0.05.

The raw sequence data, and processed results from the expression analysis have been deposited in NCBI’s Gene Expression Omnibus (http://www.ncbi.nlm.nih.gov/geo), and are available via the GEO series accession number GSE56855.

## Results and discussion

### Water chemistry and morphometric parameters

The concentration of glyphosate in each tank was measured in a composite water sample from three separate time points during the exposure (days 0, 7 and 14), and were within 88.7-110.7% of the nominal concentrations across all treatments. The mean measured concentrations for each treatment (2 replicate tanks) were 0.0097 ± 0.0003; 0.46 ± 0.01 and 9.9 ± 0.41 mg glyphosate/L for the 0.01, 0.5 and 10 mg glyphosate/L treatment groups respectively, and 0.011 ± 0.0003; 0.50 ± 0.007; 9.31 ± 0.17 mg glyphosate/L for the 0.01, 0.5 and 10 mg Roundup/L treatment groups, respectively.

The mean mass and mean length was 13.1 ± 0.3 g and 9.97 ± 0.09 cm for female fish and 12.54 ± 0.37 g and 9.75 ± 0.09 cm for male fish. There were no significant differences in size and condition factor (mean 1.31 female; mean 1.33 male) or HSI (mean 1.66 female; mean 1.75 male) between treatment groups. There were no mortalities and we observed no alteration of behaviour during the course of the exposure, suggesting that these concentrations of glyphosate and Roundup had no overt toxicological effects on general health of the exposed fish.

### Sequencing and *de novo* transcriptome assembly

Sequencing of female liver samples generated a total of 969.4 million paired 100 bp reads, averaging 20.2 million reads per library, 92.5% (897 million) of which were retained following processing and quality filtering. Following digital normalisation, a total of 101.5 million paired reads, originating from all libraries, were retained and input into the *de novo* transcriptome assemblies. Statistics for the Trinity and Velvet-Oases transcriptome assemblies are shown in Additional file 1: Table S1. The final Velvet-Oases assembly consisted of 893,904 transcripts compared to 258,702 in the Trinity assembly, while the number of putative gene loci was more similar (146,233 and 109,301 respectively). Transcript length statistics and the percentages of transcripts annotated using Blastx against Ensembl peptide databases were similar in both assemblies (45% of Trinity transcripts and 47% of Oases transcripts). There was a higher rate redundancy amongst annotated Oases transcripts, although this assembly included representation of over 2000 more unique transcripts (based on the annotation against the Ensembl zebrafish database). Overall this indicates that Velvet-Oases produced a considerably more redundant assembly, but potentially included better coverage of the brown trout liver transcriptome. Previously, Velvet-Oases transcriptome assemblies have also been found to be more redundant than Trinity assemblies [43]. For species without a good quality reference genome, like the brown trout, the quality and reliability of transcript expression analysis using RNA-seq are dependent on the quality of the *de novo* transcriptome assembly. This can be assessed using a number of metrics, which may vary based on the dataset and study objectives. Transcript redundancy is an important measure of assembly quality because high levels of redundancy tend to increase levels of ambiguous read mapping and reduce statistical power in differential expression analysis. However, inclusion of the greatest number of unique gene isoforms, including rare transcripts, is important for subsequent expression analysis and biological interpretation. Both the Trinity and Oases assemblies, therefore, have advantageous characteristics, so we performed the differential expression analysis for both assemblies independently, followed by a comparison of the resulting gene lists.

### Transcript expression analysis

A greater percentage of reads were mapped to the Oases assembly compared to the Trinity assembly (mean 94% and 90% per sample, respectively) using Bowtie2. However, for the Trinity assembly, a greater percentage of transcripts met the criterion of having at least one mapped read in six replicate samples and were retained for differential expression analysis in edgeR. The retained transcripts from the Trinity assembly also included representation of 32% more of the transcripts in the Ensembl zebrafish database. Additionally, calculated values of biological coefficient of variation (BCV) for comparisons across all treatments were consistently lower using the Trinity assembly (average 34.6%) compared to the Oases assembly (average 37.4%). Furthermore, there was a lower degree of redundancy in the list of annotated differentially expressed transcripts identified using the Trinity assembly. These differences likely reflect the greater degree of transcript redundancy in the Oases transcriptome assembly, and together, indicate that the Trinity assembly, for the present dataset, was of higher quality for transcript expression analysis. Therefore, we used the results obtained using the Trinity assembly for further biological interpretation and functional analysis.

The ERCC spike-in control analysis for all individual samples is presented in Table 1, and in Additional file 1: Figures S4 and S5. For all samples there was a strong correlation between calculated FPKM value and expected concentration of spiked-transcripts (mean R^2^ = 0.918 ± 0.002), and the mean calculated dynamic range in expression level was 25,722 FPKM. There was also a strong correlation between calculated and expected fold changes in transcript expression between samples spiked with ERCC mix 1 and ERCC mix 2 (p = 1.5E-18, R^2^ = 0.6223). Together these results provide strong technical validation for the quantitative transcript profile analysis presented here.

**Table 1:** **ERCC spike-in control analysis for assessment of transcript expression reproducibility and dynamic range in all individual samples sequenced**

**Sample**	**R** ^**2**^	**Log2 dynamic range (FPKM)**	**Sample**	**R** ^**2**^	**Log2 dynamic range (FPKM)**	**Sample**	**R** ^**2**^	**Log2 dynamic range (FPKM)**
C1	0.93	13.74	LG1	0.92	14.36	LR1	0.93	14.91
C2	0.91	14.52	LG2	0.91	14.52	LR2	0.91	14.64
C3	0.92	14.54	LG3	0.91	15.16	LR3	0.92	13.71
C4	0.94	15.61	LG4	0.91	15.32	LR4	0.93	13.32
C5	0.94	14.21	LG5	0.92	13.92	LR5	0.91	14.53
C6	0.94	14.29	LG6	0.90	14.49	LR6	0.93	16.05
C7	0.92	13.88	HG1	0.90	14.18	MR1	0.91	14.00
C8	0.92	13.83	HG2	0.91	15.26	MR2	0.93	13.97
C9	0.89	14.11	HG3	0.93	15.51	MR3	0.91	15.60
C10	0.92	14.28	HG4	0.90	14.99	MR4	0.92	14.07
C11	0.91	16.19	HG5	0.90	14.92	MR5	0.93	13.86
C12	0.93	15.26	HG6	0.93	14.76	MR6	0.92	15.70
						HR1	0.91	16.08
						HR2	0.90	13.95
						HR3	0.92	14.08
						HR4	0.91	14.08
						HR5	0.93	15.75
						HR6	0.92	15.19

1. R^2^ values represent the correlation between measured FPKM values and expected concentrations of control transcripts.

2. Dynamic range was calculated from maximum-minimum FPKM values of control transcripts for all individual libraries, using only control transcripts that had ≥1 mapped read in ≥6 replicate samples.

The MG treatment group, which had only three replicates, had the highest BCV value of all treatment groups, and multidimensional scaling (MDS) plots show that there was one individual in this group with a very different transcript profile compared to the other replicates (Additional file 1: Figure S1). Transcript expression analysis revealed an unrealistically high number of differentially expressed transcripts in this group compared to the control (>5000), presumably because of the strong influence of this individual in a group with few replicates, which increased biological variation and potentially false positive discovery. This treatment group was therefore excluded from the functional analysis.

**Figure 1:**
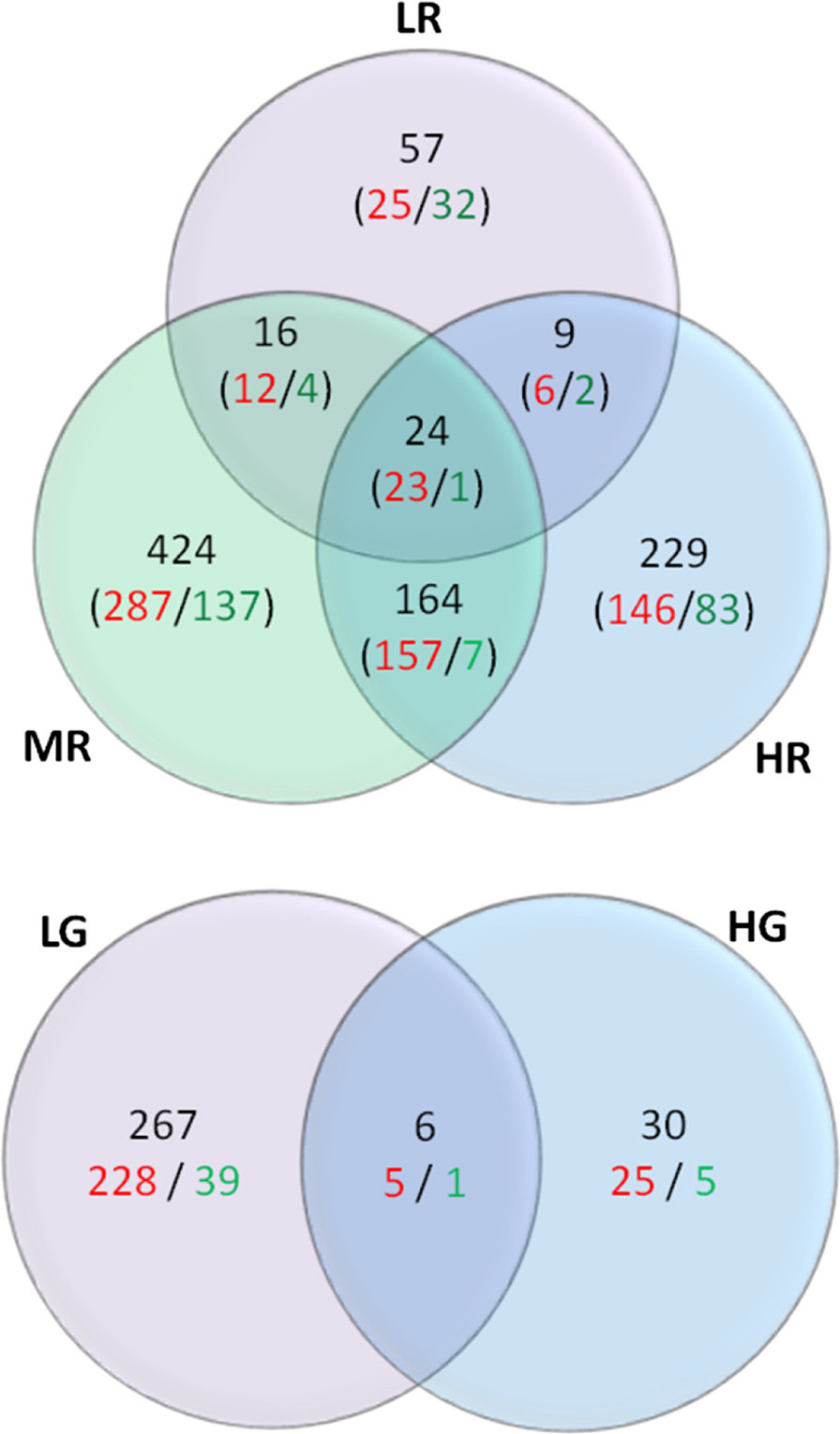
**Venn diagrams displaying the numbers of differentially expressed transcripts (FDR < 0.1) in each treatment group obtained from edgeR using the Trinity assembly as a template.** Red and green numbers represent up- and down-regulated transcripts, respectively. Treatments are represented by the following codes: LR, MR and HR represent 0.01, 0.5 and 10 mg/L Roundup, and LG and HG represent 0.01 and 10 mg/L glyphosate.

The numbers of up- and down-regulated transcripts in each treatment group, including overlaps between treatment groups, using the Trinity assembly are shown in Figure 1. The transcript level expression plots for each treatment are shown in Additional file 1: Figure S2 and the full list of differentially expressed transcripts are presented in Additional file 1: Table S3. The total number of transcripts differentially expressed in one or more Roundup treatment groups compared to the controls was 923 (656 of which were up-regulated; 266 were down-regulated; and 1 was up and down regulated in the LR and HR groups, respectively) and in the two glyphosate-treated groups was 303 (258 of which were up-regulated and 45 were down-regulated). Of these, 143 transcripts were differentially regulated following exposure to both glyphosate and Roundup (135 transcripts were up-regulated and 8 transcripts were down-regulated). The results of the differential expression analysis using the Oases assembly show a similar pattern in the number of differentially expressed transcripts in each treatment group, including a predominance of up-regulation, which increases our confidence in functional analysis and biological interpretation of the data. These results, from the Oases assembly, are presented in the (Additional file 1: Figure S3).

**Figure 2:**
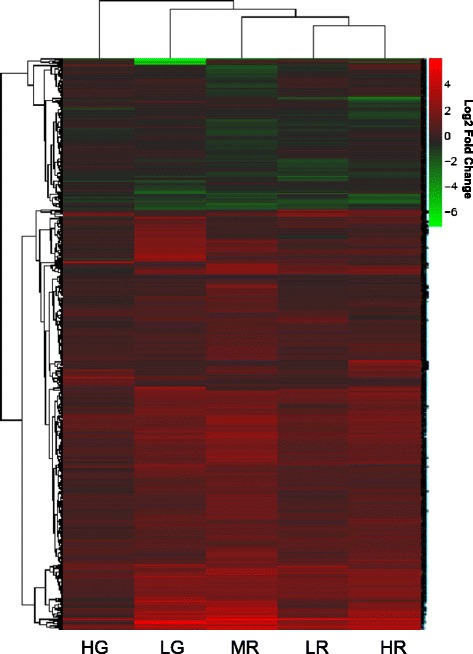
**Heatmap illustrating changes in expression level of all differentially expressed transcripts across treatment groups.** Data presented are the mean log2 fold change in expression level in each treatment group compared to the control. Hierarchical trees were generated using an Euclidean distance metric. Treatments are represented by the following codes: LR, MR and HR represent 0.01, 0.5 and 10 mg/L Roundup, and LG and HG represent 0.01 and 10 mg/L glyphosate.

Cluster analysis of all 1020 differentially expressed transcripts showed that there were strong similarities in the expression profiles of all Roundup treatment groups and the LG group (Figure 2). Visual examination of this cluster diagram shows that across these treatment groups the majority of transcripts displayed similar expression trends, even when no significant changes were detected. The three Roundup treatments clustered more closely together than to the glyphosate treatments. This is likely to reflect differences between glyphosate and Roundup due to the presence of surfactants, including POEA, which are likely to enhance and/or modify the toxicity of the formulation product, or exert some toxicological effects independently from glyphosate. The expression profile of the LG treatment was also broadly similar to that of the Roundup treatments, and functional analysis confirmed an overlap in gene ontologies and signalling pathways affected (discussed below). This suggests that the toxicological responses to Roundup and glyphosate occur via shared mechanisms of toxicity.

The differentially regulated transcript profile for fish exposed to HG was relatively distinct from the other treatments, and there were also considerably fewer differentially expressed transcripts in this group (Figures 1, 2). The reasons for this are not clear, but technical differences in the sequencing or water chemistry, are unlikely to contribute to this outcome, due to the random assignment of samples during the library preparation and sequencing procedures and the very consistent measurement of glyphosate in the HG treatments (95% and 103% of nominal concentration for the two replicate tanks). The consistency of expression profiles between individual fish in the HG group (Additional file 1: Figure S1) also suggests that high biological variation is unlikely to have limited statistical power for identifying differentially expressed transcripts. It is possible that different mechanisms of toxicity were induced by the LG and HG treatments, although the large difference in concentration between these treatments makes it difficult to draw firm conclusions. Non-monotonic dose response curves have been reported for a number of different environmental chemicals including Bisphenol A and DEHP [44,45]. For glyphosate and Roundup, different mechanisms of toxicity at different treatment concentrations have also been reported in cell line exposures including more acutely toxic effects, such as necrosis, at the highest treatment concentrations [20,21,23]. Following functional analysis, which is discussed below, we found that the transcript expression changes in the Roundup and LG treatments were characteristic of an extensive compensatory cellular stress response. Therefore, we hypothesise that in the HG treatment this response may have been depressed, potentially suggesting more acute toxicological effects at this concentration, and resulting in the distinct expression profile observed. It is also possible that a similar response occurred in Roundup-treated fish, as fewer changes in transcript expression were induced by the highest treatment concentration (Figure 1). Furthermore, herbicides are known to significantly modify the algal and microbial composition of an environmental or experimental system [46], potentially exerting indirect effects on exposed fish, and contributing to the different expression profiles observed.

### Functional analysis and biological interpretation

Functional analysis of differentially expressed transcripts identified 68 over-represented GO terms (Biological Process, Molecular Function and Cellular Compartment), and 11 over-represented Kegg pathways (p < 0.05), across all treatments (Additional file 1: Table S2).

#### Oxidative stress, cellular stress response and apoptosis

We found evidence suggesting an up-regulation of the antioxidant system following both Roundup and glyphosate exposure. Glutathione reductase (*gsr*) was significantly up-regulated by MR, and there were increasing trends in expression of transcripts encoding for this enzyme in the other treatment groups. This key enzyme is responsible for the restoration of reduced glutathione (GSH), a major cellular antioxidant which neutralises ROS, and in the process is itself oxidised [47]. Additionally, three transcripts encoding heme oxygenase 1 (*hmox1*) were differentially-regulated by LG and MR. These proteins have roles as cellular anti-oxidants and in the maintenance of cellular redox balance, and have previously been shown to be amongst the most responsive markers of cellular oxidative stress [48,49]. Our data is therefore consistent with previous reports that glyphosate and Roundup induce oxidative stress in fish and other species, including at concentrations measured in the environment, potentially resulting in damage of DNA, lipids and proteins, and modulation of the cellular antioxidant system (e.g. [12-17]).

A number of transcripts encoding stress-response proteins were differentially expressed. These included hypoxia induced gene 1 (*hig1*) and hypoxia up-regulated protein 1 (*hyou1*) which were significantly up-regulated in the MR and LG treatments, respectively, and also showed increasing trends in expression in the other treatment groups. Heat shock proteins (encoded by genes *hsp5, hsp13, hspb2, dnajb11, dnajb9, dnajc3*), which bind, stabilise and remove damaged proteins, were also differentially regulated across various treatment groups. The transcription factor tumour-suppressor protein p53 (*tp53*) was up-regulated in the LG treatment. p53 transcription has been previously associated with oxidative stress-induced genotoxicity, and mediates several cellular stress responses including arrest in the cell cycle, preventing mutation propagation, initiation of DNA repair mechanisms and initiation of apoptosis when DNA damage is extensive [47,50,51]. We also found evidence of differential regulation of transcripts involved in DNA-repair. For example, DNA damage-inducible transcripts (*ddit4, ddit4l*) were up-regulated by HG and LG, respectively, and DNA damage-inducible protein 4a (*gadd45a*) was down-regulated in MR, HR and LG.

Cellular stress response is mediated by a diverse array of interacting signalling pathways, and the nature of the response can vary depending on the degree and duration of the stress. For example, low concentrations of ROS tend to induce pro-survival signalling, while a greater degree of oxidative stress and cellular damage can promote apoptosis as a protective mechanism [47]. We found evidence of up-regulation of several of the major regulatory pathways responsible for cellular stress response and apoptosis following exposure to both glyphosate and Roundup, including mitogen-activated protein kinase (MAPK), tumour necrosis factor (TNF) and calcium signalling. MAPK signalling was over-represented in both the MR and LG treatments (Additional file 1: Table S2) where transcripts encoding p38- and JNK-related MAPK proteins (*mapk14a, mapk14b*, *mapk3k6*) were up-regulated. Additionally, a number of transcripts encoding MAPK-interacting serine/threonine kinases (*mknk1* and *mknk2b*) were up-regulated across LR, MR, HR and LG treatments. TNF signalling-related transcripts were also up-regulated including *tnfr14* (LR, MR, HR, LG), *tnip3, optn, sqstm1* (MR, HR, LG), *tnip2* (MR, HR) and *tnfr2, cd40*, *traf3, traf2b* (MR). TNF signalling has previously been reported to be modulated in rats exposed to both glyphosate and Roundup, in association with oxidative stress [18]. Calcium signalling was over-represented in fish exposed to LG, and transcripts with roles in maintaining calcium homeostasis were significantly up-regulated including Ca^2+^ transporting ATPases (*atp2a2a, atp2a2b*) in LG and MR, with increasing trends in the other treatment groups. Calsynterin (*clstn1*), calcitonin receptor (*calcrla*) and tescalcin (*tescb*) were also up-regulated by LG, and calcineurin-like (*chp1*) was up-regulated by MR. Calcium channel (*cacng6b)* was down-regulated by LR, while hippocalcin (*hpcl1*) was down-regulated by MR and HR. Additionally, transcripts encoding components of transcription factors known to be induced by ROS and important in the regulation of cellular stress response and apoptosis were differentially expressed. In particular, Fos-like antigen 2 (*fosl2*) and activating transcription factor 3 (*atf3*), which are key components of the transcription factor AP-1, were some of the most up-regulated transcripts across MR,HR and LG treatments, by 4.6-6.4 and 3.9-5.4 fold respectively. In addition, three key members of the nuclear factor kappa N (NF-κB) family (*nfkb2, rela, relb*) were up-regulated in fish across LR, MR, HR and LG treatments.

**Figure 3:**
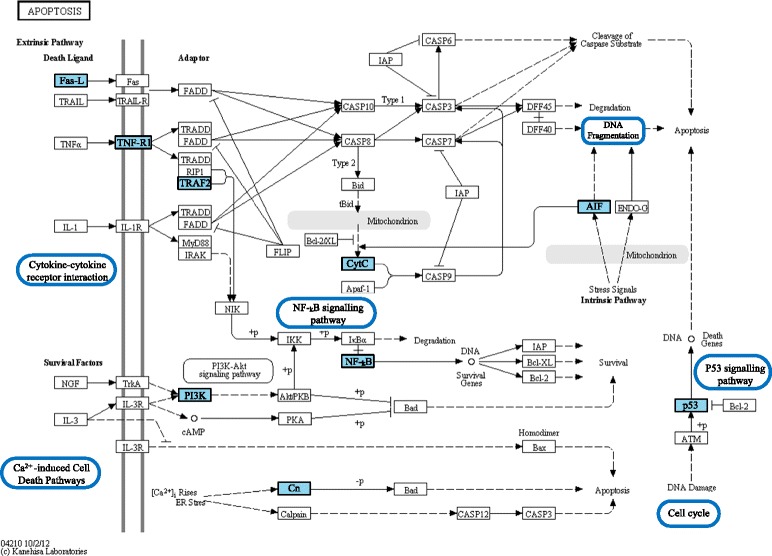
**Kegg pathway representing** ***Apoptosis*** **, which was found to be over-represented in the list of differentially-regulated transcripts across treatment groups.** Analysis was conducted using the Database for Annotation, Visualization and Integrated Discovery (DAVID)^42^ v6.7, using the *de novo* assembled liver transcriptome generated in this study as a background. Differentially expressed transcripts and enriched related processes from all glyphosate and Roundup treatments are highlighted in blue.

The *apoptosis* Kegg pathway displaying a summary of differentially regulated genes and processes across treatment groups is shown in Figure 3. In addition to the signalling pathways discussed above that can regulate apoptosis in a positive or negative way, transcript profiling also revealed up-regulation of a number of factors that specifically promote apoptosis. Mitochondrion-associated apoptosis-inducing factor (*aifm2*), an intrinsic signalling factor controlling apoptosis, was significantly up-regulated by MR treatment and there were increasing trends in all other treatment groups, while caspase recruitment domain-containing protein 14 (*card14*), which interacts with members of the Bcl family, and is a positive regulator of apoptosis, was up-regulated by MR and HR. With regard to the extrinsic signalling control of apoptosis, two transcripts encoding lymphocyte G0/G1 switch protein 2 (*G0S2*) were amongst the most strongly up-regulated transcripts (7–25 fold by MR, HR and LG treatments). This gene has been found to strongly promote apoptosis by binding Bcl-2 and inhibiting its anti-apoptotic activity, through induction by TNF signalling and NF-kB activity [52,53]. In addition, programmed cell death protein 6 (*pcdp6*) and cytotoxic granule-associated RNA binding protein (*tia1*), which are also pro-apoptotic factors and interact with the TNF family Fas-receptor, were up-regulated by MR. Transcripts encoding sphingomyelin synthase 2 (*sgms2*) and sphingomyelin phosphodiesterase 5 (*smpd5*), which are both important in the generation of pro-apoptotic ceramide, were up-regulated by MR, and by MR and HR, respectively. These results align strongly with previous research, where glyphosate and various Roundup formulations have been shown to cause an increase in the rate of apoptosis in various human cell lines, characterised by elevated caspase activity [20,21,23,24], altered Bcl protein activity and loss of mitochondrial integrity [22]. Additionally, we found some evidence of an increase in autophagy, another form of programmed cell death, which is regulated by many of the same pathways that regulate apoptosis [53]. Autophagy related homolog 5 (*atg5*) was amongst the most up-regulated transcripts (5–35 fold) by MR, HR and LG treatments.

**Figure 4:**
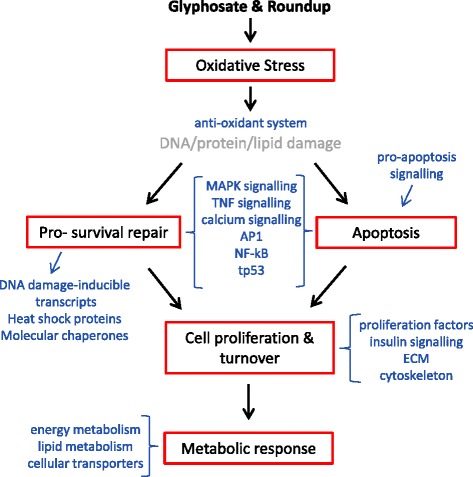
**Schematic illustrating oxidative stress as a central mechanism of glyphosate and Roundup toxicity, and the compensatory processes affected which are consistent with an associated cellular stress response in brown trout.** Blue text indicates the key regulated signalling pathways and functional gene groups associated with each process.

Overall, these data indicate that both glyphosate and Roundup induced cellular stress response mechanisms. We hypothesise that this resulted from the generation of oxidative stress, and this hypothesis is summarised in the schematic pathway presented in Figure 4. There was some evidence of differential modulation of the regulatory signalling pathways between treatment groups which may reflect dose-specific effects, including a differential balance between pro-survival and pro-apoptotic pathways following exposures to low concentrations versus high concentrations of Roundup and glyphosate. Specifically, transcriptional changes suggest a shift towards pro-apoptotic cellular stress response pathways in fish exposed to MR and HR, and to a lesser extent, LG. Although apoptosis-regulating pathways were affected by LR exposure, we found fewer changes in expression of pro-apoptosis factors than in the other treatment groups. This may suggest a dominance of pro-survival stress response mechanisms at lower treatment concentrations of Roundup, which is likely to generate lower levels of oxidative stress. Additionally, these differences in cellular responses, together with the greater number of differentially-regulated transcripts in the LG group compared to the LR group, also suggests that glyphosate is likely more toxic to brown trout than Roundup formulation containing equivalent concentrations of glyphosate. This contrasts with a number of previous reports which reported that formulated glyphosate herbicides are more toxic than glyphosate alone, but agrees with evidence suggesting that pure glyphosate had greater reproductive toxicity than Roundup in breeding zebrafish [28].

#### Cell proliferation and turnover

Transcript expression analysis revealed some evidence of up-regulation of cell proliferation in response to Roundup and glyphosate treatment. In particular, up-regulator of cell proliferation (*urgcp*) expression was increased in LG, while early growth response 1 (*erg1*) and connective tissue growth factor (*ctgfa*) were increased in MR and LG. In addition, syndecan (*sdc4*), a G-protein cell surface co-receptor that interacts with various growth factors was up-regulated by up to 10-fold in the LR, MR, HR and LG treatments. The insulin signalling pathway, important in the promotion of cellular growth, as well as lipid and energy metabolism and homeostasis, was also over-represented. Within these pathways, insulin-induced gene 1 (*insig1*) and insulin receptor substrates (*IRS2, IRS4*) were up-regulated in the MR and HR treatments respectively, while insulin-like growth factor binding protein (*igfbp1a*) was up-regulated by MR and LG. Various transcripts associated with the cytoskeleton were also differentially regulated, including several involved in regulating actin filament dynamics and reorganisation. These included *nav2* (down-regulated in MR group), *synpo2* (up-regulated in MR), *syncrip* (up-regulated in MR and LG), *ssh2b* (up-regulated in LR, MR, HR and LG), *fnbp* (down-regulated in MR and HR), *wasb* (down-regulated in HG) and *antxr1* (up-regulated in MR and MG). Intermediate filament related transcript (*evpla*) and microtubule associated transcript (*mapre1b*) were also up-regulated in MR and HR, and MR, respectively.

GO terms related to the extracellular matrix (ECM) were amongst the most significantly enriched, especially in the MR group. Changes in the regulation of ECM components, which have important roles in cellular proliferation and growth, cell signalling and regulation of the cell cycle, may be associated with an increased rate of cell turnover. In particular, a number of transcripts encoding collagens (*col1a1a, col1a1b, col1a2, col6a2, col6a3, col8a1a, col16a1*) were down-regulated by MR, while *col13a1* was up-regulated by HR and collagen binding protein (*col4a3bp*) was up-regulated by MR. A number of transcripts encoding matrix metalloproteinase collagenases (*mmp9, mmp13a, mmp13*) were also up-regulated across LR, MR, HR and LG treatments by 3–5 fold. Additionally, collagen metabolism is a specific target of oxidative stress; ROS are known to both inhibit collagen transcription and increase collagenase activity [54] and collagenases are suspected to contain antioxidant-response elements (AREs) [49]. In addition to collagen degradation, collagenases are also important in the cleavage and activation of molecules involved in regulation of apoptosis and immune response [54]. Other differentially regulated, interacting, ECM components include laminins (*lama4, lamb1b*; down-regulated by MR), integrin (*itgax;* up-regulated by LR, MR and HR) and fibronectin (*fndc3a*; up-regulated by LR).

A balance between programmed cell death and cell proliferation is essential for the maintenance of tissue homeostasis, and both processes are regulated by integrated, complex, signalling pathways with a considerable degree of crosstalk. It is also known that an increased rate of cell proliferation can accompany increased apoptosis to maintain tissue homeostasis [50,51]. Therefore, we hypothesise that this evidence of an up-regulation of cellular growth and proliferation, together with over-representation of terms related to the cytoskeleton and the ECM, may reflect a compensatory response to increased cellular loss through apoptosis.

#### Metabolic processes

Several transcripts encoding riboflavin transporters (*slc52a3* and *rft2*) were the most up-regulated transcripts, ranging from 5–30 fold increases across all five treatment groups. Riboflavin (vitamin B2) is the central component of the flavoproteins, flavin adenine dinucleotide (FAD) and flavin mononucleotide (FMN) [55]. Four transcripts encoding riboflavin kinase (*rfk*), a key enzyme in FMN and FAD synthesis, were additionally all up-regulated by a minimum of 2.5 fold by LR, MR, HR and LG. These strong and consistent changes in transcript expression suggest that increased synthesis of flavoproteins was a key response in brown trout exposed to Roundup and glyphosate. The redox activity of the flavin group makes FAD and FMN essential components of many cellular enzymes, including those with essential roles in cellular respiration such as succinate dehydrogenase (FAD) and complex I NADH ubiquitinone dehydrogenase (FMN). These results may therefore suggest an up-regulation of aerobic respiration, perhaps to meet the energetic demands of increased cellular turnover and other aspects of cellular stress response to glyphosate and Roundup toxicity. This corresponds with existing evidence of an up-regulation of transcripts involved in energy metabolism following exposure to environmentally relevant concentrations of glyphosate in flounder [56] and oyster [57], although very high concentrations of > 84.5 mg/L Roundup were found to impair respiration in isolated rat liver cells [58]. The energetic demand associated with the cellular stress response to glyphosate and Roundup exposure could be expected to have wider health impacts. We found no evidence of an impact on the size or condition of individuals in this short term exposure under experimental conditions, but the possibility of adverse impacts on the growth and survival of fish following chronic exposure in the wild, where many factors including food availability and water quality may fluctuate and limit growth and survival, cannot be excluded.

There was an enrichment of GO terms related to a number of other metabolic processes, especially lipid metabolism in the LR and MR treatment groups (Additional file 1: Table S2). Given the role of lipids as key structural components of cellular membranes, and also in signalling and intracellular transport processes, we hypothesise that a differential regulation of lipid metabolism was induced in association with an increased rate of cell proliferation and turnover, and/or to replace lipids damaged by oxidative stress. This corresponds with previous reports of altered expression of transcripts with roles in lipid metabolism in flounder exposed to environmentally relevant concentrations of glyphosate [56,59]. In particular, we found that transcripts encoding the central transcription factor responsible for regulating cholesterol biosynthesis, *srebf2*, were amongst the most consistently up-regulated across the LR, MR, HR and LG groups. Consistent with this, transcripts encoding SREBF2-regulated cholesterol biosynthesis enzymes (*ch25h* and *sc5d*) were also up-regulated in these groups. This corresponds with previous research where treatment with both glyphosate and Roundup elevated the serum cholesterol, and triglycerides, in rats [18]. Furthermore, some evidence suggests that cholesterol may interact with ROS, and enhance antioxidant and immune response in rainbow trout [60].

A number of transcripts from the solute carrier family of membrane-bound transporters were up-regulated. *slc43*, which transports large, neutral amino acids, was amongst the most up-regulated transcripts (>10 fold) in LR, MR, HR and LG groups, and other amino acid transporters *slc3a2* (MR, HR) and *slc6a16* (LG) were also up-regulated. Additionally, citrate transporter *slc13a5* (HR, MG), monocarboxylic acid transporter *slc16a6b* (HR, LG), fatty acid transporter *slc27a4* (MR) and acetyl-coA transporter *slc33a1* (MR) were all up-regulated. Glucose and amino acid uptake via secondary transport is among the diverse functions of Na^+^/K^+^ ATPase (*atp1a2a*), which was up-regulated by 6–8 fold by LR, MR, HR and LG, while the potassium channel (*irk11*) was up-regulated in LR, MR, HR and LG. This dominant up-regulation may reflect a compensatory cellular response, associated with an increase in metabolism and turnover. Transporters of essential metals, which are essential cofactors in numerous metabolic enzymes, were also up-regulated. These included zinc (*slc39a8*) and iron (*slc25a28*) transporters which were up-regulated by MR, while the copper transporter (*slc31a1*) and iron-binding protein ferritin (*fth1b*) were up-regulated by HR.

#### Innate immune system

Functional analysis also revealed enrichment of processes involved in innate immune response. In particular, the Kegg pathways toll-like receptor (TLR) signalling, RIG-1-like (RLR) receptor signalling and Nod-like receptor (NLR) signalling were enriched. These pathways involve specific recognition of pathogen-associated molecular patterns and trigger a number of interacting downstream signalling pathways which culminate in cytokine production, recruitment of immune cells and transcriptional changes that constitute immune response [61,62]. Transcripts encoding toll-like receptor 5b (*tlr5b*), which specifically recognises bacterial flagella, were amongst the most up-regulated transcripts (between 5 and 24 fold increases) by LR, MR, HR and LG, and *tlr21* was also up-regulated in MR. In addition, transcripts linked to pathogen recognition and response through these signalling pathways were found to be up-regulated including *nod2, unc93a* and *cylda* (MR), *nlrc2* (HR) and *pglyrp6* and *rsad2* (LG). Other up-regulated transcripts included components of the complement system, *itgax* (LR, MR, HR, MG) and *c7* (MR, HR, LG) and those involved in the regulation of cytokine signalling, specifically related to interleukins (*il4r, il10r, il17r* (MR and HR), *irak3, nfil3* (MR)), interferons (*irf7, ifit5, mx2* (HR and LG)) and chemokines (*ccl5* (LG) and *ccr4* (HG)). This suggests both Roundup and glyphosate up-regulate signalling pathways involved in innate immune response. This may indicate that the immune system is a target of toxicity suggesting that these chemicals may increase susceptibility of fish to pathogen infection. This is supported by previous research which showed differential expression of immune-related transcripts following exposure to glyphosate [56,57,59] and some evidence that glyphosate formulations modulate the immune system of fish and caimen [63-65], and may alter fish susceptibility to infection [29,64].

## Conclusions

Overall, transcriptional profiling reveals that glyphosate and Roundup exposure induce alterations of many of the complex, interacting signalling pathways that control cellular stress response, in particular those involved in regulating apoptosis. Cluster analysis and examination of individual transcripts revealed there was a considerable degree of similarity between the transcript expression profiles of fish exposed to all three concentrations of Roundup and the lowest concentration of glyphosate, suggesting common mechanisms of toxicity and cellular response. These results are broadly consistent with a cellular response to oxidative stress, and we suggest that this is the most significant mechanism of toxicity of both Roundup and glyphosate. In addition, we found evidence indicating an increase in cell proliferation and cellular turnover, and an up-regulation of metabolic processes. Importantly, we found evidence of considerable transcriptional changes in fish exposed to low, environmentally-relevant, concentrations of both glyphosate and Roundup, which were broadly similar to those occurring at higher treatment concentrations. Together, our data raises concerns about the potential for environmental relevant concentrations of glyphosate and Roundup to cause adverse health effects in wild fish populations.

## Additional file

Additional file 1: Table S1. Comparative statistics for Trinity and Velvet-Oases assemblies. **Figure S1.** Multidimensional scaling plots illustrating expression profiles for all treatments. **Figure S2.** Smear plots illustrating differential expressed transcripts for each treatment. **Figure S3.** Venn diagrams illustrating overlaps between regulated transcripts in each treatment group, based on the Velvet-Oases assembly. **Figures S4 and S5.** Results of ERCC spike control analysis. **Table S2.** Enriched GO terms and Kegg pathways for each treatment group. **Table S3.** All differentially expressed transcripts in each treatment group.

## References

[1] Schonbrunn E, Eschenburg S, Shuttleworth WA, Schloss JV, Amrhein N, Evans JNS (2001). Interaction of the herbicide glyphosate with its target enzyme 5-enolpyruvylshikimate 3-phosphate synthase in atomic detail. Proc Natl Acad Sci.

[2] Steinrucken HC, Amrhein N (1980). The herbicide glyphosate is a potent inhibitor of 5-enolpyruvylshikimic acid-3-phosphate synthase. Biochem Biophys Res Commun.

[3] European Commission (2007). The use of plant protection products in the European Union- Data 1992-2003. Eurostat Statistical books.

[4] US EPA: Pesticides Industry Sales and Usage: 2006 and 2007 Market Estimates. 2011

[5] Botta F, Lavison G, Couturier G, Alliot F, Moreau-Guigon E, Fauchon N (2009). Transfer of glyphosate and its degradate AMPA to surface waters through urban sewerage systems. Chemosphere.

[6] Brausch JM, Smith PN (2007). Toxicity of three polyethoxylated tallowamine surfactant formulations to laboratory and field collected fairy shrimp, *Thamnocephalus platyurus*. Arch Environ Contam Toxicol.

[7] Giesy JP, Dobson S, Solomon KR (2000). Ecotoxicological risk assessment for Roundup® herbicide. Rev Environ Contam Toxicol.

[8] Byer JD, Struger J, Klawunn P, Todd A, Sverko E (2008). Low cost monitoring of glyphosate in surface waters using the ELISA method: An evaluation. Environ Sci Technol.

[9] Struger J, Thompson D, Staznik B, Martin P, McDaniel T, Marvin C (2008). Occurrence of glyphosate in surface waters of southern Ontario. Bull Environ Contam Toxicol.

[10] Battaglin WA, Rice KC, Focazio MJ, Salmons S, Barry RX (2009). The occurrence of glyphosate, atrazine, and other pesticides in vernal pools and adjacent streams in Washington, DC, Maryland, Iowa, and Wyoming, 2005–2006. Environ Monit Assess.

[11] MLHB: Determination of Glyphosate residues in human urine samples from 18 European countries. Report Glyphosate MLHB-2013-06-06 2013.

[12] Cavalcante D, Martinez C, Sofia S (2008). Genotoxic effects of Roundup® on the fish *Prochilodus lineatus*. Mutat Res/Genet Toxicol Environ Mutagen.

[13] Modesto KA, Martinez CB (2010). Roundup causes oxidative stress in liver and inhibits acetylcholinesterase in muscle and brain of the fish *Prochilodus lineatus*. Chemosphere.

[14] Cavas T, Konen S (2007). Detection of cytogenetic and DNA damage in peripheral erythrocytes of goldfish (*Carassius auratus*) exposed to a glyphosate formulation using the micronucleus test and the comet assay. Mutagenesis.

[15] Guilherme S, Gaivao I, Santos M, Pacheco M (2010). European eel (Anguilla anguilla) genotoxic and pro-oxidant responses following short-term exposure to Roundup®, a glyphosate-based herbicide. Mutagenesis.

[16] Guilherme S, Santos M, Barroso C, Gaivao I, Pacheco M (2012). Differential genotoxicity of Roundup® formulation and its constituents in blood cells of fish (*Anguilla anguilla*): considerations on chemical interactions and DNA damaging mechanisms. Ecotoxicol.

[17] de Castilhos Ghisi N, Cestari MM (2013). Genotoxic effects of the herbicide Roundup in the fish Corydoras paleatus (Jenyns 1842) after short-term, environmentally low concentration exposure. Environ Monit Assess.

[18] El-Shenawy NS (2009). Oxidative stress responses of rats exposed to Roundup and its active ingredient glyphosate. Environ Toxicol Pharmacol.

[19] Astiz M, Alaniz MJ, Marra CA (2009). Effect of pesticides on cell survival in liver and brain rat tissues. Ecotoxicol Environ Saf.

[20] Gasnier C, Dumont C, Benachour N, Clair E, Chagnon MC, Seralini GE (2009). Glyphosate-based herbicides are toxic and endocrine disruptors in human cell lines. Toxicology.

[21] Clair A, Mesnage R, Travert C, Seralini GE (2012). A glyphosate-based herbicide induces necrosis and apoptosis in mature rat testicular cells in vitro, and testosterone decrease at lower levels. Toxicol in Vitro.

[22] Y-h K, Hong J-R, Gil H-W, Song H-Y, Hong S-Y (2013). Mixtures of glyphosate and surfactant TN20 accelerate cell death via mitochondrial damage-induced apoptosis and necrosis. Toxicol in Vitro.

[23] Mesnage R, Bernay B, Seralini G-E (2013). Ethoxylated adjuvants of glyphosate-based herbicides are active principles of human cell toxicity. Toxicology.

[24] Benachour N, Seralini G-E (2008). Glyphosate formulations induce apoptosis and necrosis in human umbilical, embryonic, and placental cells. Chem Res Toxicol.

[25] Walsh LP, McCormick C, Martin C, Stocco DM (2000). Roundup inhibits steroidogenesis by disrupting steroidogenic acute regulatory (StAR) protein expression. Environ Health Perspect.

[26] Romano MA, Romano RM, Santos LD, Wisniewski P, Campos DA, de Souza PB (2012). Glyphosate impairs male offspring reproductive development by disrupting gonadotropin expression. Arch Toxicol.

[27] Dallegrave E, Mantese FD, Oliveira RT, Andrade AJ, Dalsenter PR, Langeloh A (2007). Pre-and postnatal toxicity of the commercial glyphosate formulation in Wistar rats. Arch Toxicol.

[28] Uren Webster TM, Laing LV, Florance H, Santos EM (2014). Effects of Glyphosate and its Formulation, Roundup, on Reproduction in Zebrafish (*Danio rerio*). Environ Sci Technol.

[29] Kelly DW, Poulin R, Tompkins DM, Townsend CR (2010). Synergistic effects of glyphosate formulation and parasite infection on fish malformations and survival. J Appl Ecol.

[30] Howe CM, Berrill M, Pauli BD, Helbing CC, Werry K, Veldhoen N (2004). Toxicity of glyphosate based pesticides to four North American frog species. Environ Toxicol Chem.

[31] Relyea RA (2012). New effects of roundup on amphibians: predators reduce herbicide mortality; herbicides induce antipredator morphology. Ecol Appl.

[32] Paull GC, Van Look KJW, Santos EM, Filby AL, Gray DM, Nash JP (2008). Variability in measures of reproductive success in laboratory-kept colonies of zebrafish and implications for studies addressing population-level effects of environmental chemicals. Aquat Toxicol.

[33] Hansen KD, Brenner SE, Dudoit S (2010). Biases in Illumina transcriptome sequencing caused by random hexamer priming. Nucleic Acids Res.

[34] Brown CT, Howe A, Zhang Q, Pyrkosz AB, Brom TH: A reference-free algorithm for computational normalization of shotgun sequencing data. arXiv:12034802 [q-bioGN] 2012.

[35] Francis WR, Christianson LM, Kiko R, Powers ML, Shaner NC, Haddock SH (2013). A comparison across non-model animals suggests an optimal sequencing depth for de novo transcriptome assembly. BMC Genomics.

[36] Zerbino DR, Birney E (2008). Velvet: Algorithms for de novo short read assembly using de Bruijn graphs. Genome Res.

[37] Schulz MH, Zerbino DR, Vingron M, Birney E (2012). Oases: Robust de novo RNA-seq assembly across the dynamic range of expression levels. Bioinformatics.

[38] Grabherr MG, Haas BJ, Yassour M, Levin JZ, Thompson DA, Amit I (2011). Full-length transcriptome assembly from RNA-Seq data without a reference genome. Nat Biotechnol.

[39] Langmead B, Salzberg SL (2012). Fast gapped-read alignment with Bowtie 2. Nat Methods.

[40] Li H, Handsaker B, Wysoker A, Fennell T, Ruan J, Homer N (2009). The sequence alignment/map format and SAMtools. Bioinformatics.

[41] Robinson MD, McCarthy DJ, Smyth GK (2010). edgeR: a Bioconductor package for differential expression analysis of digital gene expression data. Bioinformatics.

[42] Huang DW, Sherman BT, Lempicki RA (2008). Systematic and integrative analysis of large gene lists using DAVID bioinformatics resources. Nat Protoc.

[43] Lu B, Zeng Z, Shi T (2013). Comparative study of de novo assembly and genome-guided assembly strategies for transcriptome reconstruction based on RNA-Seq. Sci China Life Sci.

[44] Vandenberg LN, Colborn T, Hayes TB, Heindel JJ, Jacobs DR, Lee D-H (2012). Hormones and endocrine-disrupting chemicals: low-dose effects and nonmonotonic dose responses. Endocr Rev.

[45] Conolly RB, Lutz WK (2004). Nonmonotonic dose–response relationships: mechanistic basis, kinetic modeling, and implications for risk assessment. Toxicol Sci.

[46] Solomon KR, Dalhoff K, Volz D, Van der Kraak G, Tierney KB, Farrell AP, Brauner CJ (2013). Effects of Herbicides on Fish. Fish physiology Vol 33.

[47] Martindale JL, Holbrook NJ (2002). Cellular response to oxidative stress: Signaling for suicide and survival*. J Cell Physiol.

[48] Bauer M, Bauer I (2002). Heme oxygenase-1: redox regulation and role in the hepatic response to oxidative stress. Antioxid Redox Signal.

[49] Di Giulio RT, Meyer JN, Di Gulio RT, Hinton DE (2008). Reactive Oxygen Species and Oxidative Stress. The Toxicology of Fishes.

[50] Andreef M, Goodrich DW, Pardee AB: Cell Proliferation, Differentiation, and Apoptosis. In: Holland-Frei Cancer Medicine. Edited by Bast RC, Pollock RE, 5th edn: Hamilton (ON): BC Decker; 2000.

[51] Mates J, Segura JA, Alonso FJ, Marquez J (2008). Intracellular redox status and oxidative stress: implications for cell proliferation, apoptosis, and carcinogenesis. Arch Toxicol.

[52] Welch C, Santra MK, El-Assaad W, Zhu X, Huber WE, Keys RA (2009). Identification of a protein, G0S2, that lacks Bcl-2 homology domains and interacts with and antagonizes Bcl-2. Cancer Res.

[53] Ouyang L, Shi Z, Zhao S, Wang FT, Zhou TT, Liu B (2012). Programmed cell death pathways in cancer: a review of apoptosis, autophagy and programmed necrosis. Cell Prolif.

[54] Siwik DA, Pagano PJ, Colucci WS (2001). Oxidative stress regulates collagen synthesis and matrix metalloproteinase activity in cardiac fibroblasts. Am J Physiol Cell Physiol.

[55] Van Berkel W, Kamerbeek N, Fraaije M (2006). Flavoprotein monooxygenases, a diverse class of oxidative biocatalysts. J Biotechnol.

[56] Marchand J, Tanguy A, Charrier G, Quiniou L, Plee-Gauthier E, Laroche J (2006). Molecular identification and expression of differentially regulated genes of the European flounder, *Platichthys flesus*, submitted to pesticide exposure. Mar Biotechnol.

[57] Tanguy A, Boutet I, Laroche J, Moraga D (2005). Molecular identification and expression study of differentially regulated genes in the Pacific oyster *Crassostrea gigas* in response to pesticide exposure. FEBS J.

[58] Peixoto F (2005). Comparative effects of the Roundup and glyphosate on mitochondrial oxidative phosphorylation. Chemosphere.

[59] Evrard E, Devaux A, Bony S, Burgeot T, Riso R, Budzinski H (2010). Responses of the European flounder Platichthys flesus to the chemical stress in estuaries: Load of contaminants, gene expression, cellular impact and growth rate. Biomarkers.

[60] Deng J, Kang B, Tao L, Rong H, Zhang X (2013). Effects of dietary cholesterol on antioxidant capacity, non-specific immune response, and resistance to *Aeromonas hydrophila* in rainbow trout (*Oncorhynchus mykiss*) fed soybean meal-based diets. Fish Shellfish Immunol.

[61] Kawai T, Akira S (2008). Toll-like Receptor and RIG-1-like Receptor Signaling. Ann N Y Acad Sci.

[62] Janeway CA, Medzhitov R (2002). Innate immune recognition. Annu Rev Immunol.

[63] El-Gendy K, Aly N, El-Sebae A (1998). Effects of edifenphos and glyphosate on the immune response and protein biosynthesis of bolti fish (*Tilapia nilotica*). J Environ Sci Health Part B.

[64] Kreutz LC, Gil Barcellos LJ, Marteninghe A, Davi dos Santos E, Zanatta R (2010). Exposure to sublethal concentration of glyphosate or atrazine-based herbicides alters the phagocytic function and increases the susceptibility of silver catfish fingerlings (*Rhamdia quelen*) to *Aeromonas hydrophila* challenge. Fish Shellfish Immunol.

[65] Latorre MA, Lopez Gonzallez EC, Larriera A, Poletta GL, Siroski PA (2013). Effects of in vivo exposure to Roundup® on immune system of Caiman latirostris. J Immunotoxicol.

