# Supplementary material for: Global transcriptomic profiling demonstrates induction of oxidative stress and of compensatory cellular stress responses in brown trout exposed to glyphosate and Roundup
Source: BMC Genomics. 2015 Jan 31;16(1):32. doi: 10.1186/s12864-015-1254-5 (PMC4318436; doi:10.1186/s12864-015-1254-5)
Supplement: Additional file 1: Table S1. — Comparative statistics for Trinity and Velvet-Oases assemblies. Figure S1. Multidimensional scaling plots illustrating expression profiles for all treatments. Figure S2. Smear plots illustrating differential expressed transcripts for each treatment. Figure S3. Venn diagrams illustrating overlaps between regulated transcripts in each treatment group, based on the Velvet-Oases assembly. Figures S4 and S5. Results of ERCC spike control analysis. Table S2. Enriched GO terms and Kegg pathways for each treatment group. Table S3. All differentially expressed transcripts in each treatment group. [file 12864_2015_1254_MOESM1_ESM.docx]

**Additional file 1**

**Global transcriptomic profiling demonstrates induction of oxidative stress and of compensatory cellular stress responses in brown trout exposed to glyphosate and Roundup.**

Tamsyn. M. Uren Webster^1*^ and Eduarda M. Santos^1^*

1. Biosciences, College of Life & Environmental Sciences, Geoffrey Pope Building, University of Exeter, Exeter, EX4 4QD

* Corresponding authors

This Supporting Information contains:

Page S2: Comparative statistics for Trinity and Velvet-Oases assemblies, **Table S1**
Page S3: Multidimensional scaling plots illustrating expression profiles for all treatments, **Figure S1**

Page S4: Smear plots illustrating differential expressed transcripts for each treatment, **Figure S2**

Page S5: Venn diagrams illustrating overlaps between regulated transcripts in each treatment group, based on the Velvet-Oases assembly, **Figure S3**

Page S6-S8: Results of ERCC spike control analysis, **Figure S4, Figure S5**

Page S9-10: Enriched GO terms and Kegg pathways for each treatment group, **Table S2**

Page S11-S25: All differentially expressed transcripts, **Table S3**

Page S25: Reference list

| **Assembly** | **No. transcripts** | **No. Loci** | **Mean length (bp)** | **N50 (bp)** | **% annotated** | **No. zf transcript annotations** | **% reads**  **re-mapped** | **No. transcripts retained by EdgeR** | **No. retained zf transcript annotations** | **Mean BCV** |
| --- | --- | --- | --- | --- | --- | --- | --- | --- | --- | --- |
| Velvet-Oases | 893,904 | 146,233 | 1198.4 | 2012 | 47 % | 19,893 | 94 % | 115,217 | 8,966 | 37.4 % |
| Trinity | 258,702 | 109,301 | 1065.6 | 2107 | 45 % | 17,852 | 89 % | 67,954 | 11,886 | 34.6 % |

**Table S1.** Comparative summary statistics describing the *de novo* transcriptome assemblies constructed using the Velvet-Oases and Trinity pipelines. Data presented include the number and length of transcripts built for each assembly and the percentage of transcripts annotated using Blastx against Ensembl peptide databases using an e-value cut off < 1e^-15^; the percentage of raw reads that re-mapped against each assembly using Bowtie2; the number of transcripts that passed the imposed criteria for inclusion in EdgeR analysis (at least 1 mapped read in at least 6 replicate libraries) and the number of unique zebrafish transcript annotations included within these; and the mean biological coefficient of variation (BCV) calculated using EdgeR for each treatment group.


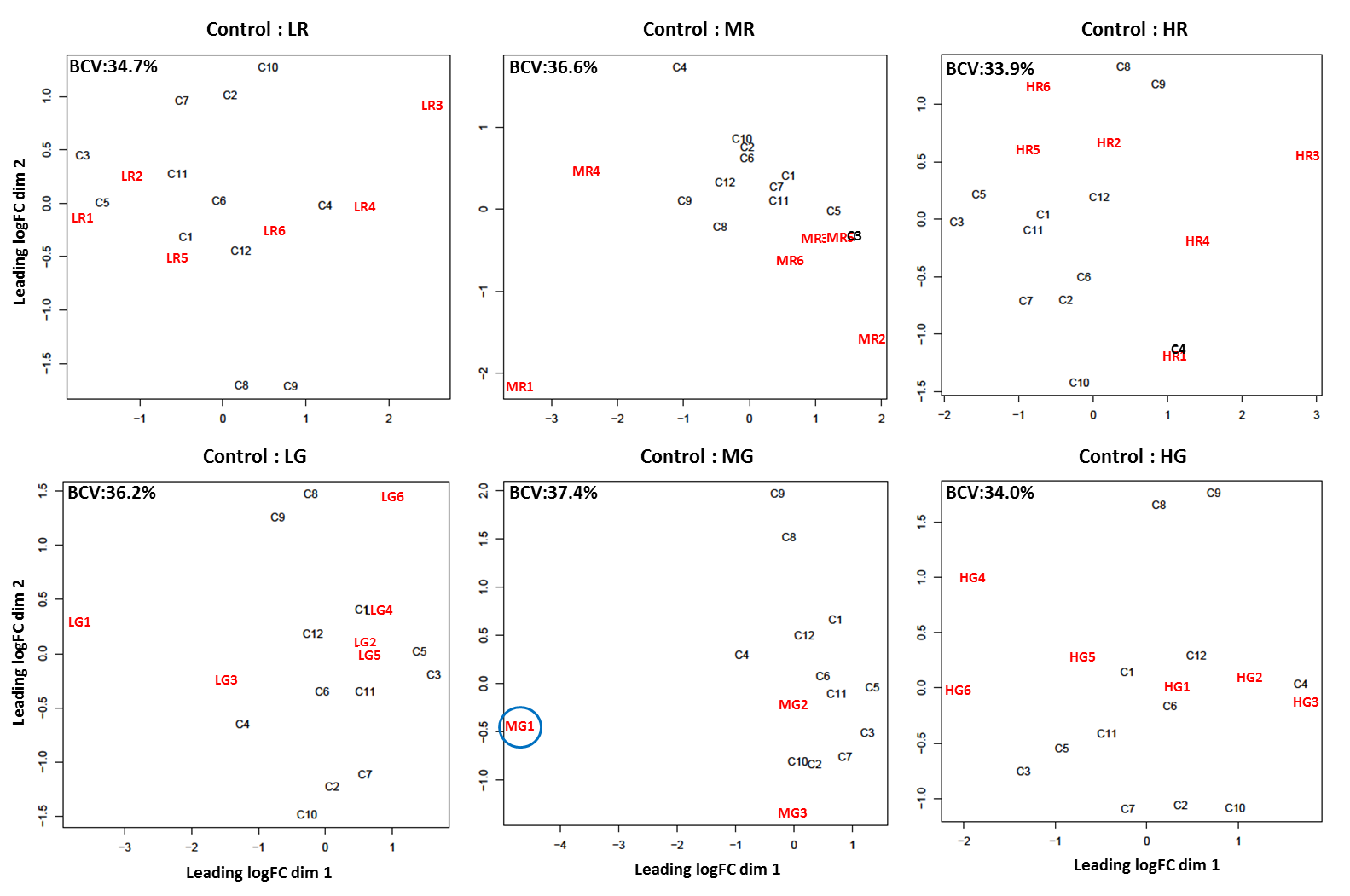


**Figure S1.** Multidimensional scaling plots illustrating the similarity of expression profiles for individual replicates in each treatment group compared to the control group, based on the expression of all transcripts in each pairwise comparison. Treatments are represented by the following codes: LR, MR and HR represent 0.01, 0.5 and 10 mg Roundup/L, and LG, MG and HG represent 0.01, 0.5 and 10 mg glyphosate /L. BCV: Biological coefficient of variation for the pairwise test between a given treatment group and the control groups.


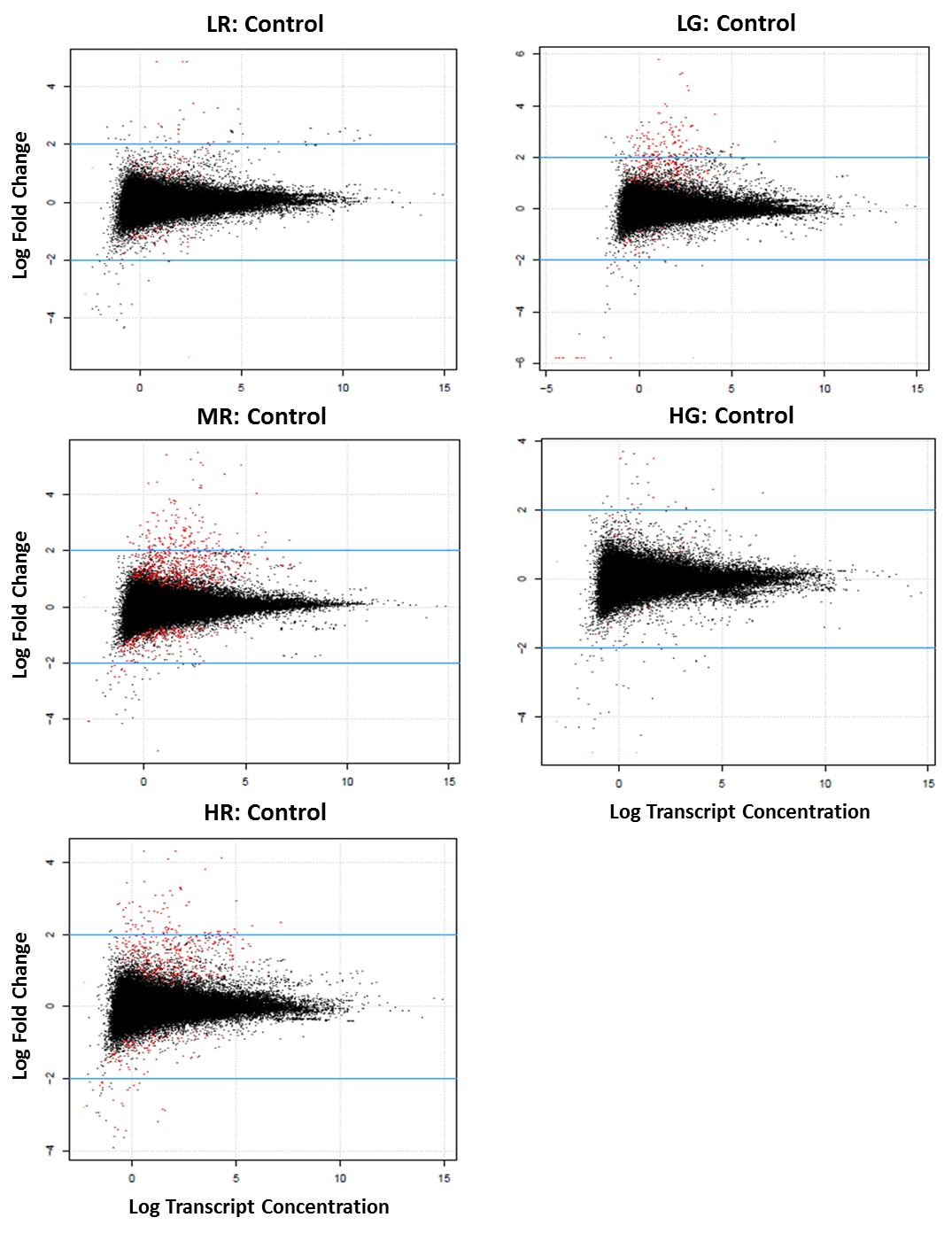


**Figure S2.** Smear plots illustrating differential expressed transcripts for each treatment group. Values plotted represent the concentration and fold change compared to the control group for all transcripts included in each pairwise test. Red dots represent differentially expressed transcripts. Treatments are represented by the following codes: LR, MR and HR represent 0.01, 0.5 and 10 mg Roundup/L and LG and HG represent 0.01 and 10 mg glyphosate/L.


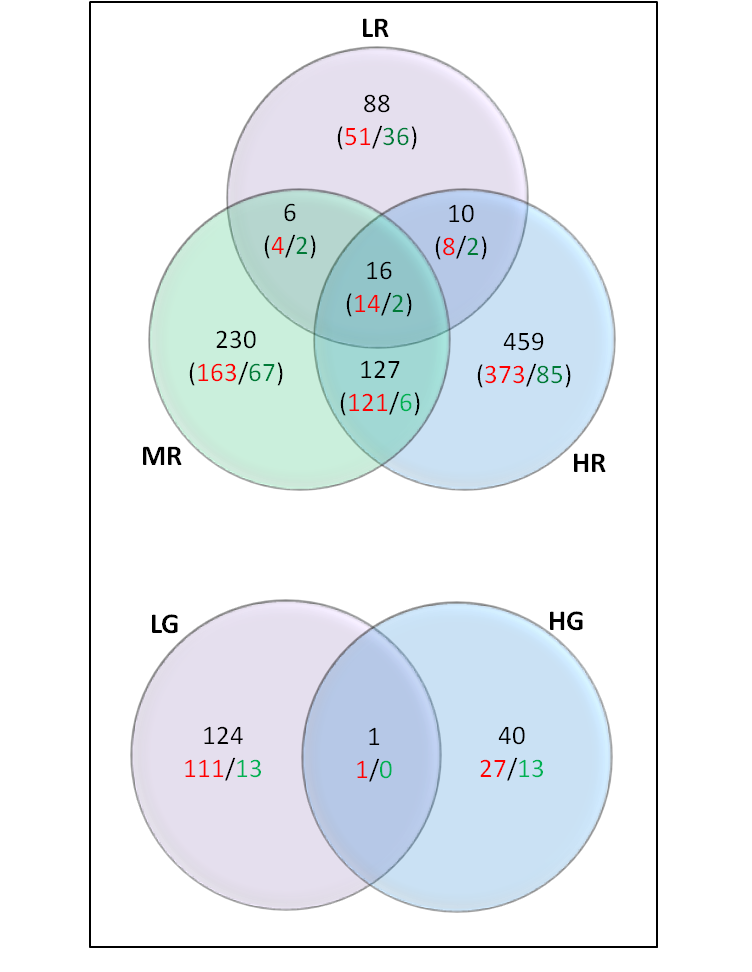


**Figure S3.** Venn diagrams illustrating the numbers of differentially expressed transcripts (FDR<0.1) and overlaps between differentially expressed transcripts in each treatment group, obtained from EdgeR, based on the Velvet-Oases assembly. Red and green numbers represent up- and down-regulated transcripts, respectively. Treatments are represented by the following codes: LR, MR and HR represent 0.01, 0.5 and 10 mg Roundup/L and LG and HG represent 0.01 and 10 mg glyphosate/L.

**
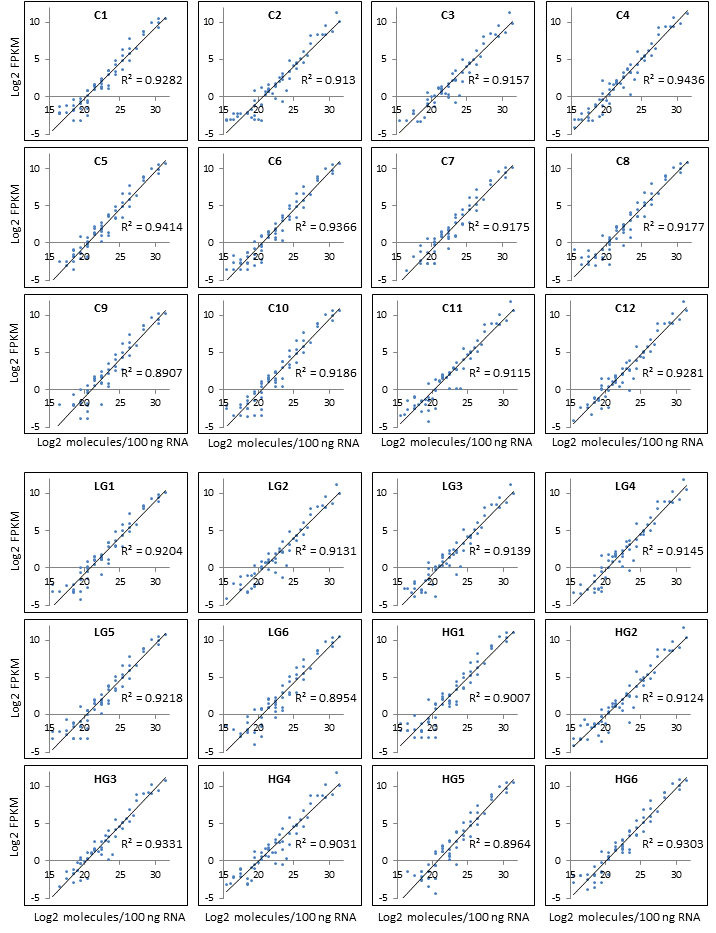
**

**
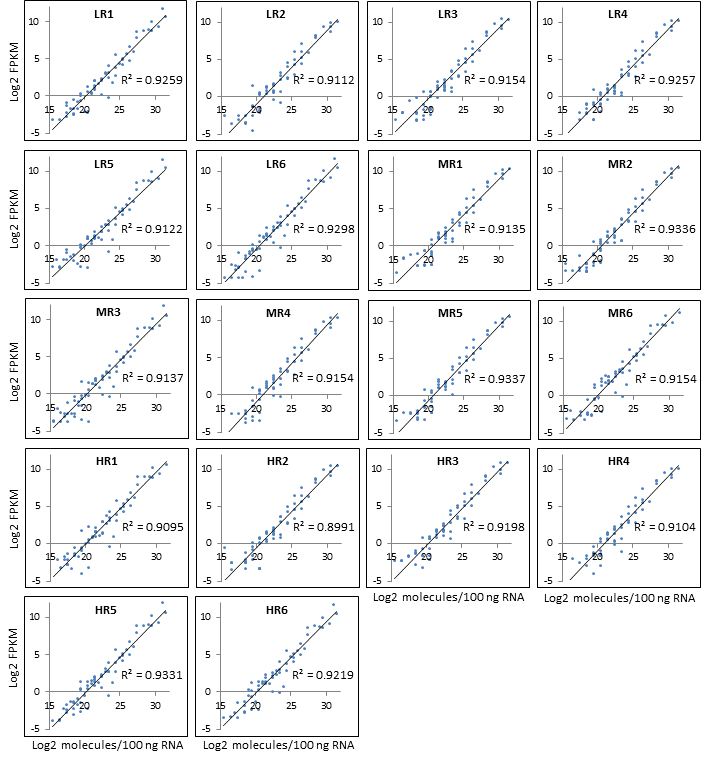
**

**Figure S4.** External RNA Controls Consortium **(**ERCC) spike-in control analysis for all individual liver samples sequenced in this project. Graphs show the relationship between the calculated expression level (FPKM) and the expected concentration of each control transcripts. Individual fish are represented by the following codes: C1-C12 represent the control individuals; LR1-LR6 represent individuals exposed to 0.01 mg Roundup/L; MR1-MR6 represent individuals exposed to 0.5 mg Roundup/L; HR1-HR6 represent individuals exposed to 10 mg Roundup/L; LG1-LG6 represent individuals exposed to 0.01 mg glyphosate/L; and HG1-HG6 represent individuals exposed to 10 mg glyphosate/L.

**
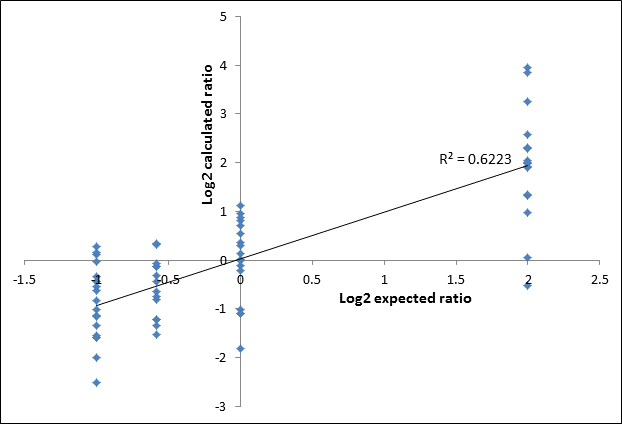
**

**Figure S5.** The relationship between the expected and calculated fold change in expression of ERCC spike-in control transcripts. Values plotted are log2 transformed ratios of transcript expression (FPKM) in samples spiked with ERCC mix 1 and mix 2.

**Table S2.** Gene Ontology Terms and Kegg Pathways over-represented in the list of differentially expressed transcripts for each treatment group. Values presented are the P-values and adjusted P-values associated with this over-representation. Analysis was conducted using the Database for Annotation, Visualization and Integrated Discovery (DAVID ) v6 .7 (Huang et al. 2008) using the *de novo* liver transcriptome assembly generated using Trinity in this study as a background.

| **BIOLOGICAL PROCESS (BP ALL)** | **0.01 mg Roundup/ L** | | **0.5 mg Roundup/ L** | | **10 mg Roundup/ L** | | **0.01 mg glyphosate/ L** | | | **10 mg glyphosate/ L** | |
| --- | --- | --- | --- | --- | --- | --- | --- | --- | --- | --- | --- |
|  | **P Value** | **FDR** | **P Value** | **FDR** | **P Value** | **FDR** | **P Value** | | **FDR** | **P Value** | **FDR** |
| biogenic amine metabolic process |  |  | 4.80E-02 | 5.50E+01 |  |  |  | |  |  |  |
| biological regulation |  |  | 1.30E-02 | 1.80E+01 |  |  |  | |  |  |  |
| cellular amino acid derivative metabolic process |  |  | 2.40E-02 | 3.00E+01 |  |  |  | |  |  |  |
| defense response |  |  |  |  |  |  | 4.50E-02 | | 5.50E+01 |  |  |
| diencephalon development | 4.30E-02 | 4.30E+01 |  |  |  |  |  | |  |  |  |
| lipid metabolic process |  |  | 4.10E-02 | 4.70E+01 |  |  |  | |  |  |  |
| mesoderm development |  |  |  |  | 2.00E-02 | 2.30E+01 |  | |  |  |  |
| multi-organism process |  |  |  |  |  |  | 3.30E-05 | | 4.70E-02 |  |  |
| positive regulation of apoptosis |  |  |  |  |  |  | 4.70E-02 | | 5.70E+01 |  |  |
| positive regulation of cell death |  |  |  |  |  |  | 4.70E-02 | | 5.70E+01 |  |  |
| positive regulation of programmed cell death |  |  |  |  |  |  | 4.70E-02 | | 5.70E+01 |  |  |
| regulation of biological process | 5.00E-02 | 4.90E+01 | 3.70E-03 | 5.40E+00 |  |  |  | |  |  |  |
| regulation of biosynthetic process | 7.90E-03 | 9.60E+00 | 3.70E-03 | 5.30E+00 |  |  |  | |  |  |  |
| regulation of cell growth |  |  |  |  |  |  | 4.40E-02 | | 4.90E+01 |  |  |
| regulation of cellular biosynthetic process | 7.80E-03 | 9.50E+00 | 3.50E-03 | 5.10E+00 |  |  |  | |  |  |  |
| regulation of cellular metabolic process | 1.10E-02 | 1.40E+01 | 8.50E-03 | 1.20E+01 |  |  |  | |  |  |  |
| regulation of cellular process | 3.70E-02 | 3.80E+01 | 4.10E-03 | 6.00E+00 |  |  |  | |  |  |  |
| regulation of gene expression | 9.10E-03 | 1.10E+01 | 5.00E-03 | 7.20E+00 |  |  | 4.10E-02 | | 4.00E+00 |  |  |
| regulation of growth |  |  |  |  |  |  | 4.50E-02 | | 5.50E+01 |  |  |
| regulation of macromolecule biosynthetic process | 7.60E-03 | 9.20E+00 | 3.30E-03 | 4.80E+00 |  |  |  | |  |  |  |
| regulation of macromolecule metabolic process | 1.40E-02 | 1.60E+01 | 1.30E-02 | 1.70E+01 |  |  |  | |  |  |  |
| regulation of metabolic process | 1.70E-02 | 1.90E+01 | 2.00E-02 | 2.60E+01 |  |  |  | |  |  |  |
| regulation of nitrogen compound metabolic process | 2.70E-02 | 2.90E+01 | 1.10E-02 | 1.50E+01 |  |  |  | |  |  |  |
| regulation of primary metabolic process | 1.20E-02 | 1.40E+01 | 9.80E-03 | 1.40E+01 |  |  |  | |  |  |  |
| regulation of RNA metabolic process |  |  | 2.30E-03 | 3.40E+00 |  |  |  | |  |  |  |
| regulation of transcription | 2.30E-02 | 2.50E+01 | 7.40E-03 | 1.00E+01 |  |  |  | |  |  |  |
| regulation of transcription, DNA-dependent |  |  | 1.90E-03 | 1.00E+01 |  |  |  | |  |  |  |
| response to bacterium |  |  |  |  |  |  | 3.40E-02 | | 3.80E+01 |  |  |
| response to biotic stimulus |  |  |  |  |  |  | 3.80E-06 | | 5.40E-03 |  |  |
| response to other organism |  |  |  |  |  |  | 1.80E-05 | | 2.50E-02 |  |  |
| response to stimulus |  |  | 2.30E-02 | 2.90E+01 | 2.30E-02 | 2.70E+01 | 4.60E-04 | | 6.40E-01 |  |  |
| response to stress |  |  |  |  | 4.80E-02 | 5.50E+01 | 5.20E-03 | | 7.00E+00 |  |  |
| response to virus |  |  |  |  |  |  | 2.20E-03 | | 3.00E+00 |  |  |
| sterol metabolic process |  |  | 4.30E-02 | 4.80E+01 |  |  |  | |  |  |  |
| **MOLECULAR FUNCTION (MF ALL)** | **0.01 mg Roundup/ L** | | **0.5 mg Roundup/ L** | | **10 mg Roundup/ L** | | **0.01 mg glyphosate/ L** | | | **10 mg glyphosate/ L** | |
|  | **P Value** | **FDR** | **P Value** | **FDR** | **P Value** | **FDR** | | **P Value** | **FDR** | **P Value** | **FDR** |
| adenyl ribonucleotide binding |  |  |  |  |  |  | | 4.90E-02 | 4.00E+01 |  |  |
| calcium-transporting ATPase activity |  |  |  |  |  |  | | 5.00E-02 | 5.80E+01 |  |  |
| carboxy-lyase activity |  |  | 4.10E-02 | 4.80E+01 |  |  | |  |  |  |  |
| DNA binding |  |  | 9.60E-03 | 1.20E+01 | 2.50E-02 | 2.50E+01 | |  |  |  |  |
| extracellular matrix structural constituent |  |  | 2.60E-02 | 2.80E+01 |  |  | |  |  |  |  |
| heat shock protein binding |  |  |  |  |  |  | | 4.30E-02 | 4.10E+01 |  |  |
| ligand-dependent nuclear receptor activity |  |  | 4.60E-02 | 5.20E+01 |  |  | |  |  |  |  |
| metalloendopeptidase activity |  |  |  |  | 2.90E-02 | 2.90E+01 | |  |  |  |  |
| nucleotide binding |  |  | 3.50E-02 | 3.60E+01 |  |  | | 4.30E-02 | 4.10E+01 |  |  |
| purine nucleotide binding |  |  |  |  |  |  | | 3.00E-02 | 4.00E+01 |  |  |
| purine ribonucleotide binding |  |  |  |  |  |  | | 4.30E-02 | 4.10E+01 |  |  |
| SAP kinase activity |  |  | 4.40E-02 | 5.00E+01 |  |  | |  |  |  |  |
| sequence-specific DNA binding |  |  | 3.40E-02 | 3.60E+01 |  |  | |  |  |  |  |
| steroid hormone receptor activity |  |  | 4.60E-02 | 5.20E+01 |  |  | |  |  |  |  |
| transcription factor activity |  |  | 1.10E-03 | 1.40E+00 | 3.90E-02 | 5.70E+01 | | 4.90E-02 | 6.30E+01 |  |  |
| transcription regulator activity | 5.90E-03 | 5.80E+00 | 1.60E-03 | 2.00E+00 |  |  | | 4.60E-02 | 6.10E+01 |  |  |
| transcription repressor activity |  |  | 4.50E-02 | 5.10E+01 |  |  | |  |  |  |  |
| **CELLULAR COMPONENT (CC ALL)** | **0.01 mg Roundup/ L** | | **0.5 mg Roundup/ L** | | **10 mg Roundup/ L** | | **0.01 mg glyphosate/ L** | | | **10 mg glyphosate/ L** | |
|  | **P Value** | **FDR** | **P Value** | **FDR** | **P Value** | **FDR** | **P Value** | | **FDR** | **P Value** | **FDR** |
| nucleus | 8.40E-03 | 7.50E+00 |  |  | 4.80E-02 | 5.60E+01 |  | |  |  |  |
| collagen |  |  | 1.50E-02 | 1.60E+01 |  |  |  | |  |  |  |
| endoplasmic reticulum |  |  | 1.40E-02 | 1.40E+01 |  |  | 7.30E-02 | | 5.30E+01 |  |  |
| external side of plasma membrane |  |  |  |  |  |  | 6.40E-02 | | 4.80E+01 |  |  |
| extracellular matrix |  |  | 7.40E-04 | 8.10E-01 |  |  |  | |  |  |  |
| extracellular region |  |  | 5.60E-03 | 6.00E+00 |  |  | 4.70E-02 | | 3.80E+01 |  |  |
| proteinaceous extracellular matrix |  |  | 6.00E-04 | 6.50E-01 |  |  |  | |  |  |  |
| **KEGG PATHWAY** | **0.01 mg Roundup/ L** | | **0.5 mg Roundup/ L** | | **10 mg Roundup/ L** | | **0.01 mg glyphosate/ L** | | | **10 mg glyphosate/ L** | |
|  | **P Value** | **FDR** | **P Value** | **FDR** | **P Value** | **FDR** | **P Value** | | **FDR** | **P Value** | **FDR** |
| Adipocytokine signaling pathway |  |  |  |  | 4.90E-02 | 3.50E+01 |  | |  |  |  |
| Apoptosis |  |  | 4.80E-02 | 6.10E+01 |  |  |  | |  |  |  |
| ECM-receptor interaction |  |  | 7.90E-03 | 7.80E+00 |  |  |  | |  |  |  |
| Glycerophospholipid metabolism |  |  | 3.60E-02 | 3.20E+01 |  |  |  | |  |  |  |
| Insulin signaling pathway |  |  |  |  | 5.90E-03 | 4.90E+00 | 4.10E-02 | | 5.30E+01 |  |  |
| MAPK signaling pathway |  |  | 4.80E-02 | 5.70E+01 |  |  | 7.20E-03 | | 6.20E+00 |  |  |
| NOD-like receptor signaling pathway |  |  | 4.80E-02 | 6.10E+01 |  |  |  | |  |  |  |
| RIG-I-like receptor signaling pathway |  |  | 3.70E-03 | 3.80E+00 |  |  |  | |  |  |  |
| Sphingolipid metabolism |  |  | 8.90E-03 | 8.80E+00 |  |  |  | |  |  |  |
| TGF-beta signaling pathway | 2.80E-03 | 2.10E+00 |  |  |  |  |  | |  |  |  |
| Toll-like receptor signaling pathway |  |  | 5.00E-03 | 5.10E+00 |  |  |  | |  |  |  |

**Table S3.** Fold changes of all differentially-regulated transcripts in all treatment groups.
Values presented are log2 transformed fold changes calculated by EdgeR. Significant differences in expression (FDR <0.1) are indicated by red (up-regulated) and green (down-regulated) shading. Treatments are represented by the following codes: LR, MR and HR represent 0.01, 0.5 and 10 mg/L Roundup, and LG and HG represent 0.01 and 10 mg/L glyphosate.

|  | | | **LR** | **MR** | **HR** | **LG** | **HG** |
| --- | --- | --- | --- | --- | --- | --- | --- |
| **Name** | **Symbol** | **Database** | **Log2 FC** | **Log2 FC** | **Log2 FC** | **Log2 FC** | **Log2 FC** |
| aanat2 | NM_001124257.1 | refseq | -1.32 | -0.74 | -0.85 | -0.17 | -0.32 |
| abcf2a | ENSDARG00000038785 | Ensembl | 1.23 | 2.09 | 1.15 | 1.33 | 0.57 |
| abcf2a | ENSDARG00000038785 | Ensembl | 0.52 | 0.97 | 0.34 | 0.55 | 0.17 |
| abcf2a | ENSDARG00000038785 | Ensembl | 0.75 | 1.27 | 0.58 | 0.89 | 0.35 |
| ABCF3 | ENSDARG00000089705 | Ensembl | 1.30 | 2.21 | 1.18 | 2.00 | 0.50 |
| acsbg2 | ENSDARG00000004094 | Ensembl | -1.48 | -0.79 | -0.56 | -1.06 | -0.16 |
| acsbg2 | ENSDARG00000004094 | Ensembl | -1.41 | -0.90 | -0.80 | -0.97 | -0.21 |
| acsbg2 | ENSDARG00000004094 | Ensembl | -1.36 | -1.06 | -0.56 | -0.90 | 0.18 |
| acss1 | ENSDARG00000044142 | Ensembl | 0.68 | 1.38 | 1.59 | 2.29 | 0.02 |
| adamts10 | ENSDARG00000075188 | Ensembl | -0.57 | -0.66 | -0.33 | -0.82 | -0.17 |
| AIFM2 | ENSDARG00000077549 | Ensembl | 0.66 | 0.70 | 0.33 | 0.55 | 0.33 |
| alpk2 | ENSDARG00000079637 | Ensembl | -0.78 | -1.11 | -0.91 | -0.21 | -0.20 |
| amd1 | ENSDARG00000043856 | Ensembl | 0.49 | 0.81 | 0.57 | 0.35 | 0.21 |
| angptl4 | ENSDARG00000035859 | Ensembl | 0.66 | 0.69 | 0.13 | 0.17 | 0.08 |
| angptl4 | ENSDARG00000035859 | Ensembl | 0.69 | 0.54 | 0.30 | 0.24 | 0.19 |
| ankrd16 | ENSDARG00000003822 | Ensembl | 1.13 | 2.14 | 1.42 | 0.65 | 0.00 |
| ANKRD28 | ENSGACG00000006364 | Ensembl | 0.44 | 1.06 | 1.22 | 0.74 | 0.30 |
| ANKRD30B | ENSDARG00000013015 | Ensembl | -0.52 | -0.31 | -1.26 | -0.21 | -1.00 |
| ANTXR1_(1_of_2) | ENSDARG00000025672 | Ensembl | 0.29 | 1.07 | 0.91 | 0.14 | 0.71 |
| anxa11a | ENSDARG00000077383 | Ensembl | 0.43 | 1.21 | 0.63 | 0.64 | 0.26 |
| anxa11a | ENSDARG00000077383 | Ensembl | 0.33 | 1.33 | 0.60 | 0.68 | 0.23 |
| anxa11a | ENSDARG00000077383 | Ensembl | 0.37 | 1.14 | 0.23 | 0.54 | -0.09 |
| apooa | ENSDARG00000046154 | Ensembl | 0.18 | 0.98 | 0.22 | 0.13 | 0.30 |
| arf1l | ENSDARG00000016393 | Ensembl | 0.61 | 1.01 | 0.62 | 1.21 | 0.49 |
| arhgap21b | ENSDARG00000075673 | Ensembl | 1.04 | 1.39 | 0.59 | 0.97 | 0.73 |
| arhgap21b | ENSDARG00000075673 | Ensembl | 0.14 | 0.84 | 0.27 | 0.22 | 0.15 |
| ARHGAP32 | ENSDARG00000074184 | Ensembl | -0.35 | -1.06 | -0.40 | -0.38 | -0.52 |
| arhgap5 | ENSDARG00000061294 | Ensembl | 0.13 | 0.86 | 0.22 | 0.49 | -0.14 |
| arhgef19 | ENSDARG00000078853 | Ensembl | -0.92 | -0.50 | -1.23 | -0.73 | -0.51 |
| aste1 | ENSONIG00000002037 | Ensembl | 0.00 | 0.00 | 1.30 | 3.02 | 0.00 |
| atf3 | ENSDARG00000007823 | Ensembl | 1.33 | 3.02 | 2.12 | 2.02 | 0.83 |
| atf5b | ENSDARG00000077785 | Ensembl | 0.67 | 1.17 | 1.17 | 1.26 | 0.32 |
| atg5 | NM_001173812.1 | refseq | 1.90 | 3.74 | 2.38 | 5.26 | 0.57 |
| atg5 | NM_001173812.1 | refseq | 1.37 | 3.78 | 2.35 | 5.21 | 0.41 |
| atp1a2a | NM_001124458.1 | refseq | 2.71 | 3.07 | 3.09 | 2.89 | 1.93 |
| atp2a2a | ENSDARG00000029439 | Ensembl | 0.79 | 1.50 | 1.21 | 1.85 | 0.08 |
| atp2a2b | ENSDARG00000005122 | Ensembl | 0.96 | 1.34 | 0.95 | 2.00 | 0.01 |
| atp2a2b | ENSDARG00000005122 | Ensembl | 0.92 | 1.11 | 1.11 | 1.69 | 0.35 |
| atp2a2b | ENSDARG00000005122 | Ensembl | 0.72 | 1.07 | 0.80 | 1.41 | 0.23 |
| atp2a2b | ENSDARG00000005122 | Ensembl | 0.56 | 1.37 | 0.62 | 1.47 | 0.33 |
| bcap29 | ENSDARG00000016231 | Ensembl | 0.62 | 1.80 | 1.28 | 1.35 | -0.05 |
| bcap29 | ENSDARG00000016231 | Ensembl | 0.19 | 1.24 | 0.78 | 0.70 | -0.13 |
| bhlhe40 | ENSDARG00000004060 | Ensembl | -0.80 | -1.04 | -1.39 | -0.13 | -0.34 |
| bhlhe40 | ENSDARG00000004060 | Ensembl | -0.65 | -0.98 | -1.40 | -0.18 | -0.37 |
| bhlhe40 | ENSDARG00000004060 | Ensembl | -0.88 | -0.92 | -1.24 | -0.10 | -0.41 |
| BICD2 | ENSORLG00000011196 | Ensembl | -0.40 | -0.66 | -0.74 | -0.01 | -1.05 |
| bmp5 | ENSDARG00000004965 | Ensembl | -1.16 | -0.63 | -0.54 | -0.53 | -0.07 |
| bmp5 | ENSDARG00000004965 | Ensembl | -1.24 | -0.93 | -0.93 | -0.51 | 0.13 |
| bmp5 | ENSDARG00000004965 | Ensembl | -1.28 | -0.87 | -0.66 | -0.64 | -0.09 |
| bmp5 | ENSDARG00000004965 | Ensembl | -1.27 | -0.61 | -1.02 | -0.59 | -0.01 |
| btbd9 | ENSDARG00000068983 | Ensembl | -0.77 | -0.25 | -1.24 | 0.03 | -0.47 |
| btr06 | ENSDARG00000054184 | Ensembl | 0.00 | 0.00 | 0.00 | 1.44 | 0.00 |
| btr20 | ENSDARG00000075603 | Ensembl | 0.46 | -0.61 | 0.33 | -5.21 | -0.65 |
| btr22 | ENSDARG00000093049 | Ensembl | -0.17 | -1.03 | -0.60 | -0.68 | -0.47 |
| btr26 | ENSDARG00000040860 | Ensembl | -0.18 | -0.94 | -0.14 | -5.22 | -0.61 |
| BX539340.1 | ENSDARG00000089797 | Ensembl | -0.76 | 0.32 | -1.71 | -4.54 | 0.00 |
| BX539340.1 | ENSDARG00000089797 | Ensembl | -0.81 | -2.04 | 0.67 | -0.45 | -0.44 |
| BX572630.2 | ENSDARG00000088251 | Ensembl | -0.39 | 0.25 | 0.31 | 1.79 | -0.08 |
| BX572630.2 | ENSDARG00000088251 | Ensembl | -0.60 | 0.17 | 0.61 | 2.08 | 0.19 |
| BX640584.1 | ENSDARG00000086955 | Ensembl | -0.24 | -0.97 | 0.26 | -2.18 | -0.72 |
| BX664721.5 | ENSMUSG00000070332 | Ensembl | 0.09 | -0.36 | 0.10 | -5.57 | -0.81 |
| BX927253.1 | ENSDARG00000089111 | Ensembl | 1.35 | 1.57 | 1.24 | 1.70 | 0.24 |
| C18H15orf39 | ENSDARG00000069168 | Ensembl | -0.47 | -0.92 | -0.36 | -0.09 | -0.03 |
| c1qtnf5 | ENSDARG00000056134 | Ensembl | -0.49 | -1.20 | -0.38 | -0.89 | -1.08 |
| C25H1orf51 (2 of 2) | ENSDARG00000088171 | Ensembl | 1.45 | 1.36 | 0.00 | 1.81 | 0.00 |
| C7 (1 of 2) | ENSDARG00000057121 | Ensembl | 0.65 | 2.52 | 1.84 | 2.39 | 0.23 |
| C7 (1 of 2) | ENSDARG00000057121 | Ensembl | 0.50 | 2.61 | 1.90 | 2.43 | 0.19 |
| C7 (1 of 2) | ENSDARG00000057121 | Ensembl | 0.75 | 2.81 | 2.19 | 2.52 | 0.06 |
| C7 (1 of 2) | ENSDARG00000057121 | Ensembl | 0.58 | 2.69 | 2.03 | 2.55 | 0.33 |
| C7 (1 of 2) | ENSDARG00000057121 | Ensembl | 0.67 | 2.78 | 2.15 | 2.56 | 0.29 |
| C7 (1 of 2) | ENSDARG00000057121 | Ensembl | 0.74 | 2.59 | 2.12 | 2.57 | 0.33 |
| C7 (1 of 2) | ENSDARG00000057121 | Ensembl | 0.94 | 2.75 | 2.10 | 2.61 | 0.25 |
| C7 (1 of 2) | ENSDARG00000057121 | Ensembl | 0.64 | 2.84 | 2.15 | 2.66 | 0.21 |
| CABZ01040055.1 | ENSDARG00000086869 | Ensembl | 1.69 | 2.40 | 1.92 | 0.55 | 2.42 |
| CABZ01055715.1 | ENSDARG00000087246 | Ensembl | 0.60 | -0.01 | 0.79 | 1.60 | 0.48 |
| CABZ01055715.1 | ENSDARG00000087246 | Ensembl | 0.18 | 0.84 | 0.32 | 0.47 | 0.14 |
| cacng6b | ENSDARG00000046079 | Ensembl | -1.21 | -0.33 | -0.77 | -0.54 | -0.77 |
| calcrla | ENSDARG00000011473 | Ensembl | 0.00 | 0.00 | 0.00 | 1.14 | 0.87 |
| CARD14 | ENSG00000141527 | Ensembl | 1.24 | 1.77 | 1.85 | 1.06 | 0.53 |
| CARD14 | ENSG00000141527 | Ensembl | 1.41 | 1.70 | 1.80 | 1.00 | 1.08 |
| cbln8 | ENSDARG00000019294 | Ensembl | 0.70 | 1.29 | 1.44 | 2.22 | -0.38 |
| ccl-c5a | ENSDARG00000058389 | Ensembl | 0.24 | 1.27 | 0.72 | 2.36 | -0.26 |
| ccrn4la | ENSDARG00000077726 | Ensembl | 0.67 | 0.39 | 0.23 | 0.60 | 0.80 |
| ccrn4la | ENSDARG00000077726 | Ensembl | 0.78 | 0.43 | 0.20 | 0.59 | 0.76 |
| cd40 | ENSDARG00000054968 | Ensembl | 1.05 | 2.58 | 1.55 | 0.85 | 0.91 |
| cd99l2 | ENSDARG00000056722 | Ensembl | -1.03 | -0.69 | -0.47 | -1.11 | -1.56 |
| cda | ENSDARG00000038199 | Ensembl | 1.17 | 1.82 | 1.53 | 1.21 | 1.05 |
| cda | ENSDARG00000038199 | Ensembl | 1.02 | 1.82 | 1.35 | 0.79 | 1.01 |
| cda | ENSDARG00000038199 | Ensembl | 0.77 | 1.56 | 1.41 | 0.92 | 0.85 |
| cda | ENSDARG00000038199 | Ensembl | 1.09 | 1.95 | 1.37 | 0.90 | 1.25 |
| cda | ENSDARG00000038199 | Ensembl | 0.84 | 1.71 | 1.05 | 0.58 | 0.87 |
| cda | ENSDARG00000038199 | Ensembl | 0.95 | 1.97 | 1.34 | 0.89 | 1.05 |
| CDR2 (1 of 2) | ENSDARG00000035952 | Ensembl | 0.16 | 0.37 | -2.03 | -0.72 | 0.20 |
| ch25h | ENSDARG00000045190 | Ensembl | 2.06 | 3.71 | 2.65 | 3.96 | 0.89 |
| chd4a | ENSDARG00000063535 | Ensembl | 0.36 | 1.00 | 0.38 | 0.55 | -0.35 |
| chka | ENSDARG00000041078 | Ensembl | 0.95 | 0.93 | 0.39 | 0.08 | 0.30 |
| chka | ENSDARG00000041078 | Ensembl | 1.00 | 0.52 | 0.28 | 0.05 | -0.15 |
| chka | ENSDARG00000041078 | Ensembl | 0.93 | 0.77 | 0.48 | 0.63 | -0.07 |
| chmp6b | ENSDARG00000021202 | Ensembl | 0.00 | 1.07 | 1.02 | 0.00 | 0.00 |
| chp1 | ENSDARG00000052859 | Ensembl | 0.37 | 0.96 | 0.50 | 0.49 | 0.21 |
| chrm4a | ENSDARG00000069254 | Ensembl | -1.27 | -1.02 | -0.07 | -2.00 | -0.03 |
| CLEC17A | ENSG00000187912 | Ensembl | 0.12 | 1.45 | 0.95 | 0.40 | 0.64 |
| CLEC17A | ENSG00000187912 | Ensembl | 0.18 | 1.51 | 0.98 | 0.55 | 0.66 |
| CLEC17A | ENSG00000187912 | Ensembl | 0.17 | 1.49 | 0.98 | 0.49 | 0.61 |
| CLEC17A | ENSG00000187912 | Ensembl | 0.16 | 1.48 | 0.96 | 0.47 | 0.62 |
| CLEC17A | ENSG00000187912 | Ensembl | 0.62 | 1.70 | 1.23 | 1.09 | 0.56 |
| CLEC17A | ENSG00000187912 | Ensembl | 0.23 | 1.55 | 1.01 | 0.62 | 0.59 |
| CLEC17A | ENSG00000187912 | Ensembl | 0.66 | 1.73 | 1.25 | 1.05 | 0.49 |
| clic2 | ENSDARG00000010625 | Ensembl | 0.51 | 0.90 | 1.04 | 0.33 | 0.17 |
| clstn1 | ENSDARG00000031720 | Ensembl | 0.61 | 0.73 | 0.53 | 1.27 | 0.03 |
| col13a1 | ENSG00000197467 | Ensembl | 0.00 | 0.00 | 0.00 | 0.00 | 1.77 |
| COL16A1 | ENSDARG00000009194 | Ensembl | 0.20 | -1.39 | 0.44 | 0.09 | 0.35 |
| col1a1a | ENSDARG00000012405 | Ensembl | 0.26 | -0.91 | -0.20 | 0.11 | -0.46 |
| col1a1a | ENSDARG00000012405 | Ensembl | 0.49 | -0.79 | -0.09 | 0.16 | -0.26 |
| col1a1a | ENSDARG00000012405 | Ensembl | 0.17 | -0.69 | -0.07 | 0.04 | -0.01 |
| col1a1a | ENSDARG00000012405 | Ensembl | 0.21 | -0.68 | -0.04 | 0.12 | 0.04 |
| col1a1b | ENSDARG00000035809 | Ensembl | -0.11 | -0.92 | -0.06 | 0.03 | 0.29 |
| col1a1b | ENSDARG00000035809 | Ensembl | 0.41 | -1.38 | -0.12 | -0.05 | 0.13 |
| col1a2 | ENSDARG00000020007 | Ensembl | 0.23 | -0.88 | -0.04 | 0.15 | -0.19 |
| col1a2 | ENSDARG00000020007 | Ensembl | 0.37 | -0.75 | 0.01 | 0.26 | -0.22 |
| col4a3bp | ENSDARG00000063542 | Ensembl | 1.17 | 1.87 | 1.14 | 1.05 | 0.23 |
| col6a2 | ENSDARG00000061436 | Ensembl | 0.16 | -0.76 | 0.03 | 0.03 | -0.28 |
| col6a3 | ENSDARG00000077139 | Ensembl | 0.02 | -0.80 | -0.21 | -0.03 | -0.28 |
| col8a1a | ENSDARG00000077403 | Ensembl | 0.25 | 0.32 | 0.90 | 1.64 | -0.49 |
| CR352328.2 | ENSDARG00000089432 | Ensembl | 0.72 | 0.27 | 0.46 | -7.13 | 0.03 |
| CR352328.2 | ENSDARG00000089432 | Ensembl | 0.71 | -0.10 | 0.52 | -6.92 | 0.07 |
| CR354432.1 | ENSDARG00000091579 | Ensembl | 0.62 | 1.44 | 0.46 | 0.88 | 0.37 |
| CR356230.1 | ENSDARG00000038872 | Ensembl | -0.59 | -0.75 | -1.35 | -0.05 | -0.81 |
| CR385063.1 | ENSDARG00000044212 | Ensembl | 0.00 | 1.11 | 0.45 | 0.00 | 0.49 |
| CREM | ENSMUSG00000063889 | Ensembl | 0.68 | 1.70 | 1.17 | 0.66 | 0.33 |
| CT055 | NM_001140483.1 | refseq | 4.84 | 5.40 | 4.31 | 2.07 | 0.00 |
| ctgfa | ENSDARG00000042934 | Ensembl | 0.62 | 1.37 | 0.97 | 1.49 | 1.19 |
| ctgfa | ENSDARG00000042934 | Ensembl | 0.27 | 1.20 | 0.84 | 1.34 | 1.02 |
| ctsh | ENSDARG00000041108 | Ensembl | -0.42 | -1.10 | -0.04 | -0.19 | 0.06 |
| CU019646.2 | ENSDARG00000091234 | Ensembl | 0.44 | 2.81 | 1.99 | 1.93 | -0.28 |
| CU459095.1 | ENSDARG00000086495 | Ensembl | -0.10 | -0.72 | -0.09 | -0.19 | 0.14 |
| cycsb | ENSDARG00000044562 | Ensembl | 1.08 | 2.09 | 0.88 | 1.95 | 0.95 |
| cycsb | ENSDARG00000044562 | Ensembl | 0.75 | 2.05 | 0.70 | 1.68 | 0.65 |
| cylda | ENSDARG00000060058 | Ensembl | 0.90 | 1.86 | 1.21 | 1.06 | 0.63 |
| cylda | ENSDARG00000060058 | Ensembl | 0.51 | 1.55 | 0.90 | 1.19 | 0.45 |
| cylda | ENSDARG00000060058 | Ensembl | 0.32 | 1.42 | 0.59 | 0.68 | 0.14 |
| cytidine | NM_001146593.1 | refseq | 1.03 | 1.69 | 1.34 | 0.68 | 0.79 |
| dcbld1 | ENSDARG00000015907 | Ensembl | 0.36 | 1.74 | 0.57 | 0.93 | 0.22 |
| ddit4 | ENSDARG00000037618 | Ensembl | -0.18 | 1.59 | 0.58 | 1.11 | 3.62 |
| ddit4 | ENSDARG00000037618 | Ensembl | 0.00 | 0.00 | 0.00 | 0.00 | 3.68 |
| ddit4 | ENSDARG00000037618 | Ensembl | 0.00 | 0.00 | 0.00 | 0.00 | 3.49 |
| ddit4l | ENSMUSG00000046818 | Ensembl | 1.21 | 0.79 | 0.12 | 2.07 | 0.19 |
| DDX17 | ENSDARG00000010873 | Ensembl | -0.22 | -0.84 | -0.16 | -0.20 | -0.13 |
| ddx5 | ENSDARG00000038068 | Ensembl | 0.84 | 1.54 | 1.62 | 1.45 | 1.41 |
| ddx5 | XM_003443093.1 | refseq | 1.07 | 2.03 | 1.72 | 1.33 | 1.40 |
| ddx5 | ENSDARG00000038068 | Ensembl | 1.19 | 1.82 | 1.74 | 1.37 | 1.38 |
| ddx5 | ENSDARG00000038068 | Ensembl | 1.08 | 2.14 | 1.74 | 1.35 | 1.27 |
| ddx5 | ENSDARG00000038068 | Ensembl | 0.91 | 2.17 | 1.55 | 1.06 | 1.06 |
| ddx5 | ENSDARG00000038068 | Ensembl | 1.08 | 2.14 | 1.77 | 1.33 | 1.30 |
| DDX5 | BT059556.1 | nt | 0.00 | 0.00 | 1.81 | 2.05 | 0.00 |
| desi1a | ENSDARG00000033140 | Ensembl | 0.29 | 0.99 | 0.49 | 0.61 | 0.05 |
| dhrs11a | ENSDARG00000046090 | Ensembl | 0.43 | 1.06 | 0.65 | 0.62 | 0.23 |
| dio2 | ENSDARG00000094857 | Ensembl | -1.51 | -1.70 | -2.61 | -1.26 | -0.84 |
| dio2 | ENSDARG00000094857 | Ensembl | -1.64 | -1.33 | -2.17 | -1.39 | -1.54 |
| dio2 | ENSDARG00000094857 | Ensembl | -1.19 | -0.95 | -1.80 | -1.10 | -0.58 |
| dio2 | ENSDARG00000094857 | Ensembl | -1.62 | -1.14 | -2.27 | -1.42 | -1.02 |
| dio2 | ENSDARG00000094857 | Ensembl | -1.49 | -1.22 | -1.88 | -1.07 | -0.78 |
| dio2 | ENSDARG00000094857 | Ensembl | -1.42 | -0.93 | -2.11 | -1.20 | -0.72 |
| dio3a | ENSDARG00000089937 | Ensembl | 1.16 | 2.24 | 2.25 | 1.90 | 1.55 |
| dio3a | ENSDARG00000089937 | Ensembl | -1.53 | 0.27 | 0.59 | -0.59 | 0.84 |
| dnajb11 | ENSDARG00000015088 | Ensembl | 1.59 | 1.52 | 1.42 | 3.19 | 0.15 |
| dnajb11 | ENSDARG00000015088 | Ensembl | 0.76 | 1.00 | 0.76 | 2.29 | -0.03 |
| dnajb11 | ENSDARG00000015088 | Ensembl | 0.80 | 1.07 | 0.90 | 2.35 | 0.09 |
| dnajb11 | ENSDARG00000015088 | Ensembl | 1.50 | 1.23 | 1.48 | 3.19 | 0.20 |
| DNAJB9 (1 of 2) | ENSDARG00000052072 | Ensembl | 1.09 | 1.91 | 1.30 | 1.95 | 0.37 |
| DNAJB9 (1 of 2) | ENSDARG00000052072 | Ensembl | 1.27 | 2.00 | 0.00 | 1.86 | 0.45 |
| DNAJB9 (1 of 2) | ENSDARG00000052072 | Ensembl | 1.52 | 2.15 | 1.53 | 1.82 | 0.71 |
| dnajc3 | ENSG00000102580 | Ensembl | 1.01 | 0.74 | 0.50 | 2.07 | -1.37 |
| dnajc3 | ENSG00000102580 | Ensembl | 0.72 | 0.63 | 0.59 | 1.56 | -0.25 |
| drg1 | ENSDARG00000039345 | Ensembl | 0.07 | 0.69 | 0.41 | 0.51 | 0.38 |
| dusp1 | ENSDARG00000007628 | Ensembl | 0.15 | 2.24 | 1.17 | 1.95 | 0.77 |
| dusp2 | ENSDARG00000007628 | Ensembl | 0.57 | 1.78 | 0.82 | 1.94 | 0.43 |
| egr1 | ENSDARG00000037421 | Ensembl | 0.61 | 2.44 | 0.71 | 3.21 | 0.17 |
| egr1 | ENSDARG00000037421 | Ensembl | 0.92 | 2.85 | 0.96 | 3.71 | 0.00 |
| eif1b | BT056792.1 | nt | 0.56 | 1.80 | 1.38 | 0.85 | 0.77 |
| eif1b | ENSDARG00000012688 | Ensembl | 0.25 | 1.29 | 0.86 | 0.50 | 0.34 |
| eif1b | ENSDARG00000012688 | Ensembl | 0.27 | 1.14 | 0.76 | 0.49 | 0.31 |
| eif1b | ENSDARG00000012688 | Ensembl | 0.16 | 1.24 | 0.72 | 0.44 | 0.24 |
| eif1b | ENSDARG00000012688 | Ensembl | 0.20 | 1.17 | 0.83 | 0.39 | 0.21 |
| eif1b | ENSDARG00000012688 | Ensembl | 0.31 | 1.17 | 0.85 | 0.48 | 0.20 |
| eif1b | ENSDARG00000012688 | Ensembl | 0.38 | 1.29 | 0.86 | 0.57 | 0.25 |
| eif1b | ENSDARG00000012688 | Ensembl | 0.28 | 1.23 | 0.82 | 0.42 | 0.25 |
| eif1b | ENSDARG00000012688 | Ensembl | 0.25 | 1.26 | 0.83 | 0.48 | 0.26 |
| eif1b | ENSDARG00000012688 | Ensembl | 0.40 | 1.08 | 0.68 | 0.31 | 0.27 |
| eif1b | ENSDARG00000012688 | Ensembl | 0.30 | 1.18 | 0.92 | 0.51 | 0.42 |
| eif1b | ENSDARG00000012688 | Ensembl | 0.14 | 1.13 | 0.78 | 0.39 | 0.24 |
| eif1b | ENSDARG00000012688 | Ensembl | 0.29 | 0.86 | 0.33 | 0.16 | 0.06 |
| eif1b | ENSDARG00000012688 | Ensembl | 0.23 | 1.10 | 0.74 | 0.43 | 0.09 |
| eif1b | ENSDARG00000012688 | Ensembl | 0.26 | 1.05 | 0.71 | 0.58 | 0.15 |
| eif1b | ENSDARG00000012688 | Ensembl | 0.22 | 1.13 | 0.78 | 0.41 | 0.29 |
| eif4g1 | ENSG00000114867 | Ensembl | 1.00 | 0.65 | 0.77 | 1.00 | 0.48 |
| eif4g2a | ENSDARG00000020377 | Ensembl | 0.91 | 1.38 | 1.04 | 0.88 | 0.67 |
| eif4g2a | ENSDARG00000020377 | Ensembl | 0.74 | 1.23 | 1.06 | 0.68 | 0.48 |
| eif4g2a | ENSDARG00000020377 | Ensembl | 0.38 | 1.23 | 1.00 | 0.65 | 0.48 |
| eif4g2a | ENSDARG00000020377 | Ensembl | 0.61 | 1.00 | 1.05 | 0.70 | 0.24 |
| eif4g2a | ENSDARG00000020377 | Ensembl | 0.55 | 0.99 | 0.47 | 0.35 | 0.50 |
| eif4g2a | ENSDARG00000020377 | Ensembl | 0.68 | 1.22 | 0.77 | 0.93 | 0.17 |
| elvol5a | GU238431.1 | nt | 0.74 | 3.65 | 1.36 | 1.81 | 0.52 |
| enpp7 | ENSDARG00000077225 | Ensembl | 1.43 | 1.96 | 1.24 | 1.87 | 1.27 |
| ERGIC1 | ENSDARG00000005273 | Ensembl | 0.56 | 0.85 | 0.77 | 1.27 | 0.26 |
| evpla | ENSDARG00000019808 | Ensembl | 0.39 | 1.27 | 1.52 | 0.15 | 2.15 |
| evpla | ENSDARG00000019808 | Ensembl | 0.79 | 1.50 | 1.17 | 0.20 | 2.04 |
| fam108b1 | ENSDARG00000035571 | Ensembl | 0.00 | 1.29 | 1.01 | 0.00 | 0.00 |
| fam20a | ENSDARG00000079486 | Ensembl | -0.33 | -0.79 | -0.37 | -0.98 | -0.38 |
| fbxl5 | ENSDARG00000043046 | Ensembl | 0.87 | 1.71 | 1.19 | 1.37 | 0.49 |
| fbxl5 | ENSDARG00000043046 | Ensembl | 0.94 | 1.58 | 1.34 | 1.34 | 0.39 |
| fbxl5 | ENSDARG00000043046 | Ensembl | 0.88 | 1.35 | 0.69 | 1.32 | 0.60 |
| fbxl5 | ENSDARG00000043046 | Ensembl | 0.66 | 1.45 | 1.16 | 1.11 | 0.29 |
| fbxl5 | ENSDARG00000043046 | Ensembl | 0.84 | 1.38 | 0.91 | 1.38 | 0.35 |
| Fc | ENSMUSG00000015947 | Ensembl | 1.24 | 0.91 | 1.36 | 2.01 | -0.09 |
| FDFT1 | ENSDARG00000060260 | Ensembl | 1.69 | 1.32 | 0.30 | 1.28 | -0.26 |
| fdx1 | ENSDARG00000056410 | Ensembl | 1.75 | 1.77 | 0.03 | 2.35 | 0.51 |
| ficd | ENSDARG00000035595 | Ensembl | 0.98 | 1.25 | 1.06 | 2.12 | 0.21 |
| fnbp1 | ENSDARG00000036156 | Ensembl | -0.71 | -1.41 | -1.51 | -0.85 | -0.57 |
| fnbp1 | ENSDARG00000036156 | Ensembl | -0.68 | -1.52 | -1.94 | -0.57 | -0.70 |
| fnbp1 | ENSDARG00000036156 | Ensembl | -0.32 | -1.34 | -2.21 | -0.76 | -0.38 |
| FNDC3A | ENSDARG00000067569 | Ensembl | 0.89 | 0.44 | -0.01 | 0.71 | -0.06 |
| fosl2 | ENSDARG00000040623 | Ensembl | 1.87 | 4.41 | 2.83 | 3.34 | 1.62 |
| fosl2 | ENSDARG00000040623 | Ensembl | 1.18 | 3.24 | 2.25 | 2.74 | 0.93 |
| fosl2 | ENSDARG00000040623 | Ensembl | 1.44 | 3.31 | 2.13 | 2.79 | 0.00 |
| FOXO4 | ENSDARG00000055792 | Ensembl | 0.82 | 0.83 | 0.61 | 0.41 | 0.12 |
| fructose | NM_001173920.1 | refseq | 1.07 | 2.39 | 1.27 | 1.35 | 0.59 |
| fth1b | ENSDARG00000007975 | Ensembl | 0.72 | 1.21 | 1.88 | 0.85 | 0.96 |
| fxyd5b | NM_001123724.1 | refseq | 0.69 | 2.97 | 1.48 | 1.90 | 0.14 |
| FYB (1 of 2) | ENSDARG00000044694 | Ensembl | 0.00 | 0.00 | 1.19 | 0.00 | 0.00 |
| G0S2 | BT046903.1_ | nt | 1.87 | 5.06 | 3.10 | 4.58 | -0.07 |
| G0S2 | BT046903.1_ | nt | 2.13 | 5.13 | 3.21 | 4.76 | 0.61 |
| gadd45a | BT046750.2 | nt | -0.64 | -1.07 | -1.07 | -0.79 | -0.75 |
| gadd45ab | ENSDARG00000069991 | Ensembl | -0.01 | -0.26 | -0.01 | -0.96 | 0.20 |
| gata5 | ENSDARG00000017821 | Ensembl | -1.15 | -2.41 | -0.78 | -1.32 | -0.01 |
| gck | ENSDARG00000068006 | Ensembl | 3.40 | 1.53 | -2.78 | -0.72 | 0.56 |
| gclm | ENSDARG00000018953 | Ensembl | 0.97 | 1.19 | 0.20 | 0.73 | 0.34 |
| gclm | ENSDARG00000018953 | Ensembl | 0.72 | 1.17 | 0.41 | 0.59 | 0.20 |
| GIMAP4 | ENSG00000133574 | Ensembl | -1.50 | -2.47 | -2.10 | -6.23 | -1.72 |
| GIMAP4 | ENSG00000133574 | Ensembl | 1.14 | 1.73 | 1.00 | 1.01 | 0.11 |
| GIMAP9 | ENSMUSG00000051124 | Ensembl | 1.06 | 1.42 | 0.00 | 0.60 | 0.00 |
| glcci1 | ENSDARG00000008503 | Ensembl | 0.53 | 0.58 | 0.74 | 0.96 | 0.54 |
| glcci1 | ENSDARG00000008503 | Ensembl | 0.60 | 0.45 | 0.55 | 0.87 | 0.48 |
| glcci1 | ENSDARG00000008503 | Ensembl | 0.82 | 0.75 | 0.80 | 1.18 | 0.60 |
| glcci1 | ENSDARG00000008503 | Ensembl | 0.44 | 0.46 | 0.68 | 0.94 | 0.07 |
| glyg | NM_001139830.1 | refseq | 0.20 | 0.99 | 0.37 | 2.12 | 0.01 |
| gmppb | ENSDARG00000017658 | Ensembl | 0.63 | 1.16 | 0.62 | 2.07 | -0.09 |
| gnai1 | ENSDARG00000021647 | Ensembl | -0.99 | -1.06 | -0.78 | -0.33 | -0.89 |
| gopc | ENSDARG00000023117 | Ensembl | 1.04 | 2.45 | 1.90 | 2.01 | 0.74 |
| gopc | ENSDARG00000023117 | Ensembl | 0.79 | 1.76 | 1.43 | 1.55 | 0.63 |
| gopc | ENSDARG00000023117 | Ensembl | 1.09 | 2.32 | 1.66 | 1.86 | 0.38 |
| gopc | ENSDARG00000023117 | Ensembl | 0.95 | 1.92 | 1.43 | 1.28 | 0.15 |
| gpd1b | ENSDARG00000043180 | Ensembl | -0.43 | -0.91 | -0.29 | -0.54 | -0.02 |
| gpd1b | ENSDARG00000043180 | Ensembl | 0.04 | -1.00 | -0.16 | -0.82 | -0.08 |
| Grik2 | ENSMUSG00000056073 | Ensembl | -0.23 | -1.57 | 0.03 | -0.24 | 0.47 |
| gsr | ENSDARG00000019236 | Ensembl | 0.41 | 0.86 | 0.29 | 0.19 | -0.12 |
| gsr | ENSDARG00000019236 | Ensembl | 0.53 | 0.94 | 0.29 | 0.32 | -0.04 |
| gys2 | ENSDARG00000004904 | Ensembl | -0.13 | -0.82 | -0.11 | -0.20 | -0.28 |
| hdac4 | ENSDARG00000041204 | Ensembl | -0.47 | -0.69 | -0.47 | -1.34 | -0.42 |
| HECT | ENSG00000138646 | Ensembl | -0.69 | 0.31 | 0.70 | 1.90 | 0.21 |
| hect | ENSMUSG00000029804 | Ensembl | -0.10 | 0.32 | 0.68 | 1.54 | 0.00 |
| HELZ2 (2 of 2) | ENSDARG00000016527 | Ensembl | -0.66 | 0.23 | 0.37 | 2.35 | 0.30 |
| HELZ2 (2 of 2) | ENSDARG00000016527 | Ensembl | 0.22 | 0.13 | 1.06 | 1.58 | -0.24 |
| HELZ2 (2 of 2) | ENSDARG00000016527 | Ensembl | 0.00 | 0.00 | 0.00 | 1.97 | 0.00 |
| HERC4 | ENSG00000138642 | Ensembl | -0.73 | 0.05 | 0.42 | 1.67 | -0.18 |
| HERC4 | ENSG00000138642 | Ensembl | -0.88 | -0.27 | -0.06 | 1.75 | 0.07 |
| HERC4 | ENSG00000138642 | Ensembl | -0.66 | -0.19 | 0.49 | 1.77 | 0.12 |
| herp2 | NM_001146672.1 | refseq | 1.30 | 1.74 | 1.72 | 1.22 | 0.00 |
| HHEX | ENSG00000152804 | Ensembl | -0.54 | -0.81 | -0.57 | -0.12 | -0.05 |
| hig1 | ENSDARG00000022303 | Ensembl | 0.74 | 2.10 | 0.86 | 1.45 | 0.77 |
| hig1 | ENSDARG00000022303 | Ensembl | 0.39 | 1.82 | 0.75 | 1.43 | 0.57 |
| hig1 | ENSDARG00000022303 | Ensembl | 0.26 | 1.80 | 0.96 | 1.27 | 0.60 |
| hmox1 | ENSDARG00000027529 | Ensembl | 0.76 | 1.61 | 0.94 | 1.02 | 0.55 |
| hmox1 | ENSDARG00000027529 | Ensembl | 0.70 | 1.43 | 0.84 | 0.74 | 0.32 |
| hmox1 | ENSDARG00000027529 | Ensembl | 0.89 | 1.47 | 0.96 | 0.74 | 0.32 |
| homez | ENSDARG00000054304 | Ensembl | 0.58 | 1.73 | 0.82 | 1.05 | 0.43 |
| homez | ENSDARG00000054304 | Ensembl | 0.92 | 1.76 | 0.86 | 0.72 | 0.57 |
| HoxD | EU025718.1 | nt | 0.69 | 1.31 | 1.66 | 1.70 | 0.59 |
| hpcl1 | NM_001173876.1 | refseq | -0.92 | -0.97 | -0.79 | -0.64 | -0.13 |
| hspa13 | ENSDARG00000040984 | Ensembl | 0.00 | 0.00 | 0.00 | 1.85 | 0.00 |
| hspa5 | ENSDARG00000004665 | Ensembl | 1.14 | 1.55 | 1.31 | 2.40 | -0.06 |
| hspa5 | ENSDARG00000004665 | Ensembl | 1.23 | 1.57 | 1.39 | 2.46 | 0.02 |
| hspa5 | ENSDARG00000004665 | Ensembl | 0.94 | 1.25 | 1.14 | 2.10 | -0.16 |
| hspb2 | ENSDARG00000052450 | Ensembl | 0.08 | -1.15 | -0.14 | -0.34 | -0.11 |
| huwe1 | ENSDARG00000016782 | Ensembl | 0.43 | 0.94 | 0.49 | 0.60 | 0.24 |
| hyou1 | ENSDARG00000013670 | Ensembl | 0.60 | 0.75 | 0.63 | 2.31 | -0.49 |
| hyou1 | ENSDARG00000013670 | Ensembl | 0.60 | 0.67 | 0.81 | 2.39 | -0.41 |
| hyou1 | ENSDARG00000013670 | Ensembl | 0.83 | 0.77 | 0.86 | 2.66 | -0.22 |
| hyou1 | ENSDARG00000013670 | Ensembl | 0.84 | 0.36 | 0.81 | 2.40 | 0.00 |
| hyou1 | ENSDARG00000013670 | Ensembl | 0.51 | 0.80 | 0.59 | 2.44 | 0.00 |
| hyou1 | ENSDARG00000013670 | Ensembl | 0.64 | 0.77 | 0.55 | 2.50 | 0.00 |
| hyou1 | ENSDARG00000013670 | Ensembl | 0.00 | 1.12 | 0.71 | 2.58 | 0.00 |
| hyou1 | ENSDARG00000013670 | Ensembl | 0.00 | 0.63 | 1.12 | 2.69 | 0.00 |
| hyou1 | ENSDARG00000013670 | Ensembl | 1.14 | 0.00 | 1.33 | 2.92 | 0.00 |
| id1 | ENSDARG00000040764 | Ensembl | -0.70 | -0.99 | -0.29 | -0.37 | -0.39 |
| id2 | ENSDARG00000040764 | Ensembl | -0.87 | -0.70 | -0.34 | -0.28 | -0.68 |
| id2a | ENSDARG00000055283 | Ensembl | -1.46 | -0.26 | -0.13 | 0.20 | -0.11 |
| idi1 | ENSDARG00000019976 | Ensembl | 0.60 | 0.66 | 0.11 | 0.43 | -0.08 |
| ifit5 | ENSG00000152778 | Ensembl | -0.59 | 0.51 | 0.73 | 2.19 | -0.51 |
| igfbp1a | ENSDARG00000014947 | Ensembl | 1.38 | 1.07 | 0.62 | 3.36 | 1.67 |
| igfbp1a | ENSDARG00000014947 | Ensembl | 1.74 | 1.66 | 1.03 | 3.28 | 1.50 |
| ihhb | ENSDARG00000058815 | Ensembl | -0.62 | -0.76 | -0.41 | -0.31 | -0.16 |
| IL10RB | ENSDARG00000068711 | Ensembl | 0.20 | 0.93 | 0.42 | 0.61 | 0.06 |
| il17r | NM_001165364.1 | refseq | 0.91 | 1.83 | 1.55 | 0.93 | 0.34 |
| IL17RA | ENSDARG00000058244 | Ensembl | 1.01 | 2.01 | 1.67 | 0.98 | 0.31 |
| il4r | ENSDARG00000031051 | Ensembl | 0.98 | 1.56 | 1.08 | 0.44 | 0.19 |
| insig1 | ENSDARG00000010658 | Ensembl | 0.00 | 1.33 | 0.00 | 0.74 | 0.00 |
| ipmkb | ENSDARG00000029291 | Ensembl | 0.00 | 0.91 | 0.00 | 0.00 | 0.00 |
| irak3 | ENSDARG00000053131 | Ensembl | 0.00 | 1.23 | 0.00 | 0.00 | 0.00 |
| irf7 | ENSDARG00000045661 | Ensembl | 0.00 | 0.00 | 1.05 | 2.31 | 0.00 |
| irk11 | NM_001173964.1 | refseq | 2.27 | 2.43 | 2.60 | 2.52 | 1.45 |
| IRS2 | ENSDARG00000037099 | Ensembl | 0.70 | 0.73 | 1.29 | 0.44 | 0.57 |
| IRS4 (1 of 2) | ENSDARG00000052065 | Ensembl | 0.00 | 1.12 | 1.07 | 0.79 | 0.00 |
| ITCH (1 of 2) | ENSDARG00000076149 | Ensembl | 0.34 | 0.78 | 0.31 | 0.28 | -0.23 |
| ITGAX | ENSG00000140678 | Ensembl | 1.30 | 0.97 | 0.96 | 0.93 | -0.14 |
| itln1 | ENSG00000158764 | Ensembl | 1.46 | 2.66 | 2.23 | 1.92 | 0.71 |
| jak1 | ENSDARG00000020625 | Ensembl | 0.78 | 1.52 | 0.86 | 0.95 | 0.25 |
| jak1 | ENSDARG00000020625 | Ensembl | 0.57 | 1.20 | 0.71 | 0.52 | 0.53 |
| junba | ENSDARG00000074378 | Ensembl | 1.14 | 2.15 | 1.64 | 2.45 | 1.01 |
| junba | ENSDARG00000074378 | Ensembl | 1.24 | 2.27 | 1.68 | 2.66 | 1.06 |
| kifap3a | ENSDARG00000008639 | Ensembl | 0.14 | -1.30 | -0.02 | -0.12 | -0.45 |
| lama4 | ENSDARG00000020785 | Ensembl | -0.25 | -0.97 | -0.31 | -0.15 | -0.11 |
| lamb1b | ENSDARG00000045524 | Ensembl | -0.35 | -0.61 | -0.48 | -0.14 | -0.29 |
| laptm4b | ENSDARG00000035870 | Ensembl | 0.36 | 0.82 | 0.52 | 0.40 | 0.40 |
| lectin | NM_001123579.1 | refseq | 0.18 | 1.66 | 1.04 | 0.55 | 0.46 |
| leptin | NM_001145890.1 | refseq | 1.50 | 3.81 | 2.23 | 4.05 | 0.85 |
| leptin | NM_001145890.1 | refseq | 0.69 | 3.04 | 1.47 | 3.26 | 0.19 |
| leptin | GU584004.1 | nt | 1.05 | 3.41 | 1.93 | 3.70 | 0.00 |
| leucine | ENSG00000188993 | Ensembl | 0.92 | 0.81 | 0.81 | 0.20 | 0.15 |
| long | NM_001173689.1 | refseq | 0.86 | 1.09 | 1.00 | 0.91 | 0.46 |
| lpar5b | ENSDARG00000068638 | Ensembl | -0.20 | -1.28 | -0.51 | -0.70 | -0.24 |
| lpcat3 | ENSDARG00000075178 | Ensembl | 0.75 | 1.04 | 1.08 | 0.60 | 0.82 |
| lpcat3 | ENSDARG00000075178 | Ensembl | 0.84 | 1.02 | 0.84 | 0.66 | 0.56 |
| lphn3.1 | ENSDARG00000061121 | Ensembl | -0.77 | -0.41 | -1.20 | 0.09 | -0.42 |
| lrpprc | ENSDARG00000043970 | Ensembl | 0.24 | 0.79 | 0.22 | 0.61 | 0.62 |
| LRRC16B | ENSDARG00000086990 | Ensembl | -1.30 | -1.07 | -1.46 | -0.18 | -1.04 |
| LRRC73 | ENSDARG00000063411 | Ensembl | -1.05 | -0.66 | -1.26 | -0.23 | -0.65 |
| manf | ENSDARG00000063177 | Ensembl | 0.70 | 0.86 | 0.64 | 2.14 | 0.05 |
| MAP3K6 | ENSDARG00000069933 | Ensembl | 0.18 | 0.67 | -0.08 | 0.88 | -0.24 |
| mapk14a | ENSDARG00000000857 | Ensembl | 0.26 | 1.57 | 1.18 | 1.10 | 0.03 |
| mapk14b | ENSDARG00000028721 | Ensembl | 0.43 | 1.41 | 0.81 | 0.66 | 0.07 |
| mapre1b | ENSDARG00000002659 | Ensembl | 0.00 | 1.05 | 0.00 | 0.00 | 0.00 |
| marco | ENSDARG00000059294 | Ensembl | 0.28 | -0.06 | -0.33 | 1.41 | -0.30 |
| mat2aa | ENSDARG00000040334 | Ensembl | 0.72 | 2.39 | 1.51 | 1.88 | 0.22 |
| mat2aa | ENSDARG00000040334 | Ensembl | 0.63 | 1.72 | 1.11 | 1.34 | 0.47 |
| mat2aa | ENSDARG00000040334 | Ensembl | 0.68 | 1.71 | 0.77 | 0.82 | 0.60 |
| mat2aa | ENSDARG00000040334 | Ensembl | 0.52 | 1.42 | 0.43 | 0.76 | 0.30 |
| matn4 | ENSDARG00000015947 | Ensembl | 0.00 | 0.00 | 0.00 | 2.65 | 0.00 |
| matn4 | ENSDARG00000015947 | Ensembl | 0.00 | 0.00 | 0.00 | 2.92 | 0.00 |
| mcf2l | ENSDARG00000075859 | Ensembl | -1.17 | -1.27 | -1.45 | -1.18 | -0.85 |
| mcf2l | ENSDARG00000075859 | Ensembl | -0.99 | -1.29 | -1.25 | -0.70 | -0.64 |
| mcf2l | ENSDARG00000075859 | Ensembl | -0.86 | -1.12 | -1.05 | -0.61 | -0.96 |
| mcf2l | ENSDARG00000075859 | Ensembl | -1.20 | -1.39 | -1.05 | -0.74 | -0.66 |
| mcm6 | ENSDARG00000057683 | Ensembl | -0.85 | -1.00 | -0.73 | -0.35 | -0.21 |
| mertk | ENSDARG00000074695 | Ensembl | 1.33 | 0.00 | 0.00 | 2.06 | 0.00 |
| mfsd12a | ENSDARG00000061908 | Ensembl | 0.76 | 1.05 | 1.01 | 0.84 | 0.39 |
| MGAM | ENSG00000259858 | Ensembl | 1.55 | 1.64 | 0.56 | 0.49 | 1.16 |
| MGAM | ENSMUSG00000068587 | Ensembl | 0.00 | 1.84 | 0.00 | 0.00 | 0.00 |
| mgt4a | NM_001173641.1 | refseq | -2.92 | -1.94 | -0.86 | -2.50 | -0.50 |
| mia3 | ENSDARG00000008184 | Ensembl | -0.17 | 0.00 | 2.66 | 0.13 | 0.37 |
| micall2b | ENSDARG00000017834 | Ensembl | 1.49 | 1.45 | 1.27 | 1.56 | 0.44 |
| mid1ip1l | ENSDARG00000018145 | Ensembl | -1.07 | -0.57 | -0.94 | -0.41 | -0.28 |
| mknk1 | ENSDARG00000018411 | Ensembl | 0.96 | 2.67 | 1.55 | 1.73 | 0.76 |
| mknk1 | ENSDARG00000018411 | Ensembl | 0.76 | 2.47 | 1.30 | 1.41 | 0.60 |
| mknk2b | ENSDARG00000015164 | Ensembl | 0.67 | 0.83 | 0.87 | 0.74 | 0.20 |
| mknk2b | ENSDARG00000015164 | Ensembl | 0.77 | 0.73 | 1.06 | 0.77 | 0.43 |
| mknk2b | ENSDARG00000015164 | Ensembl | 0.66 | 0.86 | 0.97 | 0.72 | 0.35 |
| mll | ENSDARG00000004537 | Ensembl | -0.31 | -0.37 | -1.11 | -0.25 | 0.02 |
| mmp13a | ENSDARG00000012395 | Ensembl | 1.20 | 2.40 | 1.80 | 0.51 | -0.75 |
| mmp13a | ENSDARG00000012395 | Ensembl | 1.30 | 2.21 | 1.76 | 0.41 | 0.01 |
| mmp13a | ENSDARG00000012395 | Ensembl | 1.02 | 2.31 | 0.00 | 0.00 | 0.00 |
| MMP19 | ENSDARG00000091557 | Ensembl | -0.27 | 2.83 | 1.01 | 0.94 | -0.54 |
| mmp9 | ENSDARG00000042816 | Ensembl | 1.35 | 2.76 | 2.07 | 0.94 | -0.71 |
| mmp9 | ENSDARG00000042816 | Ensembl | 1.65 | 0.00 | 2.61 | 1.59 | 0.00 |
| mmp9 | ENSDARG00000042816 | Ensembl | 1.60 | 3.34 | 2.40 | 0.00 | 0.00 |
| MPPED2 (1 of 2) | ENSDARG00000006889 | Ensembl | -1.05 | -0.55 | -0.69 | -0.52 | -0.80 |
| mtmr8 | ENSDARG00000008592 | Ensembl | 1.11 | 1.16 | 1.44 | 0.72 | -0.01 |
| mx2 | ENSDARG00000004953 | Ensembl | 0.00 | 0.00 | 0.00 | 2.44 | 0.00 |
| mych | ENSDARG00000077473 | Ensembl | 0.50 | 1.84 | 0.60 | 2.08 | 0.14 |
| nadkb | ENSDARG00000060362 | Ensembl | 0.78 | 1.74 | 1.64 | 1.54 | 0.41 |
| nadkb | ENSDARG00000060362 | Ensembl | 0.86 | 1.78 | 1.56 | 1.63 | 0.32 |
| napa | ENSDARG00000020405 | Ensembl | 0.40 | 0.74 | 0.20 | 0.38 | 0.04 |
| napepld | ENSDARG00000009252 | Ensembl | 0.19 | 0.73 | 0.40 | 0.33 | -0.13 |
| NAV2 | ENSG00000166833 | Ensembl | -0.70 | -1.33 | -0.77 | -0.83 | 0.07 |
| NAV2 | ENSMUSG00000052512 | Ensembl | -0.73 | -1.49 | -0.48 | -0.58 | 0.14 |
| ncf1 | ENSDARG00000033735 | Ensembl | 0.00 | 0.99 | 1.42 | 0.00 | 0.00 |
| ncor1 | ENSDARG00000035285 | Ensembl | -0.61 | -0.38 | -1.09 | -0.41 | -0.57 |
| NDUFA4L2 (2 of 2) | ENSDARG00000087907 | Ensembl | -2.06 | -1.06 | -1.01 | -1.19 | -0.82 |
| NEDD5 | XM_004079979.1 | refseq | 0.76 | 1.39 | 1.19 | 0.73 | 0.15 |
| nfil3-5 | ENSDARG00000094965 | Ensembl | 0.63 | 0.74 | 0.14 | 0.45 | 0.84 |
| nfkb2 | ENSDARG00000038687 | Ensembl | 0.41 | 1.65 | 1.36 | 1.17 | 0.17 |
| nfkb2 | ENSDARG00000038687 | Ensembl | 0.57 | 1.01 | 0.66 | 1.00 | 0.41 |
| nfkb2 | ENSDARG00000038687 | Ensembl | 0.18 | 1.11 | 0.33 | 0.66 | -0.10 |
| nfkb2 | ENSDARG00000038687 | Ensembl | 0.24 | 1.21 | 0.21 | 0.80 | -0.24 |
| NID1 (2 of 2) | ENSDARG00000060675 | Ensembl | 0.71 | 2.66 | 1.26 | 0.96 | 0.00 |
| nlrc3 | ENSG00000167984 | Ensembl | 0.55 | 1.17 | 1.57 | 1.51 | 0.51 |
| nlrc3 | ENSG00000167984 | Ensembl | 0.77 | 1.17 | 1.63 | 1.59 | 0.06 |
| nlrc3 | ENSG00000167984 | Ensembl | 0.00 | 1.39 | 1.67 | 1.27 | 0.00 |
| nod2 | ENSDARG00000010756 | Ensembl | 0.38 | 1.10 | 0.33 | 0.60 | -0.06 |
| nots | ENSDARG00000052792 | Ensembl | 0.41 | 0.23 | 0.24 | 0.36 | 1.03 |
| NOXO1 | ENSONIG00000002316 | Ensembl | 0.00 | 1.82 | 0.00 | 0.00 | 0.00 |
| noxo1a | ENSDARG00000041294 | Ensembl | 0.17 | 1.42 | 0.24 | 1.40 | -0.28 |
| npsn | ENSDARG00000010423 | Ensembl | 1.31 | 1.25 | 1.92 | 1.31 | 0.12 |
| nr1h4 | ENSDARG00000057741 | Ensembl | 0.71 | 1.40 | 0.87 | 0.55 | 0.78 |
| nr1h4 | ENSDARG00000057741 | Ensembl | 0.78 | 1.43 | 0.88 | 0.66 | 0.67 |
| nr1h4 | ENSDARG00000057741 | Ensembl | 0.61 | 1.35 | 0.79 | 0.45 | 0.65 |
| nr1h4 | ENSDARG00000057741 | Ensembl | 0.42 | 1.15 | 0.55 | 0.38 | 0.55 |
| nr1h4 | ENSDARG00000057741 | Ensembl | 0.65 | 1.40 | 0.98 | 0.43 | 0.72 |
| nr1h4 | ENSDARG00000057741 | Ensembl | 0.95 | 1.62 | 1.10 | 0.72 | 0.81 |
| nr1h4 | ENSDARG00000057741 | Ensembl | 0.70 | 1.48 | 0.80 | 0.53 | 0.59 |
| nr2f1a | ENSDARG00000052695 | Ensembl | -0.44 | -0.81 | -0.56 | -0.20 | -0.18 |
| nr2f5 | ENSDARG00000033172 | Ensembl | 0.43 | 0.63 | 0.26 | 0.05 | 0.17 |
| Ocln-001 | ENSMUSG00000021638 | Ensembl | -0.82 | -0.28 | -2.12 | 0.05 | -0.83 |
| odc1 | ENSDARG00000007377 | Ensembl | 0.40 | 1.25 | 0.32 | 0.53 | 0.42 |
| odc1 | ENSDARG00000007377 | Ensembl | 0.03 | 1.32 | -0.01 | 0.20 | 0.21 |
| odc1 | ENSDARG00000007377 | Ensembl | 0.07 | 1.26 | 0.00 | 0.22 | 0.19 |
| odc1 | ENSDARG00000007377 | Ensembl | 0.00 | 1.31 | 0.13 | 0.38 | 0.02 |
| optn | ENSDARG00000002663 | Ensembl | 0.74 | 1.47 | 0.00 | 0.74 | 0.68 |
| ORC2 | ENSDARG00000090203 | Ensembl | 0.75 | 0.00 | 0.00 | 1.05 | 0.00 |
| pank1a | ENSDARG00000008192 | Ensembl | 0.29 | 0.93 | 0.55 | 0.45 | -0.17 |
| paternally | ENSMUSG00000092035 | Ensembl | -0.74 | -0.89 | -0.35 | -0.19 | -0.36 |
| pdcd6 | ENSDARG00000005220 | Ensembl | 0.28 | 1.03 | 0.63 | 0.64 | 0.45 |
| pglyrp6 | ENSDARG00000015626 | Ensembl | 0.42 | 1.09 | -0.23 | 3.34 | 0.00 |
| phactr4a | ENSDARG00000015552 | Ensembl | -0.29 | -0.80 | -0.14 | -0.13 | -0.03 |
| phkb | ENSDARG00000078284 | Ensembl | -0.28 | -0.21 | -0.61 | -0.60 | -0.96 |
| phkb | ENSDARG00000078284 | Ensembl | -0.60 | -0.44 | -0.63 | -1.24 | -0.21 |
| phop2 | NM_001139857.1 | refseq | 0.84 | 1.32 | 1.47 | 1.68 | 0.80 |
| phospho2 | ENSDARG00000058675 | Ensembl | 0.51 | 1.14 | 0.82 | 1.13 | 0.56 |
| pik3cg | ENSDARG00000017757 | Ensembl | 0.69 | 1.24 | 0.58 | 1.14 | 0.32 |
| pik3r3b | ENSDARG00000034409 | Ensembl | -1.20 | -0.44 | -0.69 | -0.43 | -0.72 |
| pim1 | ENSDARG00000059120 | Ensembl | 0.80 | 1.37 | 0.83 | 1.49 | 0.55 |
| PIM1 | ENSDARG00000059120 | Ensembl | 0.64 | 1.27 | 0.60 | 1.41 | 0.50 |
| pion | ENSDARG00000045481 | Ensembl | -0.55 | -0.28 | -0.64 | -0.55 | -1.28 |
| pisd | ENSDARG00000052462 | Ensembl | 1.32 | 2.19 | 1.57 | 1.71 | -0.72 |
| pisd | ENSDARG00000052462 | Ensembl | 1.53 | 2.13 | 1.58 | 1.79 | -0.66 |
| pisd | ENSDARG00000052462 | Ensembl | 1.39 | 2.02 | 1.61 | 1.60 | -0.09 |
| pisd | ENSDARG00000052462 | Ensembl | 1.36 | 2.19 | 1.64 | 1.68 | -0.33 |
| pisd | NM_001173606.1 | refseq | 1.36 | 2.28 | 1.68 | 1.55 | -0.08 |
| pisd | ENSDARG00000052462 | Ensembl | 1.39 | 1.97 | 1.46 | 1.50 | -0.39 |
| pisd | ENSDARG00000052462 | Ensembl | 1.34 | 2.07 | 1.59 | 1.61 | -0.36 |
| pisd | ENSDARG00000052462 | Ensembl | 1.48 | 2.04 | 1.58 | 1.73 | -0.42 |
| pisd | ENSDARG00000052462 | Ensembl | 1.22 | 1.87 | 1.39 | 1.50 | -0.34 |
| pkn3 | ENSDARG00000079585 | Ensembl | 0.99 | 1.48 | 0.79 | 0.72 | 0.98 |
| plaua | ENSDARG00000039145 | Ensembl | -0.35 | -1.07 | -0.24 | -0.23 | -0.37 |
| plaua | ENSDARG00000039145 | Ensembl | -0.40 | -1.52 | -0.34 | -0.63 | -0.66 |
| plekhg3 | ENSDARG00000096613 | Ensembl | -0.55 | -1.07 | -0.26 | 0.06 | -0.60 |
| plekhg4 | ENSMUSG00000039713 | Ensembl | -0.71 | -1.13 | -0.71 | -0.23 | -0.81 |
| plin2 | ENSDARG00000042332 | Ensembl | 1.03 | 2.35 | 1.14 | 1.17 | 0.82 |
| plin2 | ENSDARG00000042332 | Ensembl | 1.02 | 2.40 | 0.94 | 1.09 | 0.89 |
| plin2 | ENSDARG00000042332 | Ensembl | 0.48 | 1.35 | 0.44 | 0.50 | 0.52 |
| plin2 | ENSDARG00000042332 | Ensembl | 0.49 | 1.24 | 0.40 | 0.26 | 0.57 |
| plin2 | ENSDARG00000042332 | Ensembl | 0.62 | 1.17 | 0.53 | 0.19 | 0.44 |
| plin2 | ENSDARG00000042332 | Ensembl | 0.27 | 1.21 | 0.50 | 0.04 | 0.40 |
| plin2 | ENSDARG00000042332 | Ensembl | 0.30 | 1.33 | 0.45 | 0.21 | 0.34 |
| pltp | ENSDARG00000035768 | Ensembl | 2.37 | 0.53 | 0.31 | 0.42 | 0.03 |
| pltp | ENSDARG00000035768 | Ensembl | 2.68 | 0.39 | 0.34 | 0.63 | 0.10 |
| pltp | ENSDARG00000035768 | Ensembl | 2.63 | 0.55 | 0.75 | 0.12 | 0.74 |
| pnpla3 | ENSDARG00000044086 | Ensembl | 0.99 | 1.17 | 0.21 | 0.00 | 0.54 |
| pnpla7a | ENSDARG00000062986 | Ensembl | 0.47 | 1.06 | 0.18 | 0.13 | -0.59 |
| PPAP2A (1 of 2) | ENSDARG00000079790 | Ensembl | 1.61 | 2.11 | 1.68 | 0.00 | 0.00 |
| PPAP2C (1 of 2) | ENSDARG00000002231 | Ensembl | -0.08 | -1.23 | -0.30 | 0.07 | -0.22 |
| ppargc1a | ENSDARG00000067829 | Ensembl | -0.53 | -0.49 | -1.48 | -0.47 | -0.33 |
| PPP1R16A | ENSDARG00000076980 | Ensembl | -0.70 | -1.36 | -0.58 | -0.57 | -0.05 |
| PPP1R16A | ENSDARG00000076980 | Ensembl | -0.91 | -1.27 | -0.80 | -0.77 | -0.16 |
| praf2 | ENSDARG00000032535 | Ensembl | -0.62 | -0.76 | -0.30 | -0.35 | -0.33 |
| prdm1a | ENSDARG00000002445 | Ensembl | 1.19 | 2.44 | 1.63 | 3.33 | -1.26 |
| prdm1a | ENSDARG00000002445 | Ensembl | 0.00 | 2.83 | 0.00 | 0.00 | 0.00 |
| PRODH (3 of 3) | ENSDARG00000086512 | Ensembl | 0.01 | 0.72 | 1.35 | 0.33 | -0.38 |
| prodha | ENSDARG00000044804 | Ensembl | 0.27 | 0.94 | 1.50 | 0.96 | 0.28 |
| psmc1b | ENSDARG00000043561 | Ensembl | 0.00 | 0.00 | 1.41 | 1.35 | 0.00 |
| psmd11b | ENSDARG00000005134 | Ensembl | 0.36 | 0.69 | 0.47 | 0.68 | 0.28 |
| ptbp1a | ENSDARG00000019362 | Ensembl | -0.15 | -1.45 | -0.37 | -0.05 | -0.31 |
| pycr1 | ENSDARG00000053965 | Ensembl | 0.49 | 0.78 | 0.66 | 0.67 | 0.13 |
| rab11bb | ENSDARG00000090086 | Ensembl | -0.06 | -0.29 | -0.55 | -0.93 | -0.40 |
| rab12 | ENSDARG00000089428 | Ensembl | 0.21 | 0.99 | -0.03 | -0.09 | 0.13 |
| rab20 | ENSDARG00000005049 | Ensembl | 0.53 | 1.25 | 0.40 | 0.47 | 0.09 |
| RAB32 | ENSMUSG00000019832 | Ensembl | 0.47 | 0.89 | 1.01 | 0.53 | 0.37 |
| rad54l2 | ENSDARG00000063031 | Ensembl | 0.56 | 0.91 | 0.86 | 0.23 | 0.56 |
| rad54l2 | ENSDARG00000063031 | Ensembl | 0.54 | 0.67 | 0.92 | 0.10 | 0.29 |
| raf1a | AB204911.1 | nt | 0.56 | 1.51 | 0.89 | 0.96 | 0.38 |
| rarg | EU025716.1 | nt | 2.74 | 2.24 | 0.00 | 2.79 | 1.33 |
| rarg | EU025716.1 | nt | 0.78 | 2.05 | 1.17 | 1.09 | 0.28 |
| rbfox1 | ENSDARG00000014746 | Ensembl | -0.88 | -0.37 | -0.26 | -0.28 | -0.36 |
| RBM6 | ENSDARG00000077060 | Ensembl | 0.00 | 1.04 | 0.00 | 1.18 | 0.00 |
| rela | ENSDARG00000021907 | Ensembl | 0.30 | 1.29 | 0.81 | 1.36 | -0.21 |
| relb | ENSDARG00000086173 | Ensembl | 1.02 | 2.33 | 2.06 | 1.76 | 1.07 |
| relb | ENSDARG00000086173 | Ensembl | 0.83 | 2.24 | 1.74 | 1.63 | 0.83 |
| relb | ENSDARG00000086173 | Ensembl | 1.10 | 2.17 | 1.92 | 1.73 | 0.79 |
| relb | ENSDARG00000086173 | Ensembl | 1.15 | 1.93 | 1.48 | 1.38 | 0.83 |
| relb | ENSDARG00000086173 | Ensembl | 1.01 | 2.06 | 1.49 | 1.51 | 0.82 |
| relb | ENSDARG00000086173 | Ensembl | 0.75 | 1.95 | 1.28 | 1.28 | 0.45 |
| rev3l | ENSDARG00000058801 | Ensembl | -0.78 | -0.97 | -0.44 | -0.43 | -1.04 |
| rfk | ENSDARG00000060522 | Ensembl | 1.77 | 2.61 | 1.76 | 1.56 | 0.38 |
| rifk | NM_001140512.1 | refseq | 1.91 | 2.55 | 1.88 | 1.42 | 0.92 |
| rifk | NM_001140512.1 | refseq | 1.90 | 2.64 | 1.93 | 1.45 | 0.69 |
| rifk | NM_001140512.1 | refseq | 1.86 | 2.73 | 1.82 | 1.51 | 0.53 |
| RIPK2 | ENSG00000104312 | Ensembl | 0.00 | 0.00 | 1.66 | 0.00 | 0.00 |
| rnd3a | ENSDARG00000076799 | Ensembl | 1.02 | 1.75 | 1.40 | 1.16 | 0.28 |
| rnd3a | ENSDARG00000076799 | Ensembl | -0.78 | -0.40 | -1.36 | -0.51 | -0.59 |
| rnf126 | ENSDARG00000088454 | Ensembl | 0.53 | 0.24 | 0.39 | 0.75 | 0.64 |
| rnf170 | ENSG00000120925 | Ensembl | 0.94 | 0.92 | 0.55 | 0.93 | 0.22 |
| Rnf213 | ENSMUSG00000070327 | Ensembl | -0.38 | 0.34 | 0.32 | 1.44 | -0.17 |
| rsad2 | ENSDARG00000004952 | Ensembl | -0.17 | 0.08 | 0.08 | 1.88 | -0.03 |
| rxraa | ENSDARG00000057737 | Ensembl | -0.75 | -0.82 | -0.64 | -0.57 | -0.78 |
| sacs | ENSMUSG00000048279 | Ensembl | -0.82 | -0.35 | 0.04 | 3.01 | -1.36 |
| sacs | ENSMUSG00000048279 | Ensembl | -0.73 | -0.64 | 0.17 | 2.96 | -1.11 |
| sacs | ENSMUSG00000048279 | Ensembl | 0.00 | 0.00 | 0.00 | 2.94 | 0.00 |
| sar1ab | ENSDARG00000033320 | Ensembl | 0.00 | 1.04 | 0.00 | 0.00 | 0.00 |
| sc5d | NM_001140116.1 | refseq | 2.10 | 2.81 | 2.13 | 1.80 | 0.77 |
| scap | ENSDARG00000018096 | Ensembl | 0.63 | 0.99 | 0.52 | 1.15 | -0.08 |
| scinla | ENSDARG00000091639 | Ensembl | -0.49 | -0.41 | -0.15 | -0.11 | -0.82 |
| sdc4 | ENSDARG00000059906 | Ensembl | 1.12 | 4.05 | 2.94 | 1.45 | 0.78 |
| sec14l1 | ENSDARG00000019301 | Ensembl | 0.90 | 1.18 | 1.16 | 0.01 | 0.81 |
| sec23a | ENSDARG00000016636 | Ensembl | 0.56 | 0.78 | 0.54 | 0.53 | 0.43 |
| sec24d | ENSDARG00000045946 | Ensembl | 0.38 | 0.93 | 0.44 | 0.86 | 0.12 |
| sf3b3 | NM_001141594.1 | refseq | 1.01 | 4.53 | 2.69 | 3.15 | -0.13 |
| sgms2 | ENSDARG00000052520 | Ensembl | 0.76 | 1.80 | 1.31 | 1.64 | 0.99 |
| sh2d4b | ENSDARG00000029443 | Ensembl | 0.79 | 1.93 | 1.70 | 2.54 | 0.01 |
| sh2d4b | ENSDARG00000029443 | Ensembl | 1.58 | 2.33 | 1.97 | 2.08 | 0.33 |
| shrprbck1r | ENSDARG00000059871 | Ensembl | 0.39 | 0.74 | 0.35 | 0.28 | 0.03 |
| si:ch211-154o6.6 | ENSDARG00000056379 | Ensembl | 0.28 | 1.56 | 1.00 | 0.61 | 0.53 |
| si:ch211-154o6.6 | ENSDARG00000056379 | Ensembl | 0.56 | 1.76 | 1.15 | 0.98 | 0.55 |
| si:ch211-154o6.6 | ENSDARG00000056379 | Ensembl | 0.88 | 1.94 | 1.41 | 1.45 | 0.51 |
| si:ch211-154o6.6 | ENSDARG00000056379 | Ensembl | 0.89 | 1.89 | 1.43 | 1.41 | 0.50 |
| si:ch211-154o6.6 | ENSDARG00000056379 | Ensembl | 0.88 | 1.92 | 1.31 | 1.45 | 0.45 |
| si:ch211-214c7.4 | ENSDARG00000069595 | Ensembl | -0.51 | -0.49 | -1.37 | -0.08 | -0.40 |
| si:ch211-236p5.3 | ENSDARG00000086418 | Ensembl | -0.86 | -0.82 | -0.47 | -2.29 | -1.73 |
| si:ch211-74m13.1 | ENSDARG00000094952 | Ensembl | 0.85 | 4.24 | 2.33 | 3.02 | 0.23 |
| si:dkey-10o6.2 | ENSDARG00000074628 | Ensembl | -0.82 | -1.24 | -0.64 | -0.71 | -0.01 |
| skia | ENSDARG00000042151 | Ensembl | -0.39 | -1.29 | -0.56 | -0.54 | -0.95 |
| SLC13A5 | ENSG00000141485 | Ensembl | 0.41 | 0.71 | 1.30 | 0.95 | 0.75 |
| SLC13A5 (2 of 2) | ENSDARG00000077691 | Ensembl | 0.82 | 0.71 | 1.08 | 1.10 | 0.61 |
| slc16a6b | ENSDARG00000060246 | Ensembl | 0.40 | 0.84 | 1.12 | 1.62 | 0.56 |
| slc16a6b | ENSDARG00000060246 | Ensembl | 0.44 | 0.81 | 1.20 | 1.47 | 0.46 |
| slc25a28 | ENSDARG00000074297 | Ensembl | 0.00 | 1.26 | 0.00 | 0.48 | 0.00 |
| slc27a4 | ENSDARG00000017047 | Ensembl | 0.00 | 1.03 | 0.66 | 1.13 | 0.19 |
| slc31a1 | ENSDARG00000013961 | Ensembl | 0.93 | 1.59 | 1.62 | 1.52 | 0.29 |
| slc33a1 | ENSDARG00000020085 | Ensembl | 0.40 | 0.85 | 0.45 | 0.78 | 0.24 |
| SLC39A8 (1 of 2) | ENSDARG00000056757 | Ensembl | 0.27 | 0.99 | 0.36 | 1.02 | 0.25 |
| SLC39A8 (2 of 2) | ENSDARG00000087905 | Ensembl | 0.50 | 1.10 | 0.62 | 1.19 | 0.09 |
| slc3a2b | ENSDARG00000037012 | Ensembl | 0.71 | 1.08 | 0.89 | 0.77 | 0.34 |
| slc43a1a | ENSDARG00000037393 | Ensembl | 1.37 | 1.75 | 1.72 | 1.98 | 0.78 |
| slc43a1a | ENSDARG00000037393 | Ensembl | 2.86 | 3.33 | 3.25 | 3.51 | 1.61 |
| slc52 | ENSMUSG00000027463 | Ensembl | 4.86 | 5.50 | 4.32 | 2.55 | 3.49 |
| slc52 | ENSMUSG00000027463 | Ensembl | 4.87 | 5.24 | 4.08 | 2.45 | 3.34 |
| SLC6A16 (1 of 2) | ENSDARG00000007129 | Ensembl | 1.15 | 1.11 | 0.00 | 1.78 | 0.00 |
| smad4 | ENSDARG00000023527 | Ensembl | -0.83 | -1.35 | -0.74 | -0.54 | -0.22 |
| smad6b | ENSDARG00000031763 | Ensembl | -0.43 | -0.73 | -0.44 | -0.15 | -0.32 |
| smad7 | ENSDARG00000016858 | Ensembl | -1.26 | -1.01 | -0.86 | -0.44 | -0.56 |
| smad7 | ENSDARG00000016858 | Ensembl | -0.92 | -0.96 | -0.70 | -0.38 | -0.53 |
| SMCHD1 | ENSG00000101596 | Ensembl | 0.00 | 0.00 | 0.00 | 1.45 | 0.00 |
| smpd5 | ENSDARG00000059811 | Ensembl | 1.01 | 1.43 | 1.80 | 0.51 | 0.46 |
| smpd5 | ENSDARG00000059811 | Ensembl | 0.88 | 1.54 | 1.58 | 0.35 | 0.15 |
| socs2 | ENSDARG00000045557 | Ensembl | 1.22 | 0.00 | 1.62 | 0.00 | 0.00 |
| socs3b | ENSDARG00000026611 | Ensembl | 1.03 | 0.92 | 0.86 | 0.97 | 0.42 |
| sox11a | ENSDARG00000077811 | Ensembl | -0.75 | -1.26 | -0.10 | -0.24 | 0.01 |
| sqstm1 | ENSDARG00000075014 | Ensembl | 0.93 | 1.99 | 1.39 | 1.41 | 0.43 |
| sqstm1 | ENSDARG00000075014 | Ensembl | 0.80 | 1.78 | 1.12 | 1.08 | 0.39 |
| sqstm1 | ENSDARG00000075014 | Ensembl | 0.84 | 1.87 | 1.09 | 1.17 | 0.44 |
| sqstm1 | ENSDARG00000075014 | Ensembl | 0.78 | 1.69 | 1.09 | 1.06 | 0.45 |
| sqstm1 | ENSDARG00000075014 | Ensembl | 0.74 | 1.75 | 0.97 | 1.07 | 0.29 |
| srebf2 | ENSDARG00000063438 | Ensembl | 1.04 | 1.10 | 0.67 | 1.05 | 0.17 |
| srebf2 | ENSDARG00000063438 | Ensembl | 0.95 | 0.96 | 0.50 | 0.90 | 0.01 |
| srebf2 | ENSDARG00000063438 | Ensembl | 1.00 | 1.16 | 0.71 | 0.77 | 0.32 |
| srebf2 | ENSDARG00000063438 | Ensembl | 1.11 | 1.18 | 0.86 | 0.84 | -0.04 |
| srebf2 | ENSDARG00000063438 | Ensembl | 0.99 | 1.13 | 0.32 | 0.73 | 0.06 |
| srebf2 | ENSDARG00000063438 | Ensembl | 1.06 | 0.90 | 0.46 | 0.80 | 0.02 |
| ssh2b | ENSDARG00000077623 | Ensembl | 0.91 | 1.48 | 1.60 | 1.38 | 0.12 |
| ssh2b | ENSDARG00000077623 | Ensembl | 0.97 | 1.30 | 1.59 | 1.19 | 0.27 |
| ssh2b | ENSDARG00000077623 | Ensembl | 1.05 | 1.26 | 1.60 | 1.51 | 0.11 |
| st3gal1 | ENSDARG00000079654 | Ensembl | 0.51 | 1.97 | 1.42 | 0.44 | 0.51 |
| stat2 | ENSDARG00000031647 | Ensembl | 0.40 | 1.19 | 1.00 | 1.19 | 0.37 |
| stat3 | ENSDARG00000022712 | Ensembl | 1.24 | 0.95 | 0.96 | 0.85 | 0.55 |
| steap4 | ENSDARG00000055901 | Ensembl | 1.59 | 1.90 | 1.88 | 1.88 | 0.50 |
| steap4 | ENSDARG00000055901 | Ensembl | 1.60 | 1.95 | 1.96 | 1.83 | 0.56 |
| steap4 | ENSDARG00000055901 | Ensembl | 1.54 | 1.94 | 2.04 | 1.68 | 0.41 |
| steap4 | ENSDARG00000055901 | Ensembl | 1.44 | 1.87 | 1.95 | 1.68 | 0.46 |
| steap4 | ENSDARG00000055901 | Ensembl | 1.27 | 1.82 | 1.79 | 1.73 | 0.32 |
| steap4 | ENSDARG00000055901 | Ensembl | 1.35 | 1.88 | 1.77 | 1.59 | 0.52 |
| steap4 | ENSDARG00000055901 | Ensembl | 1.34 | 1.88 | 1.80 | 1.60 | 0.48 |
| steap4 | ENSDARG00000055901 | Ensembl | 1.60 | 1.93 | 1.98 | 1.79 | 0.42 |
| steap4 | ENSDARG00000055901 | Ensembl | 1.60 | 1.98 | 1.99 | 1.80 | 0.50 |
| steap4 | ENSDARG00000055901 | Ensembl | 1.26 | 1.60 | 1.77 | 1.57 | 0.30 |
| steap4 | ENSDARG00000055901 | Ensembl | 1.48 | 1.82 | 1.88 | 1.62 | 0.38 |
| steap4 | ENSDARG00000055901 | Ensembl | 1.31 | 1.62 | 1.74 | 1.62 | 0.24 |
| steap4 | ENSDARG00000055901 | Ensembl | 1.48 | 1.96 | 1.90 | 1.62 | 0.59 |
| steap4 | ENSDARG00000055901 | Ensembl | 1.50 | 1.74 | 1.84 | 1.64 | 0.29 |
| steap4 | ENSDARG00000055901 | Ensembl | 1.35 | 1.51 | 1.66 | 1.64 | 0.23 |
| steap4 | ENSDARG00000055901 | Ensembl | 1.57 | 1.78 | 1.83 | 1.68 | 0.50 |
| steap4 | ENSDARG00000055901 | Ensembl | 1.46 | 1.80 | 1.98 | 1.68 | 0.52 |
| steap4 | ENSDARG00000055901 | Ensembl | 1.47 | 1.97 | 2.02 | 1.74 | 0.38 |
| steap4 | ENSDARG00000055901 | Ensembl | 1.48 | 1.93 | 2.07 | 1.74 | 0.38 |
| steap4 | ENSDARG00000055901 | Ensembl | 1.42 | 1.69 | 1.84 | 1.77 | 0.16 |
| steap4 | ENSDARG00000055901 | Ensembl | 1.49 | 1.88 | 1.98 | 1.78 | 0.55 |
| steap4 | ENSDARG00000055901 | Ensembl | 1.58 | 1.89 | 1.99 | 1.84 | 0.52 |
| steap4 | ENSDARG00000055901 | Ensembl | 1.01 | 1.44 | 1.48 | 1.23 | 0.42 |
| steap4 | ENSDARG00000055901 | Ensembl | 1.08 | 1.47 | 1.51 | 1.37 | 0.31 |
| steap4 | ENSDARG00000055901 | Ensembl | 1.15 | 1.57 | 1.58 | 1.40 | 0.43 |
| steap4 | ENSDARG00000055901 | Ensembl | 1.57 | 1.73 | 1.81 | 1.71 | 0.64 |
| steap4 | ENSDARG00000055901 | Ensembl | 1.59 | 1.88 | 2.08 | 1.80 | 0.49 |
| steap4 | ENSDARG00000055901 | Ensembl | 1.59 | 1.89 | 1.98 | 1.84 | 0.47 |
| stim2 | ENSDARG00000001776 | Ensembl | 1.32 | 1.72 | 0.82 | 1.42 | 0.62 |
| stk40 | ENSDARG00000060318 | Ensembl | 0.70 | 1.99 | 1.27 | 1.38 | 0.39 |
| stk40 | ENSDARG00000060318 | Ensembl | 0.46 | 1.78 | 1.20 | 1.03 | 0.05 |
| stk40 | ENSDARG00000060318 | Ensembl | 0.93 | 1.87 | 1.27 | 1.50 | 0.44 |
| styx | ENSDARG00000057699 | Ensembl | 0.12 | 0.88 | 0.54 | 0.62 | 0.03 |
| SUCO | ENSMUSG00000040297 | Ensembl | 0.39 | 1.20 | 0.54 | 0.85 | 0.19 |
| syncrip | ENSDARG00000040184 | Ensembl | 0.89 | 1.40 | 0.79 | 1.72 | 0.39 |
| syncripl | ENSDARG00000026723 | Ensembl | 1.12 | 1.72 | 1.11 | 1.62 | 0.58 |
| syncripl | ENSDARG00000026723 | Ensembl | 0.94 | 1.70 | 0.40 | 1.60 | 0.51 |
| SYNPO2 (1 of 2) | ENSDARG00000079675 | Ensembl | -0.23 | 0.83 | 0.37 | 0.20 | -0.08 |
| tbx2b | ENSDARG00000006120 | Ensembl | -0.57 | -0.45 | -1.04 | -0.32 | -0.47 |
| tbx2b | ENSDARG00000006120 | Ensembl | -0.64 | -0.78 | -0.61 | -0.14 | -0.51 |
| tbx2b | ENSDARG00000006120 | Ensembl | -1.20 | -0.68 | -0.54 | -0.29 | -0.97 |
| TDG | ENSGMOG00000019080 | Ensembl | 0.33 | 1.38 | 0.74 | 0.18 | 0.58 |
| tdh | ENSDARG00000002745 | Ensembl | 1.01 | 1.93 | 1.38 | 1.69 | 1.01 |
| tdh | ENSDARG00000002745 | Ensembl | 0.94 | 1.88 | 1.70 | 1.60 | 0.69 |
| tdo2a | ENSDARG00000071429 | Ensembl | -0.35 | -1.39 | -0.30 | -0.31 | 0.24 |
| tescb | ENSDARG00000030839 | Ensembl | -0.16 | 0.98 | 0.49 | 1.97 | -0.03 |
| tfpi2 | ENSDARG00000061351 | Ensembl | 0.27 | -0.08 | 0.71 | 0.83 | -0.02 |
| tial1 | ENSDARG00000009525 | Ensembl | 0.26 | 0.93 | 0.30 | 0.68 | 0.59 |
| tlr21 | ENSDARG00000058045 | Ensembl | -0.17 | 1.58 | 0.85 | 0.56 | -0.20 |
| tlr5b | ENSDARG00000052322 | Ensembl | 2.50 | 4.31 | 3.31 | 3.06 | 1.61 |
| tlr5b | ENSDARG00000052322 | Ensembl | 3.24 | 5.07 | 4.13 | 3.67 | 2.02 |
| tlr5b | ENSDARG00000052322 | Ensembl | 2.52 | 4.24 | 3.27 | 2.84 | 1.52 |
| tnfr14 | NM_001141866.1 | refseq | 1.40 | 3.18 | 2.87 | 3.03 | 0.25 |
| tnfr21 | ENSDARG00000001807 | Ensembl | 0.20 | 1.72 | 1.27 | 0.53 | 0.25 |
| tnip1 | ENSDARG00000015653 | Ensembl | 0.96 | 1.49 | 1.30 | 1.60 | 0.34 |
| tnip1 | ENSDARG00000015653 | Ensembl | 0.63 | 0.95 | 1.00 | 0.53 | 0.35 |
| tnip1 | ENSDARG00000015653 | Ensembl | 0.94 | 1.34 | 1.29 | 1.45 | -0.05 |
| tnip1 | ENSDARG00000015653 | Ensembl | 0.67 | 1.34 | 1.13 | 1.08 | 0.71 |
| TNIP2 | ENSDARG00000074501 | Ensembl | 0.65 | 3.27 | 1.64 | 1.83 | -0.19 |
| tp53 | ENSDARG00000035559 | Ensembl | 0.00 | 0.00 | 0.00 | 1.91 | 0.00 |
| traf2b | ENSDARG00000017812 | Ensembl | 0.29 | 1.30 | 0.90 | 0.81 | 0.04 |
| traf3 | ENSDARG00000022000 | Ensembl | 0.58 | 1.33 | 0.90 | 0.66 | 0.06 |
| trak1 | ENSG00000182606 | Ensembl | -0.06 | -1.31 | -0.53 | -0.46 | -0.23 |
| trim32 | ENSDARG00000076553 | Ensembl | -0.18 | -0.73 | -0.04 | -0.10 | -0.39 |
| trim39 | ENSMUSG00000045409 | Ensembl | -0.87 | -0.63 | -1.19 | -0.28 | -0.68 |
| Tstd1 | ENSMUSG00000091166 | Ensembl | 0.62 | 0.47 | -0.13 | 1.12 | 0.40 |
| txndc11 | ENSDARG00000076938 | Ensembl | 0.64 | 0.00 | 0.00 | 1.60 | 0.00 |
| txndc9 | ENSDARG00000069853 | Ensembl | -1.37 | -1.36 | -0.83 | -1.38 | -0.87 |
| u2af2b | ENSDARG00000011740 | Ensembl | -0.74 | -1.31 | -0.56 | -0.58 | -0.11 |
| ubald1a | ENSDARG00000002362 | Ensembl | 0.37 | 0.77 | 0.37 | 0.24 | 0.28 |
| ubap2 | ENSDARG00000088318 | Ensembl | 0.65 | 0.48 | 0.46 | 1.03 | 0.41 |
| ugt5c1 | ENSDARG00000061444 | Ensembl | -0.20 | -1.13 | -0.30 | -0.80 | -0.30 |
| unc93a | ENSDARG00000041554 | Ensembl | 0.61 | 1.16 | 0.45 | 0.74 | 0.06 |
| uncharacterised | BT045802.1 | nt | -0.43 | -0.27 | 0.17 | 0.02 | 1.27 |
| uncharacterised | BT059612.1 | nt | -0.01 | 0.19 | -0.07 | 0.29 | 0.99 |
| uncharacterised | DQ156150.1 | nt | 0.02 | 0.00 | 0.02 | 0.20 | 0.94 |
| uncharacterised | DQ246664.1 | nt | 0.13 | 0.38 | 0.12 | 0.34 | 1.44 |
| uncharacterised | ENSORLG00000000574 | Ensembl | 0.07 | 0.32 | 0.17 | 0.86 | 1.90 |
| uncharacterised | NM_001141471.1 | refseq | 0.18 | 0.25 | 0.15 | 1.22 | 1.43 |
| uncharacterised |  |  | -0.30 | 0.94 | 1.44 | 1.04 | 2.35 |
| uncharacterised |  |  | 0.51 | 0.49 | 0.56 | 0.24 | 1.28 |
| uncharacterised |  |  | 0.50 | 0.65 | 0.34 | -0.09 | 0.84 |
| uncharacterised | NP_001118004.1 | refseq | -0.19 | -0.24 | 0.96 | 0.96 | 2.07 |
| uncharacterised | EU853449.1 | nt | 0.00 | 0.00 | 0.00 | 0.76 | 1.61 |
| uncharacterised |  |  | 0.00 | 0.00 | 0.56 | 0.00 | 1.26 |
| uncharacterised |  |  | 0.00 | 0.81 | 0.00 | 0.00 | 1.23 |
| uncharacterised |  |  | 0.00 | 0.00 | 0.00 | 0.00 | 1.75 |
| uncharacterised | AC203446.12 | nt | 1.07 | 3.06 | 2.81 | 3.24 | 0.31 |
| uncharacterised | AC203446.12 | nt | 1.06 | 2.33 | 1.27 | 1.48 | 0.28 |
| uncharacterised | AC203446.12 | nt | 1.16 | 2.81 | 2.63 | 2.79 | 0.51 |
| uncharacterised | AF055440.1 | nt | 1.56 | 2.22 | 1.97 | 1.75 | 1.05 |
| uncharacterised | BT047041.1 | nt | -0.02 | 1.71 | 1.27 | 1.55 | 0.36 |
| uncharacterised | BT059080.1 | nt | 1.10 | 2.67 | 1.65 | 1.82 | 0.78 |
| uncharacterised | BT072619.1 | nt | 0.80 | 1.39 | 1.35 | 1.37 | 0.20 |
| uncharacterised | EU221180.1_ | nt | 1.46 | 3.15 | 2.03 | 1.85 | 1.04 |
| uncharacterised | GU129139.1 | nt | 1.33 | 1.78 | 1.67 | 1.38 | 1.29 |
| uncharacterised | GU817337.1 | nt | 1.16 | 2.23 | 2.73 | 2.86 | 0.66 |
| uncharacterised |  |  | 1.17 | 2.44 | 1.74 | 1.45 | 1.25 |
| uncharacterised | EU221177.1 | nt | 2.56 | 2.60 | 2.77 | 2.82 | 1.71 |
| uncharacterised | EU481821.1 | nt | 3.12 | 4.72 | 3.80 | 3.03 | 2.23 |
| uncharacterised | BX511086.5 | nt | 0.92 | 0.83 | 0.85 | 1.03 | 0.65 |
| uncharacterised |  |  | 1.76 | 2.37 | 2.33 | 2.62 | 1.27 |
| uncharacterised | FJ969489.1 | nt | 0.86 | 1.41 | 0.71 | 1.11 | 0.29 |
| uncharacterised | GU294488.1 | nt | 0.35 | 1.55 | 0.57 | 1.81 | 0.13 |
| uncharacterised | AB162343.1 | nt | 0.48 | 0.11 | 0.30 | 1.18 | 0.19 |
| uncharacterised | AC203446.12 | nt | 0.09 | 0.92 | 0.28 | 2.02 | -0.19 |
| uncharacterised | AY493348.1 | nt | 0.54 | 0.56 | 0.74 | 2.10 | -0.73 |
| uncharacterised | BT045214.1 | nt | 0.74 | 0.83 | 0.38 | 1.89 | 1.14 |
| uncharacterised | CAB51372.1 | nt | -0.18 | 0.17 | 0.33 | 1.60 | 0.12 |
| uncharacterised | CAB51372.1 | nt | 0.20 | 0.81 | 0.60 | 1.95 | -0.05 |
| uncharacterised | DQ156149.1 | nt | 0.63 | 1.20 | 0.99 | 1.44 | 0.70 |
| uncharacterised | EU025708.1 | nt | 2.14 | 1.52 | 0.28 | 2.70 | 0.51 |
| uncharacterised | EU816603.1 | nt | 0.76 | 0.12 | 0.99 | 1.26 | 0.62 |
| uncharacterised | GU129140.1 | nt | 1.58 | 0.20 | 1.63 | 2.11 | 1.15 |
| uncharacterised | HM159473.1 | nt | 1.58 | 0.73 | 1.61 | 2.22 | 0.92 |
| uncharacterised |  |  | 0.82 | 0.94 | 1.07 | 2.60 | 1.62 |
| uncharacterised |  |  | 0.64 | 1.26 | 0.67 | 1.46 | 0.51 |
| uncharacterised |  |  | 0.52 | 0.86 | 0.47 | 1.08 | 0.25 |
| uncharacterised | XM_003455175.1 | refseq | 0.33 | 1.60 | 0.21 | 2.44 | -0.15 |
| uncharacterised | XP_003458266.1 | refseq | 1.02 | 0.38 | 0.46 | 1.19 | 0.32 |
| uncharacterised | XP_003458962.1 | refseq | 1.06 | 0.34 | 0.73 | 1.31 | -0.19 |
| uncharacterised | CAB51372.1 | nt | 0.34 | 1.30 | 0.00 | 2.31 | 0.42 |
| uncharacterised | NP_001119851.1 | refseq | 0.68 | 1.09 | 0.00 | 1.14 | 0.47 |
| uncharacterised | NM_001123619.1 | refseq | 0.00 | 0.00 | 0.00 | 2.06 | 0.21 |
| uncharacterised | EU221178.1 | nt | -1.21 | -1.65 | -0.79 | -1.45 | -0.34 |
| uncharacterised | EU481821.1 | nt | -0.70 | -0.81 | -0.68 | -1.14 | -0.19 |
| uncharacterised | HM159471.1 | nt | -1.25 | -1.87 | -0.75 | -1.68 | -0.93 |
| uncharacterised |  |  | -0.13 | -0.90 | -0.58 | -1.17 | -0.57 |
| uncharacterised | FP016154.2 | nt | -1.49 | -1.78 | -0.71 | -1.82 | -0.56 |
| uncharacterised | AB162342.1 | nt | 0.68 | 0.46 | 0.95 | -5.06 | 0.01 |
| uncharacterised | AF232215.1 | nt | -0.24 | -0.22 | -0.25 | -1.59 | 0.21 |
| uncharacterised | EU481821.1 | nt | -0.48 | -0.02 | -0.42 | -0.95 | -0.28 |
| uncharacterised | NM_001165397.1 | refseq | -0.15 | -0.08 | 0.07 | -1.10 | -0.14 |
| uncharacterised | NM_001172281.1 | refseq | -1.03 | -0.25 | 0.00 | -4.84 | -0.62 |
| uncharacterised |  |  | -0.35 | -0.26 | -0.26 | -1.71 | -1.11 |
| uncharacterised |  |  | -0.67 | -0.47 | -0.73 | -1.17 | -0.52 |
| uncharacterised |  |  | -0.62 | -0.68 | -0.53 | -1.39 | -0.46 |
| uncharacterised |  |  | -0.34 | -0.62 | -0.58 | -1.31 | -0.45 |
| uncharacterised |  |  | 0.50 | 0.24 | 0.47 | -6.63 | -0.63 |
| uncharacterised |  |  | 1.34 | 0.66 | 1.09 | -5.37 | 0.40 |
| uncharacterised |  |  | -0.37 | 0.24 | -0.54 | -2.23 | -0.04 |
| uncharacterised |  |  | -0.64 | -0.60 | -0.41 | -1.09 | -0.17 |
| uncharacterised | XP_003448688.1 | refseq | -0.17 | -0.08 | -0.30 | -0.74 | -0.25 |
| uncharacterised | AC203446.12 | nt | 1.48 | 3.50 | 2.48 | 2.17 | 0.86 |
| uncharacterised | BT044988.1 | nt | 0.52 | 2.32 | 1.25 | 1.11 | 0.65 |
| uncharacterised | BT045198.1 | nt | 1.80 | 2.60 | 1.86 | 1.46 | 0.70 |
| uncharacterised | BT045418.1 | nt | 1.77 | 2.50 | 1.74 | 1.40 | 0.68 |
| uncharacterised |  |  | 0.44 | 0.84 | 0.87 | 0.35 | 0.89 |
| uncharacterised |  |  | 0.31 | 0.90 | 0.85 | -0.01 | 0.66 |
| uncharacterised |  |  | 0.53 | 2.00 | 1.30 | 1.32 | 0.70 |
| uncharacterised |  |  | 0.78 | 2.47 | 1.42 | 1.02 | 0.55 |
| uncharacterised |  |  | 1.41 | 2.41 | 1.91 | 1.10 | 0.72 |
| uncharacterised |  |  | 0.23 | 0.91 | 0.66 | 0.73 | 0.07 |
| uncharacterised | XM_003450362.1 | refseq | 0.45 | 1.68 | 1.19 | 0.79 | 0.46 |
| uncharacterised | XM_004553799.1 | refseq | 1.09 | 1.41 | 1.32 | 1.01 | 1.05 |
| uncharacterised | XM_004575248.1 | refseq | 1.11 | 1.75 | 1.31 | 1.36 | 0.81 |
| uncharacterised | AAX28478.2 | nt | 1.05 | 0.88 | 1.62 | 1.05 | 0.76 |
| uncharacterised | BT048706.1 | nt | 1.35 | 2.73 | 3.46 | 3.33 | 0.28 |
| uncharacterised | CX354065.1 | nt | 0.01 | 0.03 | 1.38 | 0.30 | 1.32 |
| uncharacterised | DN047920.1 | nt | 0.51 | 0.65 | 1.25 | 1.32 | 0.87 |
| uncharacterised | DQ246664.1 | nt | 1.69 | 1.96 | 2.04 | 1.99 | 0.68 |
| uncharacterised | DQ246664.1 | nt | 1.76 | 2.24 | 2.06 | 2.00 | 0.64 |
| uncharacterised | DW537532.1 | nt | 1.23 | 1.23 | 1.28 | 0.66 | 0.87 |
| uncharacterised | DY702037.1 | nt | 0.84 | 0.75 | 1.10 | 0.93 | 0.42 |
| uncharacterised | DY729066.1 | nt | 0.82 | 1.05 | 1.24 | 0.82 | 0.79 |
| uncharacterised | DY733338.1 | nt | 0.37 | 1.01 | 1.37 | 1.28 | 0.93 |
| uncharacterised | DY738636.1 | nt | 0.10 | 0.09 | 1.09 | 0.34 | 0.93 |
| uncharacterised | EF210363.1 | nt | 1.68 | 1.83 | 1.95 | 1.74 | 0.81 |
| uncharacterised | EF210363.1 | nt | 1.41 | 1.43 | 1.58 | 1.44 | 0.28 |
| uncharacterised | EG831757.1 | nt | 1.29 | 1.40 | 2.17 | 2.16 | 1.16 |
| uncharacterised | ENSORLG00000004811 | Ensembl | 1.79 | 1.77 | 2.00 | 2.15 | 0.75 |
| uncharacterised | ENSORLG00000004811 | Ensembl | 1.45 | 1.84 | 1.77 | 1.88 | 0.70 |
| uncharacterised | ENSORLG00000004811 | Ensembl | 1.55 | 1.44 | 1.73 | 1.84 | 0.58 |
| uncharacterised | ENSORLG00000015212 | Ensembl | 0.57 | 0.67 | 1.09 | 0.82 | 0.42 |
| uncharacterised | EU025706.1 | nt | 0.64 | 1.25 | 1.33 | 0.91 | 0.65 |
| uncharacterised | EU025708.1 | nt | 1.67 | 1.82 | 2.01 | 1.94 | 0.56 |
| uncharacterised | EU025716.1 | nt | 1.48 | 1.64 | 1.87 | 1.68 | 0.73 |
| uncharacterised | EU025719.1 | nt | 1.79 | 1.98 | 2.14 | 2.07 | 0.70 |
| uncharacterised | EU025719.1 | nt | 0.75 | 1.00 | 1.08 | 0.75 | 0.00 |
| uncharacterised | EU025719.1 | nt | 1.74 | 1.95 | 2.09 | 1.99 | 0.60 |
| uncharacterised | EU221177.1 | nt | 1.82 | 1.82 | 1.94 | 2.03 | 0.66 |
| uncharacterised | EU221180.1 | nt | 1.77 | 1.99 | 2.15 | 1.97 | 0.66 |
| uncharacterised | EV374524.1 | nt | 0.81 | 0.52 | 0.98 | 0.67 | 0.39 |
| uncharacterised | FF845837.1 | nt | 1.37 | 1.10 | 2.04 | 2.26 | 0.98 |
| uncharacterised | FJ356137.1 | nt | 0.35 | 0.71 | 1.04 | 1.20 | 0.66 |
| uncharacterised | GQ505860.1 | nt | 1.69 | 1.66 | 1.78 | 1.88 | 0.54 |
| uncharacterised | GQ925552.1 | nt | 0.42 | 1.72 | 1.71 | 2.01 | 0.87 |
| uncharacterised | GU817336.1 | nt | 1.42 | 1.36 | 1.70 | 1.61 | 0.20 |
| uncharacterised | HM159472.1 | nt | 1.73 | 1.78 | 1.91 | 2.01 | 0.67 |
| uncharacterised | HM159473.1 | nt | 1.77 | 1.80 | 2.05 | 1.98 | 0.52 |
| uncharacterised | HQ287746.1 | nt | 0.83 | 2.16 | 2.13 | 2.02 | 1.21 |
| uncharacterised | NM_001124458.1 | refseq | 1.64 | 1.75 | 1.92 | 1.87 | 0.68 |
| uncharacterised | NM_001146488.1 | refseq | 0.45 | 1.16 | 2.21 | 0.67 | 1.15 |
| uncharacterised | NM_001173968.1 | refseq | 1.73 | 1.82 | 1.92 | 1.98 | 0.48 |
| uncharacterised |  |  | 0.36 | 1.75 | 1.71 | 1.76 | 0.70 |
| uncharacterised | XP_002933173.1 | refseq | 1.10 | 1.91 | 1.55 | 0.89 | 0.47 |
| uncharacterised | NM_001173828.1 | refseq | 1.15 | 1.12 | 1.58 | 1.12 | 0.46 |
| uncharacterised |  |  | 1.15 | 0.43 | 1.04 | 0.70 | 1.21 |
| uncharacterised | XM_004553799.1 | refseq | 1.06 | 0.79 | 1.22 | 0.73 | 0.72 |
| uncharacterised | BT058802.1 | nt | -0.13 | -1.37 | -1.65 | -0.99 | -0.32 |
| uncharacterised | BT125491.1 | nt | -0.07 | -0.19 | -1.30 | -0.23 | 0.18 |
| uncharacterised | CA038505.1 | nt | -0.07 | -0.39 | -1.27 | -0.38 | -0.59 |
| uncharacterised | CA052404.1 | nt | -1.58 | -1.19 | -2.04 | -1.35 | -0.98 |
| uncharacterised | CB510837.1 | nt | -0.74 | -0.54 | -1.10 | -0.62 | -0.56 |
| uncharacterised | CB517258.1 | nt | -1.02 | -0.95 | -1.15 | -0.65 | -0.54 |
| uncharacterised | CR381643.18 | nt | -0.42 | -0.59 | -1.14 | -0.37 | -0.54 |
| uncharacterised | DN047751.1 | nt | -1.69 | -0.33 | -2.83 | -0.51 | -0.43 |
| uncharacterised | DN047751.1 | nt | -1.57 | -0.23 | -3.32 | -0.23 | -0.27 |
| uncharacterised | DY736041.1 | nt | -0.69 | -0.57 | -1.49 | -0.89 | -0.62 |
| uncharacterised | EG818439.1 | nt | -0.27 | -0.75 | -1.32 | -0.28 | -0.11 |
| uncharacterised | EG856747.1 | nt | -0.32 | -0.40 | -1.28 | -0.17 | 0.28 |
| uncharacterised | EG862378.1 | nt | -0.35 | -0.20 | -1.16 | -0.10 | 0.28 |
| uncharacterised | FJ969488.1 | nt | 0.06 | -0.25 | -1.73 | 0.02 | 0.24 |
| uncharacterised | NM_001124249.1 | refseq | 3.17 | 1.65 | -2.56 | -0.31 | 0.31 |
| uncharacterised | NM_001140310.1 | refseq | -0.68 | -0.67 | -1.09 | -0.33 | -0.30 |
| uncharacterised | NM_001141267.2 | refseq | -1.03 | -0.73 | -1.27 | -0.49 | -0.41 |
| uncharacterised | NM_001141481.1 | refseq | -0.18 | 0.06 | -1.13 | -0.41 | -0.06 |
| uncharacterised | NM_001173941.1 | refseq | -0.21 | -0.27 | -1.12 | 0.53 | 0.21 |
| uncharacterised |  |  | -0.61 | -0.91 | -1.44 | -1.01 | -0.57 |
| uncharacterised |  |  | -0.92 | -1.09 | -1.33 | -1.11 | 0.09 |
| uncharacterised |  |  | -1.38 | -0.45 | -3.18 | -0.45 | -0.41 |
| uncharacterised |  |  | -1.23 | -0.46 | -1.75 | 0.07 | -0.14 |
| uncharacterised |  |  | -0.69 | -0.05 | -1.09 | 0.12 | -0.23 |
| uncharacterised | XP_001923568.1 | refseq | -1.26 | -0.14 | -2.85 | -0.48 | -0.43 |
| uncharacterised | AB204911.1 | nt | 0.78 | 2.30 | 1.12 | 1.12 | 0.42 |
| uncharacterised | AC203446.12 | nt | 0.71 | 1.97 | 1.15 | 0.65 | 0.50 |
| uncharacterised | AC203446.12 | nt | 0.40 | 1.27 | 0.30 | 0.62 | 0.09 |
| uncharacterised | AC203446.12 | nt | 0.48 | 1.21 | 0.44 | 0.68 | 0.03 |
| uncharacterised | ACI66788.1 | nt | 0.49 | 1.38 | 0.88 | 0.47 | 0.48 |
| uncharacterised | ACI68549.1 | nt | 0.85 | 2.43 | 1.59 | 0.77 | 0.92 |
| uncharacterised | ACN10093.1 | nt | 1.06 | 1.92 | 1.36 | 0.78 | 0.83 |
| uncharacterised | ACN10793.1 | nt | 0.64 | 1.44 | 0.51 | 0.74 | 0.61 |
| uncharacterised | BT045054.1 | nt | 0.58 | 1.22 | 0.87 | 0.83 | 0.04 |
| uncharacterised | BT045136.1 | nt | 0.15 | 0.64 | 0.27 | 0.37 | 0.27 |
| uncharacterised | BT048122.1 | nt | 0.62 | 1.35 | 1.06 | 0.68 | 0.16 |
| uncharacterised | BT059080.1 | nt | 0.73 | 1.82 | 1.05 | 1.04 | 0.72 |
| uncharacterised | BT059080.1 | nt | 0.49 | 1.48 | 0.40 | 0.56 | 0.68 |
| uncharacterised | BT059080.1 | nt | 0.34 | 1.59 | 0.61 | 0.74 | 0.65 |
| uncharacterised | BT071883.1 | nt | 1.05 | 2.41 | 1.42 | 1.66 | 0.27 |
| uncharacterised | BT072281.1 | nt | 0.10 | 0.93 | 0.38 | 0.63 | 0.14 |
| uncharacterised | BT072361.1 | nt | 0.74 | 1.95 | 1.07 | 0.98 | 0.89 |
| uncharacterised | BT072377.1 | nt | 0.66 | 1.07 | 0.76 | 0.92 | 0.49 |
| uncharacterised | BT072377.1 | nt | 0.61 | 1.42 | 0.69 | 1.01 | 0.51 |
| uncharacterised | BT072598.1 | nt | 0.97 | 2.15 | 0.89 | 0.99 | 0.99 |
| uncharacterised | CB484574.1 | nt | 0.34 | 1.02 | 0.90 | 1.00 | 0.68 |
| uncharacterised | DQ778606.1 | nt | 0.66 | 1.52 | 0.79 | 0.80 | 0.46 |
| uncharacterised | DQ778606.1 | nt | 0.91 | 2.05 | 1.02 | 1.05 | 0.49 |
| uncharacterised | DQ778606.1 | nt | 0.42 | 1.46 | 0.72 | 0.80 | 0.06 |
| uncharacterised | DQ778606.1 | nt | 0.35 | 1.32 | 0.62 | 0.82 | 0.21 |
| uncharacterised | DW535658.1 | nt | 0.56 | 1.27 | 1.17 | 0.40 | 0.55 |
| uncharacterised | DW538554.1 | nt | 0.53 | 1.41 | 0.42 | 0.77 | 0.47 |
| uncharacterised | DW557959.1 | nt | 0.26 | 1.57 | 0.78 | 0.50 | 0.35 |
| uncharacterised | DW564872.1 | nt | 0.23 | 1.04 | -0.20 | 0.75 | 0.48 |
| uncharacterised | DW564872.1 | nt | 0.20 | 1.14 | 0.24 | 0.99 | -0.20 |
| uncharacterised | DW565550.1 | nt | 0.71 | 1.11 | -0.01 | 0.47 | 0.34 |
| uncharacterised | DY719183.1 | nt | 0.99 | 1.90 | 1.04 | 0.16 | 0.39 |
| uncharacterised | DY724465.1 | nt | 1.61 | 3.63 | 1.60 | 2.08 | 0.84 |
| uncharacterised | DY729630.1 | nt | 0.48 | 1.77 | 1.05 | 1.09 | 0.30 |
| uncharacterised | EF467296.1 | nt | 1.53 | 2.35 | 1.87 | 1.50 | 1.12 |
| uncharacterised | EG778722.1 | nt | 0.04 | 0.86 | 0.61 | 0.54 | 0.16 |
| uncharacterised | EG787053.1 | nt | 1.06 | 1.89 | 1.22 | 1.39 | 0.44 |
| uncharacterised | EG858211.1 | nt | 0.83 | 1.62 | 0.76 | 1.05 | 0.40 |
| uncharacterised | EG876529.1 | nt | 0.63 | 1.01 | 0.68 | 0.39 | 0.41 |
| uncharacterised | EG891444.1 | nt | 0.18 | 1.59 | 0.54 | 0.98 | 0.20 |
| uncharacterised | EG939846.1 | nt | 0.42 | 1.33 | 0.81 | 0.58 | 0.25 |
| uncharacterised | ENSONIG00000020760 | Ensembl | 0.31 | 0.88 | 0.34 | 0.46 | 0.26 |
| uncharacterised | ENSONIG00000020760 | Ensembl | 0.43 | 0.95 | 0.33 | 0.44 | 0.21 |
| uncharacterised | ENSONIG00000020760 | Ensembl | 0.50 | 1.48 | 0.61 | 0.70 | 0.23 |
| uncharacterised | ENSONIG00000020760 | Ensembl | 0.21 | 0.81 | 0.18 | 0.33 | 0.16 |
| uncharacterised | ENSONIG00000020760 | Ensembl | 0.41 | 1.17 | 0.50 | 0.60 | 0.15 |
| uncharacterised | ENSORLG00000004337 | Ensembl | 0.38 | 1.01 | 0.40 | 0.50 | 0.25 |
| uncharacterised | ENSORLG00000004811 | Ensembl | 0.99 | 1.09 | 0.83 | 0.83 | 0.57 |
| uncharacterised | EU025709.1 | nt | -0.08 | 1.09 | 0.16 | -0.06 | 0.13 |
| uncharacterised | EU025714.1 | nt | 0.38 | 1.35 | 0.71 | 0.77 | 0.55 |
| uncharacterised | EU025715.1 | nt | 0.97 | 1.74 | 1.21 | 0.53 | 0.55 |
| uncharacterised | EU025715.1 | nt | 0.76 | 1.70 | 1.03 | 0.80 | 0.20 |
| uncharacterised | EU025717.1 | nt | 0.73 | 1.72 | 1.21 | 0.56 | 0.37 |
| uncharacterised | EU221176.1 | nt | -0.17 | 0.70 | 0.25 | -0.11 | 0.34 |
| uncharacterised | EU221178.1 | nt | 0.56 | 1.28 | 0.69 | 0.75 | 0.33 |
| uncharacterised | EU481821.1 | nt | 0.55 | 0.90 | 0.39 | 0.34 | 0.02 |
| uncharacterised | EU621898.1 | nt | 0.68 | 1.10 | 0.54 | 0.62 | 0.20 |
| uncharacterised | FM207658.1 | nt | 0.28 | 2.01 | 1.15 | 0.71 | 0.86 |
| uncharacterised | GQ505859.1 | nt | -0.13 | 3.45 | -0.10 | 0.24 | 0.08 |
| uncharacterised | GU129139.1 | nt | 0.68 | 2.55 | 1.57 | 1.34 | 0.10 |
| uncharacterised | GU129140.1 | nt | 0.17 | 1.22 | 0.50 | 0.63 | -0.01 |
| uncharacterised | HM159473.1 | nt | 1.06 | 1.95 | 1.39 | 0.91 | 0.62 |
| uncharacterised | HM208332.1 | nt | 0.19 | 1.15 | 0.55 | 0.43 | 0.34 |
| uncharacterised | NM_001139612.1 | refseq | 0.40 | 1.11 | 0.62 | 0.53 | 0.29 |
| uncharacterised | NM_001139997.1 | refseq | 0.56 | 1.39 | 0.65 | 0.52 | 0.45 |
| uncharacterised | NM_001160619.1 | refseq | 0.63 | 1.27 | 0.10 | 0.49 | 0.49 |
| uncharacterised | NM_001173566.1 | refseq | 0.64 | 1.48 | 0.75 | 0.73 | 0.16 |
| uncharacterised |  |  | 0.70 | 1.10 | 0.44 | 0.42 | 0.51 |
| uncharacterised |  |  | -0.56 | 1.74 | 0.65 | 0.48 | 0.17 |
| uncharacterised | NR_030020.1 | refseq | 0.51 | 1.30 | 0.91 | 0.96 | 0.66 |
| uncharacterised | U58910.1 | nt | 0.50 | 2.53 | 1.15 | 1.49 | -0.55 |
| uncharacterised | FJ969489.1 | nt | 1.20 | 1.63 | 0.93 | 1.26 | 0.68 |
| uncharacterised |  |  | 1.19 | 2.02 | 1.06 | 1.33 | 0.55 |
| uncharacterised | AB162342.1 | nt | -0.47 | -0.86 | -0.78 | 0.08 | 0.00 |
| uncharacterised | ABV31710.1 | nt | -0.36 | -0.86 | -0.37 | -0.47 | -0.21 |
| uncharacterised | AC203456.8 | nt | -0.44 | -1.06 | -1.03 | -0.08 | 0.20 |
| uncharacterised | BAB55662.1 | nt | 0.05 | -0.88 | -0.15 | 0.12 | -0.43 |
| uncharacterised | BAB55662.1 | nt | 0.20 | -0.93 | -0.16 | 0.09 | -0.38 |
| uncharacterised | BAB55662.1 | nt | 0.30 | -0.77 | -0.20 | 0.10 | -0.26 |
| uncharacterised | BAB55662.1 | nt | 0.20 | -1.32 | -0.45 | -0.22 | -0.35 |
| uncharacterised | BAB55662.1 | nt | 0.24 | -0.77 | -0.10 | 0.28 | -0.18 |
| uncharacterised | BT044936.1 | nt | -0.43 | -1.05 | -0.42 | -0.34 | -0.16 |
| uncharacterised | BT057777.1 | nt | 0.22 | -1.03 | -0.32 | -0.66 | 0.05 |
| uncharacterised | BT059209.1 | nt | -0.87 | -1.09 | -0.38 | -0.40 | -0.30 |
| uncharacterised | BT059282.1 | nt | -1.59 | -1.53 | -0.67 | -1.25 | -0.65 |
| uncharacterised | BT059667.1 | nt | -0.03 | -0.86 | 0.17 | -0.09 | 0.03 |
| uncharacterised | BT072251.1 | nt | -0.28 | -1.04 | -0.04 | 0.05 | 0.39 |
| uncharacterised | BT072255.1 | nt | -1.77 | -1.87 | -0.97 | 0.31 | 0.14 |
| uncharacterised | BT072255.1 | nt | -1.50 | -1.93 | -1.69 | 0.32 | 0.00 |
| uncharacterised | CA063502.1 | nt | -0.52 | -1.13 | -0.72 | -0.75 | -0.33 |
| uncharacterised | CR318614.7 | nt | -0.54 | -1.04 | -0.15 | -0.16 | 0.21 |
| uncharacterised | DQ025547.1 | nt | -0.10 | -0.84 | 0.03 | -0.41 | -0.17 |
| uncharacterised | DQ156151.1 | nt | -0.55 | -2.13 | -1.45 | -0.55 | -1.65 |
| uncharacterised | DQ246664.1 | nt | -0.34 | -0.96 | 0.10 | 0.11 | -0.10 |
| uncharacterised | DW570469.1 | nt | -0.64 | -1.28 | -0.38 | -0.39 | -0.30 |
| uncharacterised | DY700139.1 | nt | -0.81 | -2.19 | -0.17 | -0.67 | -0.70 |
| uncharacterised | DY714549.1 | nt | -0.36 | -0.92 | -0.47 | -0.37 | -0.42 |
| uncharacterised | DY722844.1 | nt | -0.99 | -1.08 | -0.82 | -0.67 | -0.29 |
| uncharacterised | DY730167.1 | nt | -1.44 | -2.48 | -0.57 | -1.17 | -0.45 |
| uncharacterised | EF427377.1 | nt | -0.32 | -1.25 | 0.11 | -0.24 | 0.15 |
| uncharacterised | EF467300.1 | nt | -0.83 | -1.50 | -0.32 | -0.14 | -0.11 |
| uncharacterised | EG760823.1 | nt | -0.55 | -1.04 | -0.39 | -0.23 | -0.63 |
| uncharacterised | EG795246.1 | nt | -0.70 | -1.19 | -0.24 | -0.75 | -0.53 |
| uncharacterised | EG818374.1 | nt | -0.46 | -0.89 | -0.50 | -0.33 | 0.00 |
| uncharacterised | EG860955.1 | nt | -0.57 | -0.93 | -0.20 | -0.31 | -0.14 |
| uncharacterised | EG930769.1 | nt | -0.40 | -1.47 | -0.49 | -0.44 | -0.42 |
| uncharacterised | ENSGACG00000002729 | Ensembl | -0.69 | -1.91 | 0.75 | -0.45 | -0.33 |
| uncharacterised | ENSORLG00000017674 | Ensembl | -0.50 | -1.06 | -0.01 | -0.23 | -0.15 |
| uncharacterised | ENSORLG00000017674 | Ensembl | -0.22 | -0.87 | 0.12 | -0.05 | 0.09 |
| uncharacterised | EU025716.1 | nt | -0.23 | -0.87 | -0.46 | -0.22 | -0.61 |
| uncharacterised | EU025717.1 | nt | -0.62 | -1.18 | -0.76 | -0.26 | -0.19 |
| uncharacterised | EU025717.1 | nt | -0.26 | -1.06 | 0.13 | -0.22 | 0.05 |
| uncharacterised | EU025717.1 | nt | -0.35 | -0.97 | -0.07 | 0.03 | -0.24 |
| uncharacterised | EU221177.1 | nt | -0.84 | -2.15 | -1.20 | -0.75 | -0.21 |
| uncharacterised | EU221179.1 | nt | -0.61 | -1.06 | 0.13 | -0.04 | -0.68 |
| uncharacterised | EV394677.1 | nt | -0.27 | -0.94 | -0.31 | -0.25 | 0.13 |
| uncharacterised | EV394848.1 | nt | -0.18 | -0.97 | -0.53 | 0.03 | -0.30 |
| uncharacterised | FJ969488.1 | nt | -1.05 | -1.07 | -0.61 | -0.75 | -0.71 |
| uncharacterised | FJ969490.1 | nt | -0.26 | -0.99 | 0.00 | -0.23 | 0.02 |
| uncharacterised | FJ969490.1 | nt | -0.16 | -1.08 | 0.13 | -0.16 | -0.01 |
| uncharacterised | GQ505860.1 | nt | -0.99 | -1.55 | 0.23 | -0.52 | -0.80 |
| uncharacterised | GQ505860.1 | nt | -0.86 | -1.68 | 0.51 | -0.59 | -0.54 |
| uncharacterised | GQ505860.1 | nt | -0.66 | -1.74 | 0.72 | -0.58 | -0.18 |
| uncharacterised | GQ505860.1 | nt | -0.59 | -1.88 | 0.47 | -0.39 | -0.54 |
| uncharacterised | GQ505860.1 | nt | -0.71 | -1.53 | 0.69 | -0.25 | -0.22 |
| uncharacterised | GQ925642.1 | nt | -0.14 | -1.02 | 0.21 | -0.21 | 0.10 |
| uncharacterised | GQ925642.1 | nt | -0.29 | -1.01 | -0.09 | -0.06 | 0.05 |
| uncharacterised | HM159473.1 | nt | -0.33 | -1.15 | -0.01 | -0.15 | -0.61 |
| uncharacterised | HQ287745.1 | nt | -0.31 | -1.38 | 0.12 | -0.21 | 0.18 |
| uncharacterised | NM_001129986.1 | refseq | -2.39 | -4.82 | 1.58 | -1.20 | 2.77 |
| uncharacterised |  |  | -0.96 | -1.11 | -0.41 | -0.35 | -0.69 |
| uncharacterised |  |  | -0.73 | -1.51 | -0.55 | -0.27 | -0.63 |
| uncharacterised |  |  | -2.01 | -2.00 | -0.72 | -1.69 | -0.94 |
| uncharacterised |  |  | -1.55 | -1.99 | -1.24 | -1.89 | -0.79 |
| uncharacterised |  |  | -0.43 | -1.19 | -0.69 | -0.49 | -0.42 |
| uncharacterised |  |  | -0.38 | -0.92 | -0.52 | -0.35 | -0.10 |
| uncharacterised |  |  | 0.01 | -0.76 | -0.30 | -0.22 | -0.09 |
| uncharacterised | NP_957363.1 | refseq | -0.22 | -0.90 | -0.55 | -0.21 | -0.05 |
| uncharacterised | NR_029981.1 | refseq | -0.44 | -0.81 | -0.60 | -0.23 | -0.43 |
| uncharacterised | XP_003458662.1 | refseq | 0.20 | -1.03 | -0.28 | -0.28 | -0.05 |
| uncharacterised | BT059787.1 | nt | -1.22 | -0.32 | 0.11 | -0.68 | -0.30 |
| uncharacterised | BX571969.5 | nt | -1.11 | -0.13 | -0.51 | -0.52 | -0.08 |
| uncharacterised | CT033841.18 | nt | -0.98 | -0.48 | -0.52 | -0.59 | 0.02 |
| uncharacterised | DQ156150.1 | nt | -1.06 | -0.85 | -0.97 | -0.12 | -0.08 |
| uncharacterised | DQ849941.1 | nt | -0.80 | -0.39 | -0.42 | -0.14 | -0.19 |
| uncharacterised | DW582826.1 | nt | -1.98 | -0.95 | -0.80 | -0.69 | -0.71 |
| uncharacterised | DY701683.1 | nt | -1.42 | -1.00 | -0.46 | -0.51 | 0.20 |
| uncharacterised | DY710134.1 | nt | -0.92 | -0.69 | -0.43 | -0.50 | 0.03 |
| uncharacterised | EG801741.1 | nt | -1.62 | -0.49 | -0.08 | -0.96 | 0.11 |
| uncharacterised | EU025718.1 | nt | -1.22 | -0.73 | -0.81 | -0.62 | -0.30 |
| uncharacterised | EU481821.1 | nt | -1.05 | -0.57 | -0.11 | -0.30 | 0.26 |
| uncharacterised | EV392641.1 | nt | -1.88 | -0.47 | -0.23 | -0.59 | -0.80 |
| uncharacterised | HM159471.1 | nt | -0.94 | -0.92 | -0.99 | -0.57 | -0.59 |
| uncharacterised | HQ287746.1 | nt | -0.93 | -0.47 | -0.48 | -0.45 | -0.32 |
| uncharacterised | JN755268.1 | nt | -1.25 | -0.87 | -0.89 | -0.29 | -0.09 |
| uncharacterised |  |  | -1.64 | -0.82 | -0.38 | -0.10 | -0.83 |
| uncharacterised | ACN11142.1 | nt | 1.14 | 0.90 | 1.01 | 0.81 | 0.92 |
| uncharacterised | BX649540.7 | nt | 3.22 | -0.63 | 1.54 | -1.18 | 0.02 |
| uncharacterised | CA046791.1 | nt | 1.27 | 1.07 | 1.16 | 0.66 | 0.72 |
| uncharacterised | CAZ39956.1 | nt | 0.92 | 0.21 | 0.26 | 0.74 | 0.69 |
| uncharacterised | DW571201.1 | nt | 1.66 | 0.75 | 0.61 | 0.62 | 0.18 |
| uncharacterised | BT059485.1 | nt | 0.00 | 2.06 | 0.00 | 0.62 | 0.27 |
| uncharacterised | GU817335.1 | nt | 0.38 | 0.73 | 1.41 | 0.00 | 0.92 |
| uncharacterised | BT045014.1 | nt | 0.94 | 0.00 | 1.11 | 0.00 | 1.10 |
| uncharacterised | GU129140.1 | nt | 0.21 | 0.00 | 1.34 | 0.00 | 0.56 |
| uncharacterised | EG766368.1 | nt | 0.27 | 1.77 | 1.23 | 0.00 | 0.52 |
| uncharacterised | CB508615.1 | nt | 0.68 | 1.05 | 0.00 | 0.00 | 0.05 |
| uncharacterised |  |  | 0.00 | 1.22 | 0.00 | 0.00 | 0.52 |
| uncharacterised | AF004739.1 | nt | 2.13 | 4.38 | 3.42 | 6.00 | 0.00 |
| uncharacterised |  |  | 1.59 | 3.01 | 2.08 | 2.72 | 0.00 |
| uncharacterised | AC203446.12 | nt | 0.89 | 0.00 | 0.70 | 1.16 | 0.00 |
| uncharacterised | EU481821.1 | nt | 0.00 | 0.00 | 0.77 | 1.12 | 0.00 |
| uncharacterised | BT047517.1 | nt | 0.00 | 0.93 | 0.00 | 1.02 | 0.00 |
| uncharacterised |  |  | 0.00 | 1.11 | 0.00 | 2.22 | 0.00 |
| uncharacterised |  |  | 0.00 | 0.00 | 0.00 | 2.70 | 0.00 |
| uncharacterised | XP_002740391.1 | refseq | 0.00 | 0.00 | 0.00 | 1.69 | 0.00 |
| uncharacterised | CB494419.1 | nt | 1.26 | 2.43 | 2.84 | 2.20 | 0.00 |
| uncharacterised | EF210363.1 | nt | 0.47 | 1.23 | 1.51 | 1.54 | 0.00 |
| uncharacterised | ACN58700.1 | nt | 0.72 | 0.00 | 1.35 | 1.08 | 0.00 |
| uncharacterised | AC203446.12 | nt | 0.27 | 1.64 | 0.86 | 0.72 | 0.00 |
| uncharacterised |  |  | 1.22 | 1.21 | 0.00 | 0.94 | 0.00 |
| uncharacterised | EU025706.1 | nt | 0.00 | 1.79 | 0.00 | 0.74 | 0.00 |
| uncharacterised | HQ287747.1 | nt | 0.00 | 1.20 | 0.00 | 1.72 | 0.00 |
| uncharacterised |  |  | 0.77 | 2.51 | 1.69 | 0.00 | 0.00 |
| uncharacterised | ENSONIG00000015881 | Ensembl | 1.73 | 0.00 | 2.26 | 0.00 | 0.00 |
| uncharacterised | BT059181.1 | nt | 0.00 | 0.00 | 1.69 | 0.00 | 0.00 |
| uncharacterised | BT072122.1 | nt | 0.00 | 0.00 | 1.80 | 0.00 | 0.00 |
| uncharacterised | EG850879.1 | nt | 0.00 | 0.00 | 2.46 | 0.00 | 0.00 |
| uncharacterised | ENSGACG00000017205 | Ensembl | 0.00 | 0.00 | 1.77 | 0.00 | 0.00 |
| uncharacterised | GQ505860.1 | nt | 0.00 | 0.00 | 2.79 | 0.00 | 0.00 |
| uncharacterised | GU817336.1 | nt | 0.00 | 0.00 | 2.69 | 0.00 | 0.00 |
| uncharacterised | NM_001140220.1 | refseq | 0.00 | 0.00 | 1.60 | 0.00 | 0.00 |
| uncharacterised | EU025719.1 | nt | 1.37 | 0.00 | 1.11 | 0.00 | 0.00 |
| uncharacterised | HM159473.1 | nt | 1.43 | 0.00 | 0.77 | 0.00 | 0.00 |
| uncharacterised | EG877986.1 | nt | 1.37 | 1.99 | 0.00 | 0.00 | 0.00 |
| uncharacterised | BT071996.1 | nt | 0.00 | 1.13 | 0.00 | 0.00 | 0.00 |
| uncharacterised | DW561968.1 | nt | 0.00 | 1.18 | 0.00 | 0.00 | 0.00 |
| uncharacterised | DW573624.1 | nt | 0.00 | 2.21 | 0.00 | 0.00 | 0.00 |
| uncharacterised | NM_001140385.1 | refseq | 0.00 | 1.05 | 0.00 | 0.00 | 0.00 |
| uncharacterised |  |  | 0.00 | 1.36 | 0.00 | 0.00 | 0.00 |
| URGCP (1 of 3) | ENSDARG00000078731 | Ensembl | -0.08 | -0.02 | 0.10 | 1.67 | -0.74 |
| USP2 (2 of 3) | ENSDARG00000087495 | Ensembl | 0.78 | 0.55 | -0.05 | 2.20 | 0.17 |
| vcam1 | ENSDARG00000062479 | Ensembl | -0.36 | -0.50 | -0.58 | -1.01 | -0.18 |
| vps37a | ENSDARG00000017119 | Ensembl | 0.59 | 0.79 | 0.61 | 0.42 | 0.50 |
| vtg1 | ENSDARG00000092233 | Ensembl | 2.73 | 1.60 | 1.08 | 1.71 | 1.51 |
| wasb | ENSDARG00000026350 | Ensembl | -0.56 | -0.57 | -0.52 | -0.20 | -1.39 |
| wbscr27 | ENSDARG00000069507 | Ensembl | -0.56 | -1.01 | -0.34 | -0.37 | -0.11 |
| xbp1 | ENSDARG00000035622 | Ensembl | 0.84 | 1.04 | 0.75 | 0.59 | 0.21 |
| XM_004550138.1 | XM_004550138.1 | refseq | 1.45 | 3.20 | 2.89 | 3.20 | 0.52 |
| XM_004550138.1 | XM_004550138.1 | refseq | 1.65 | 3.26 | 3.06 | 3.50 | 0.41 |
| XM_004551111.1 | XM_004551111.1 | refseq | 0.34 | 1.96 | 1.07 | 0.68 | 0.60 |
| XM_004551111.1 | XM_004551111.1 | refseq | 0.40 | 2.00 | 1.13 | 0.78 | 0.50 |
| zbtb21 | ENSDARG00000043285 | Ensembl | 0.69 | 1.27 | 1.04 | 0.66 | 0.24 |
| zdhhc23b | ENSDARG00000003899 | Ensembl | 0.87 | 0.83 | 0.37 | 0.69 | 0.72 |
| zgc:152863 | ENSDARG00000069338 | Ensembl | 1.31 | 2.10 | 1.66 | 1.05 | 1.42 |
| zgc:162608 | ENSDARG00000069375 | Ensembl | 2.10 | 1.03 | 1.33 | 0.16 | 1.65 |
| zgc:162608 | ENSDARG00000069375 | Ensembl | 2.11 | 1.54 | 0.78 | 1.44 | 1.60 |
| zgc:162608 | ENSDARG00000069375 | Ensembl | 2.07 | 1.03 | 1.29 | 0.20 | 1.61 |
| zgc:162608 | ENSDARG00000069375 | Ensembl | 2.00 | 1.02 | 1.32 | 0.19 | 1.55 |
| zgc:162608 | ENSDARG00000069375 | Ensembl | 1.99 | 0.91 | 1.27 | 0.08 | 1.60 |
| zgc:162608 | ENSDARG00000069375 | Ensembl | 1.85 | 0.79 | 1.06 | -0.07 | 1.43 |
| zgc:162608 | ENSDARG00000069375 | Ensembl | 2.03 | 1.02 | 1.24 | 0.07 | 1.52 |
| zmym4 | ENSDARG00000035823 | Ensembl | -0.06 | -1.02 | -0.24 | -0.16 | -0.25 |

**Reference list:**

Huang DW, Sherman BT, Lempicki RA (2008) Systematic and integrative analysis of large gene lists using DAVID bioinformatics resources. Nature Protocols 4: 44-57.
